# Substituted Aryl Benzylamines as Potent and Selective Inhibitors of 17β-Hydroxysteroid Dehydrogenase Type 3

**DOI:** 10.3390/molecules26237166

**Published:** 2021-11-26

**Authors:** Nigel Vicker, Helen V. Bailey, Joanna M. Day, Mary F. Mahon, Andrew Smith, Helena J. Tutill, Atul Purohit, Barry V. L. Potter

**Affiliations:** 1 Medicinal Chemistry, Department of Pharmacy and Pharmacology and Sterix Ltd., University of Bath, Claverton Down, Bath BA2 7AY, UK; vickern@yahoo.co.uk (N.V.); helen.driver@admin.cam.ac.uk (H.V.B.); asmith@cresset-group.com (A.S.); 2 Oncology Drug Discovery & Women’s Health Group, Department of Endocrinology & Metabolic Medicine & Sterix Ltd., Imperial College London, London W2 1NY, UK; joannamaryberry@gmail.com (J.M.D.); h.tutill@ucl.ac.uk (H.J.T.); atul.purohit1@gmail.com (A.P.); 3 Department of Chemistry, University of Bath, Claverton Down, Bath BA2 7AY, UK; M.F.Mahon@bath.ac.uk; 4 Medicinal Chemistry & Drug Discovery, Department of Pharmacology, University of Oxford, Mansfield Road, Oxford OX1 3QT, UK

**Keywords:** prostate cancer, synthesis, dehydrogenase, homology modelling, chiral chromatography, X-ray crystallography

## Abstract

17β-Hydroxysteroid dehydrogenase type 3 (17β-HSD3) is expressed at high levels in testes and seminal vesicles; it is also present in prostate tissue and involved in gonadal and non-gonadal testosterone biosynthesis. The enzyme is membrane-bound, and a crystal structure is not yet available. Selective aryl benzylamine-based inhibitors were designed and synthesised as potential agents for prostate cancer therapeutics through structure-based design, using a previously built homology model with docking studies. Potent, selective, low nanomolar IC_50_ 17β-HSD3 inhibitors were discovered using *N*-(2-([2-(4-chlorophenoxy)phenylamino]methyl)phenyl)acetamide (**1**). The most potent compounds have IC_50_ values of approximately 75 nM. Compound **29**, *N*-[2-(1-Acetylpiperidin-4-ylamino)benzyl]-*N*-[2-(4-chlorophenoxy)phenyl]acetamide, has an IC_50_ of 76 nM, while compound **30**, *N*-(2-(1-[2-(4-chlorophenoxy)-phenylamino]ethyl)phenyl)acetamide, has an IC_50_ of 74 nM. Racemic *C*-allyl derivative **26** (IC_50_ of 520 nM) was easily formed from **1** in good yield and, to determine binding directionality, its enantiomers were separated by chiral chromatography. Absolute configuration was determined using single crystal X-ray crystallography. Only the *S*-(+)-enantiomer (**32**) was active with an IC_50_ of 370 nM. Binding directionality was predictable through our in silico docking studies, giving confidence to our model. Importantly, all novel inhibitors are selective over the type 2 isozyme of 17β-HSD2 and show <20% inhibition when tested at 10 µM. Lead compounds from this series are worthy of further optimisation and development as inhibitors of testosterone production by 17β-HSD3 and as inhibitors of prostate cancer cell growth.

## 1. Introduction

**Figure 1:**
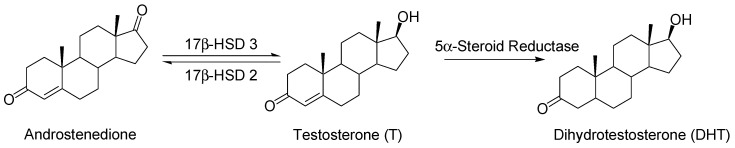
The role of 17β-HSD Type 3 in androgen synthesis.

With over 1,275,000 cases reported annually globally [1], prostate cancer is the second most fatal cancer in males and still represents a therapeutic area with a greatly unmet medical need, despite the huge advances of the last decade. The androgens testosterone (T) and dihydrotestosterone (DHT) (Figure 1) are hormones that play an important role in the development of prostate cancer, while the androgen receptor (AR) is central to the clinical management of prostate cancer [2]. Currently, used therapies in the US include the 5α-reductase inhibitor Finasteride; anti-androgens Bicalutamide (Casodex^TM^), Flutamide (Eulexin^TM^), and Nilutamide (Nilandron^TM^); and more recently the 17,20-lyase inhibitor Abiraterone.

Another interesting potential therapeutic approach relates to the 17β-hydroxysteroid dehydrogenases (17β-HSDs), a family of enzymes that catalyse the oxido-reduction of 17β-alcohol or 17-keto groups on steroids using NAD(P)H or NAD(P) as a cofactor [3]. These enzymes catalyse the final step in male and female sex hormone biosynthesis. In humans, fifteen 17β-HSD isozymes have been characterised, and types 1–4 as well as 6–15 belong to the short-chain dehydrogenase/reductase (SDR) family [4,5,6]. The 17β-HSD5 isozyme, however, belongs to the Aldo-Keto Reductase (AKR) family [7]. Specifically, 17β-Hydroxysteroid dehydrogenase type 3 (17β-HSD3) catalyses the formation of testosterone from androstenedione [8], while the type 2 enzyme (17β-HSD2) catalyses the reverse reaction, oxidising testosterone to the weaker androgen androstenedione (Figure 1). Yet, 17β-HSD3 inhibition is still an unexploited potential therapeutic modality.

17β-HSD3 is expressed at high levels in the testes and seminal vesicles and is also present in prostate tissue, suggesting its potential involvement in both gonadal and non-gonadal testosterone biosynthesis. The enzyme uses NADPH as a cofactor in the reductive direction and is a protein of 310 amino acids with an apparent molecular mass of ~35 kDa [9]. It is a microsomal enzyme, bound through the *N*-terminal transmembrane domain to the endoplasmic reticulum [4]. Expression of 17β-HSD3 mRNA increases over 30-fold in cancerous prostate biopsies [10], and inhibition of 17β-HSD3 may, therefore, reduce prostate tumour growth. The role of 17β-HSD3 in testosterone biosynthesis thus makes this enzyme an attractive molecular target for small molecule inhibitors against prostate cancer, but in any design strategy it is essential that such inhibitors are selective for the inhibition of 17β-HSD3 over 17β-HSD2. 

**Figure 2:**
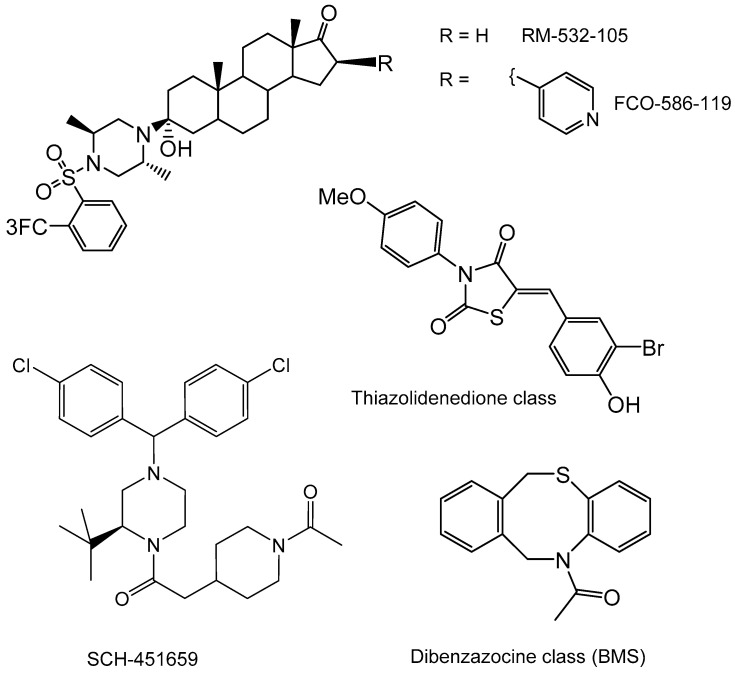
Established steroidal and non-steroidal inhibitors of 17β-HSD3.

The 17β-HSD3 research area was recently reviewed, including the published details of steroidal or non-steroidal inhibitors [11,12]. Earlier known potent inhibitors, the non-steroidal SCH-451659 [13] and a Bristol-Myers Squibb (BMS) *N*-acetyl tetrahydrodibenzazocine lead [14], are shown in Figure 2. Steroidal RM-532-105 is an androsterone derivative substituted at position 3β, developed from SAR investigations. A dansyl-labelled inhibitor for optical imaging was reported from the same series. IC_50_ values of 5 nM and 13 nM, respectively, were observed using whole HEK-293 cells and LNCaP cells, respectively, both over-expressing human 17β-HSD3 [15]. Given subcutaneously to rats in vivo, this compound also significantly decreased the plasma levels of T and DHT. A 16β-picolyl derivative, FCO-586-119 (Figure 2), was also recently reported [16]. Another orally bioavailable non-steroidal *N*-aryl substituted thiazolidenedione 17β-HSD3 inhibitor class was also reported [15] (Figure 2), where delivery using an easily removed phenolic phosphate ester prodrug was adopted.

**Figure 3:**
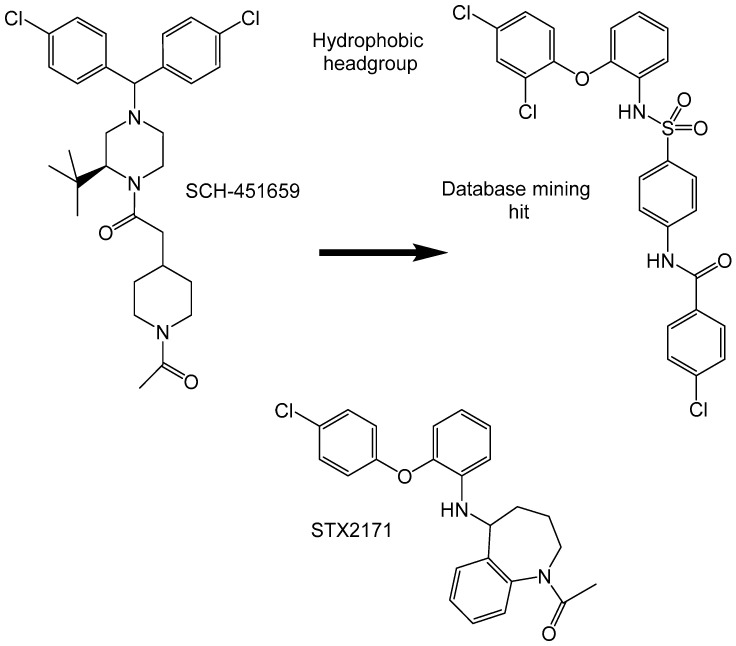
Database mining hit from SCH-451659 and resulting lead STX2171.

We reported earlier on our own initial efforts in this area, applying a structure-based drug design approach encompassing pharmacophore generation, database mining (Maybridge), homology model construction and docking studies [17], as well as using SCH-451659 [13] as a lead compound [18,19] (Figure 3). 

STX2171 (Figure 3) is a non-androgenic, weakly cytotoxic (IC_50_ = 200 nM for 17β-HSD3 inhibition) lead compound and has an encouraging in vitro profile and initial in vivo results [20,21]. It is also selective for the inhibition of 17β-HSD3 over 17β-HSD2. STX2171 showed potent inhibition of androstenedione-stimulated tumour growth in a mouse model [20,21], thus giving good precedent to the work reported in this paper. 

We now report the full details of a related, but novel, aryl benzylamine inhibitor series (see Supplementary Materials for spectroscopic, HPLC and X-ray data and other details) with details of selectivity, androgenicity and cytotoxicity, with some members exceeding the selective potency of STX2171 against 17β-HSD3 in vitro.

## 2. Results and Discussion

Our previously constructed 17β-HSD3 homology model was integral to this current study [17], enabling conclusions to be made about the large size and shape of the active site. A homology model based on the sequence of human 17β-HSD3 has also been reported and used in docking studies of RM-532-105 and the 16β-picolyl derivative FCO-586-119 [16]. It was anticipated that the enzyme would tolerate large compounds, either in length or bulk. Scope for further substitution on a flexible linker may allow additional interactions in the active site as outlined by our substitution strategy below. 

**Figure 4:**
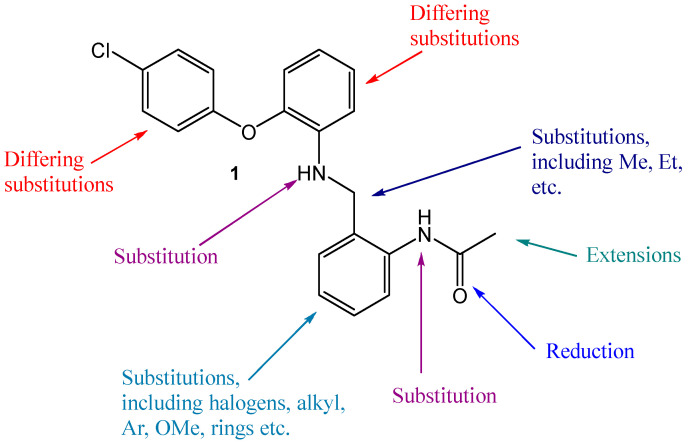
Scope of substitution on lead novel benzylamine, compound **1**.

We planned to build an SAR from the various analogues synthesised based upon *N*-(2-([2-(4-chloro-phenoxy)phenylamino]methyl)phenyl)acetamide **1** (Figure 4). It was envisaged to synthesise and screen a cohort of compounds to identify lead compounds that could be modified in this new benzylamine-linked series. The diphenyl ether hydrophobic head here, such as in STX2171, was derived from the original database mining hit. 

Extending the length of the inhibitor molecule may help to realign the compound in the active site, so that it can interact with other hydrophobic regions and the cofactor. SCH-451659 illustrated the importance of this region, when the activity was greatly increased with the incorporation of a large hydrophobic group on the piperidine linker, such as *t*-butyl [13]. This implies that there is spatial flexibility in this region of the active site, so filling this space is likely to be beneficial for improving potency and selectivity.

### 2.1. Synthesis and Evaluation of Initial Targets

**Scheme 1:**
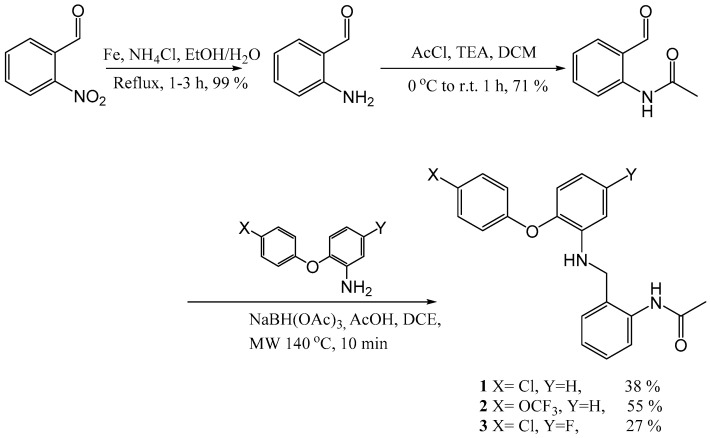
Synthesis of novel benzylamine linked targets, compounds **1**, **2** and **3.** MW, microwave.

The synthesis of the first target was initiated from commercially available 2-nitrobenzaldehyde, which was reduced to the corresponding primary amine using iron powder (Scheme 1). This intermediate can decompose due to reactions between the amine and the aldehyde functional groups, so it must either be stored in the freezer or quickly used in the next step, which was acetylation of the amine to stabilise the intermediate. The aldehyde can then be reacted with the desired differing headgroup aniline using the standard reductive amination conditions [22] to produce the final compounds. 

Compound **1** shows good inhibitor activity with an IC_50_ value of 0.9 μM, as a promising lead compound and a starting point for further optimisation (full biological data of all compounds is reported in Table 9).

### 2.2. In Silico Analysis of the Initial Targets

**Figure 5:**
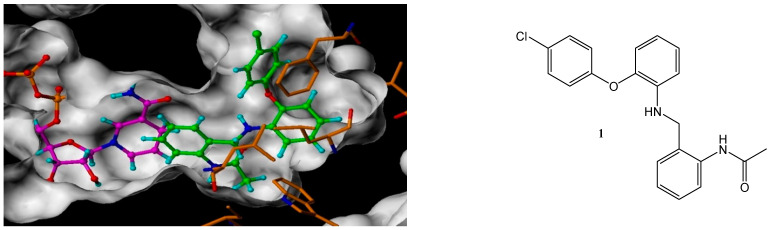
Compound **1** (green) docked in the 17β-HSD3 homology model showing interactions with the cofactor (cyan) and key amino acid residues (brown). These colours are consistent throughout.

Figure 5 shows compound **1** docked into our 17β-HSD3 homology model within the active site, very differently from the amide and amine linked compounds of our previous work [17]. The diphenyl ether hydrophobic headgroup lies further within the hydrophobic pocket; thus, the alignment of the molecule is such that the central aromatic ring lies towards the cofactor at the base of the active site. 

We had previously observed that the acetyl group carbonyl is usually orientated towards the cofactor. However, in this case the acetyl group appears to fit the active site well, but there is significant space surrounding the molecule to be investigated and to improve activity. Although docking is only a guideline, it was useful to generate hypotheses in early lead optimisation.

### 2.3. Modifications to the Benzylamine Template

There are various regions of **1** that can be modified with substituents to allow a more detailed SAR to be constructed. Substitution around the hydrophobic headgroup was explored with the preparation of **2,** with a 4-trifluoromethoxy-substituted hydrophobic diphenyl ether headgroup, and **3** with a 5-fluoro-headgroup substituent (Scheme 1). However, these showed lower biological activity than **1** (Table 9). 

### 2.4. Modification of the Benzylamine Template: Substitutions on the Central Aromatic Ring

The docking of **1** (Figure 5) shows that there is a large amount of space surrounding the central aromatic ring. This area also lies towards the cofactor and, therefore, there may be scope for potential interactions in this region. A wide range of substitutions was planned to extend our SAR, including halogens, heterocycles, naphthyl, alkyl and alkoxy groups. 

**Scheme 2:**
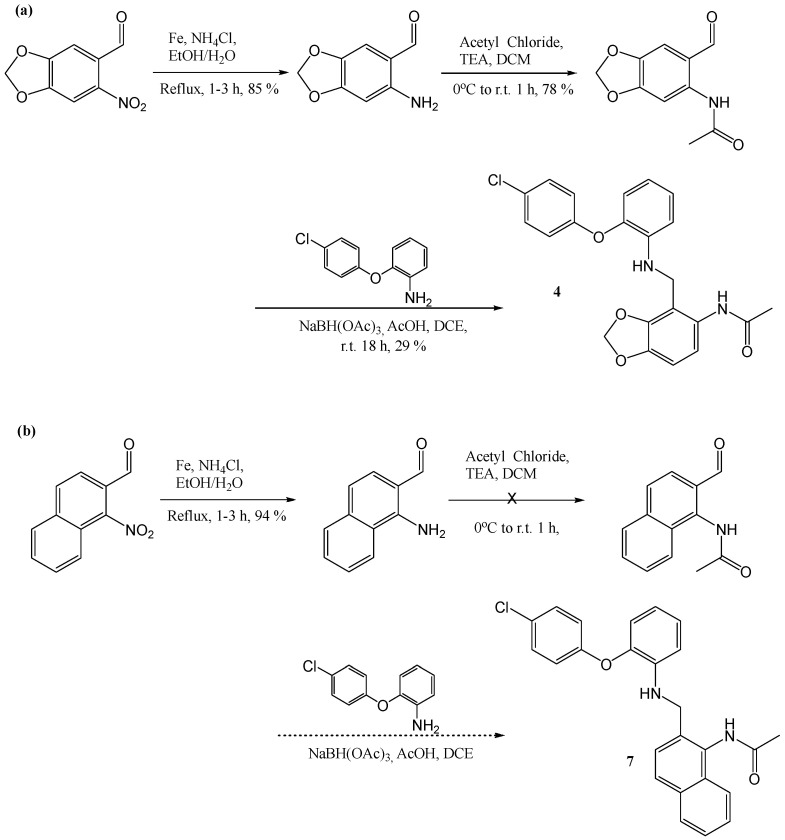
(**a**) Synthesis of **4**; (**b**) attempted synthesis of the naphthyl analogue **7**.

Initially, the synthesis of these compounds was attempted using the same route as for **1**. In the case of the 4,5-methylenedioxy substitution, this was successful and **4** was synthesised effectively (Scheme 2a). It should be noted that the final reductive amination step was carried out at room temperature, as use of the microwave methodology, so successful elsewhere, led to decomposition. However, when the same route was applied to naphthyl analogue **7** (Scheme 2b), it was unsuccessful as the intermediate was unstable. Even when used immediately or stored in the freezer, this compound decomposed before acetylation could occur. 

**Scheme 3:**
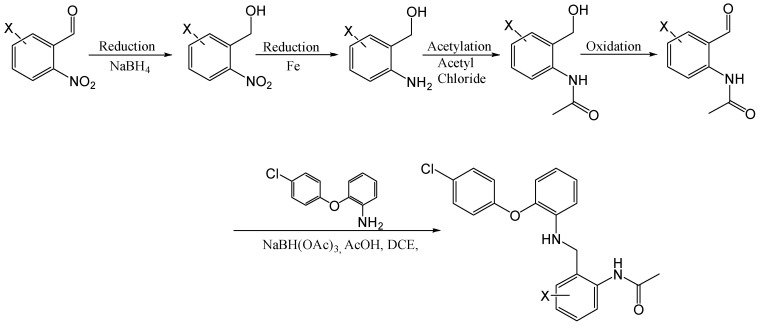
New synthetic route to compounds with a substituted central aromatic ring.

Investigations were initiated to find a synthetic route that was suitable for a wider range of substituents. The availability of starting materials meant that the 4,5-methylenedioxy substituent was used in these investigations. In the new route (Scheme 3), reductive amination would be the first step and the nitro group would then be reduced and acetylated, thus avoiding the previously problematic intermediate. However, reduction of the nitro group failed and the desired product was not isolated. The iron-based reduction conditions caused the amine bond in the molecule to break, and only the diphenyl ether hydrophobic headgroup aniline was isolated. This occurs as the intermediate contains a benzyl-protected amine type moiety, thus indicating that the amine bond might be sensitive to benzyl deprotection conditions, in this case iron reduction, so a more robust universal route was required.

**Scheme 4:**
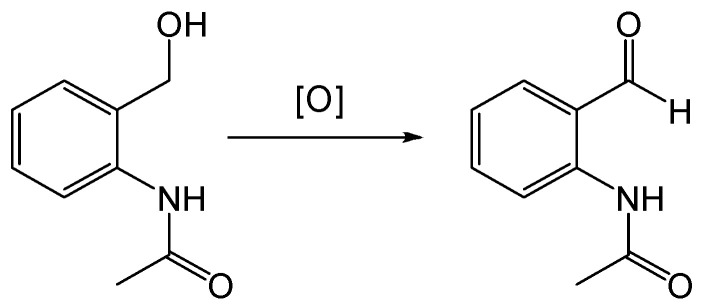
Alcohol oxidations of 2-Acetamidobenzyl.

We developed a new route to the substituted aromatic group from 2-nitrobenzyl alcohol (Scheme 3). The route required optimisation before it could be used to prepare a range of compounds with varying substitution patterns around the central aromatic ring. The reduction of the aldehyde to the alcohol was carried out using NaBH_4_ in quantitative yield over 2 h. The reduction of the nitro group and the subsequent acetylation were carried out successfully using the standard iron reduction conditions from the literature [23]. The oxidation of the alcohol to the aldehyde (Scheme 4) required more detailed investigation. There are many available reagents for this oxidation, and some were tested to find the most suitable for this application (see Table 1).

**Scheme 5:**
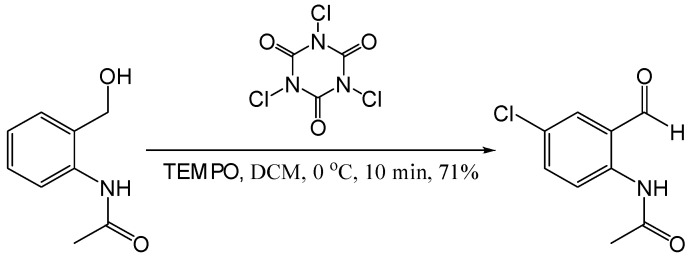
Formation of incorrect chloro-substituted product.

A method from the literature was attempted [24], in which trichloroisocyanuric acid was used to activate TEMPO for the mild and chemo-selective oxidation of alcohols (Table 1, Entry 1). However, for this application it is not a suitable reagent as it also caused a substitution of chlorine in the ring (Scheme 5). Although 5-chloro substitution is one of the targets in this series, this is not a desirable method as only the one analogue can be prepared in this way. It was hoped that the trichloroisocyanuric acid could be removed from this reaction, and TEMPO could act alone. However, after 3 days, only the starting alcohol was isolated (Table 1, Entry 2).

Four successful methods for this oxidation were discovered (Table 1, Entries 3–6), all producing the correct product in similar yields. DMP was chosen as the method of choice (Table 1, Entry 5) because it gave the most reproducible results, did not require anhydrous conditions and is easy to remove at the end of the reaction through a simple aqueous work up plus standard flash chromatography.

**Scheme 6:**
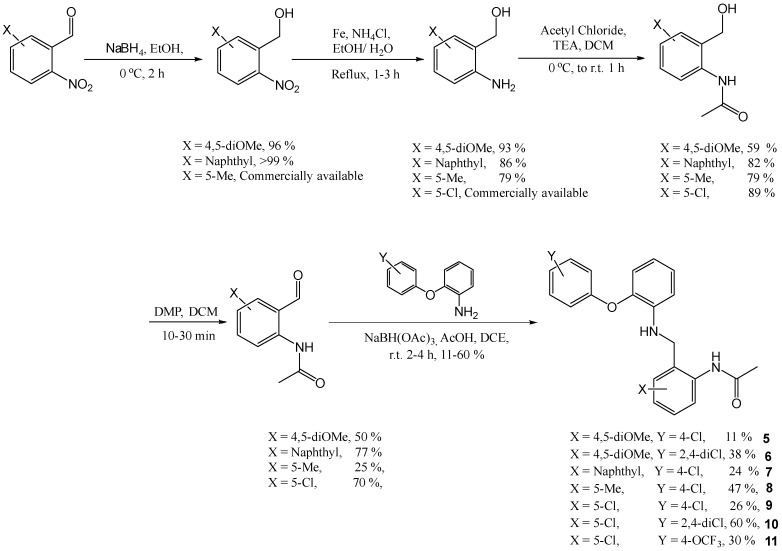
Synthetic route for analogues with various substitutions around the central aromatic ring.

DMP was used to oxidise the respective alcohols in the synthesis of all targets in this series (Scheme 6). Unfortunately, the reliable results observed in the test reactions were not conserved, and the yields varied from 25–77%. There are many different, commercially available starting materials with differing substitution patterns, either as the 2-nitrobenzaldehyde, the 2-nitrobenzylalcohol or the 2-aminobenzylalcohol. The availability of the commercial starting materials dictated at which step in the route synthesis began. For example, the 4,5-dimethoxy substitution pattern was only available as the 2-nitroaldehyde, so the full 5-step synthesis was required. In contrast, the 5-chloro-substitution was available as the 2-aminobenzylalcohol, so only the final 3 steps were required.

This synthetic route led to a wide range of analogues of **1**, e.g., **5** and **6** with various central aromatic ring substitutions. Eight novel compounds were prepared in this series and are listed in Table 2, along with their biological results. Biological results for all compounds evaluated are also summarised at the end (Table 9). IC_50_ values were calculated for compounds showing >70% inhibition at 10 μM, and compounds showing <20% inhibition at 10 μM are classed as inactive (IA).

Generally, 4-substituents (Y below) are well tolerated on the diphenyl ether hydrophobic head, even OCF_3_. It can be seen that substitutions around the central ring are also allowed and the analogue with methylenedioxy substitution (**4**) has good activity. However, steric clashes were predicted for the di-methoxy analogues **5** and **6** and activity was lost. Although these functionalities were tolerated, they do not lead to increased activity and **1** was still the most active compound in this series. Compound **11** with Y = 4-trifluoromethoxy and X = 5-chloro has good activity, indicating these medium-sized groups at these positions are tolerated in the active site.

Observations from docking studies with these compounds showed that some substitutions, such as chlorine in compound **9**, do not seem to affect the overall position of the molecule within the active site, when compared to **1**. This might explain why little difference in inhibitory activity is observed between compounds **1** and **9**.

**Figure 6:**
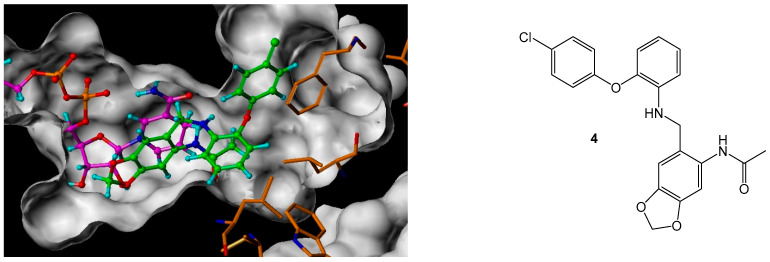
Compound **4** with a 4,5-methylenedioxy substituent docked into the homology model.

Conversely, a considerable change in alignment is observed in the docking study of the 4,5-methylenedioxy-substituted compound **4**, as the compound is no longer aligned in the same way as **1**. It appears that the 4,5-methylenedioxy group interacts strongly with the cofactor within the active site. This is strong enough to realign the molecule, as the hydrophobic headgroup is no longer in a hydrophobic pocket. It could be assumed that this change in alignment would greatly alter the biological activity. However, the result for **4** is not substantially different to that for **1**, showing these substitutions to be well tolerated and further secondary binding interactions such as π-stacking are likely, see Figure 6. Residues in the hydrophobic pocket include Val221, plus Tyr229 and Trp192 can π-stack with aryl groups on inhibitors to improve activity. The methylenedioxyphenyl analogue (**4**) is active with the dimethoxy compound (**5**) being inactive, indicating that steric clashes likely lower activity in the latter. A similar observation is noted for the methylenedioxyphenyl chloro-compound (**4**) being active and the dimethoxy dichloro-compound (**6**) being inactive. The naphthalene and *C*-benzyl-linked analogues lose activity, thus confirming that a steric constraint in the hydrophobic pocket is important for tight binding and good activity. 

### 2.5. Modifications to the Benzylamine Template with Substitutions on the Linker Nitrogen and Acetamide

**Scheme 7:**
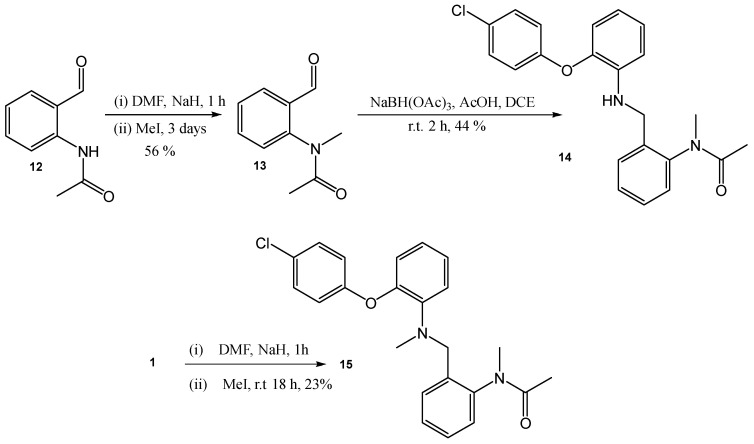
Synthesis of *N*-methyl acetamide **14** and the di-methylated product **15**.

So far, the main potential hydrogen bond donors are the nitrogen atoms in the amine and the amide bond. Alkylation of either or both nitrogen atoms will help to determine which NH, if any, acts as a hydrogen-bond donor in enzyme-inhibitor interactions. The synthesis of a series of *N*-methylated compounds was thus initiated. To methylate the amide nitrogen alone, it was necessary to carry out the reaction on the *N*-(2-formylphenyl) acetamide intermediate before the reductive amination with the headgroup. This methylated intermediate was isolated in 56% yield and was reacted with the headgroup to produce compound **14** in 44% yield (Scheme 7) with an IC_50_ of 460 nM.

Another method of methylation was investigated to synthesise the desired compounds directly from **1** and, by using different ratios of sodium hydride and methyl iodide, it was hoped that the three desired methylated analogues could be synthesised. However, it was not straightforward, as the desired product was not always obtained as predicted. Methylation of both the amine and the amide nitrogen simultaneously was possible by this route, giving a 23% yield of the weakly active dimethylated compound, *N*-(2-acetylamino-benzyl)-*N*-[2-(4-chloro-phenoxy)-phenyl]-acetamide **15** (see Table 9). The major product of this reaction was the mono-*N*-methylated product **14** previously synthesised. 

**Scheme 8:**
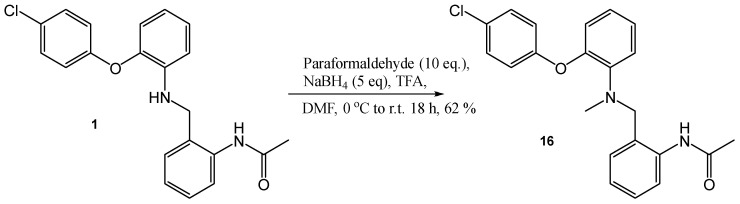
Synthesis **16** with an *N*-methylated linker.

The synthesis of the mono-methylated amine analogue (**16**) was also attempted. Unfortunately, this did not produce the desired product and only the mono-methylated amide **14** was formed. Successful *N*-methylation was achieved directly from compound **1** (Scheme 8) using paraformaldehyde to give the *N*-formyl product in situ, which was then reduced with NaBH_4_/TFA to form the desired product **16** in a 62% yield [28]. Compound **16** was inactive, possibly due to the conformational change caused by the combination of the *N*-methyl and *N*-acetyl groups. 

**Scheme 9:**
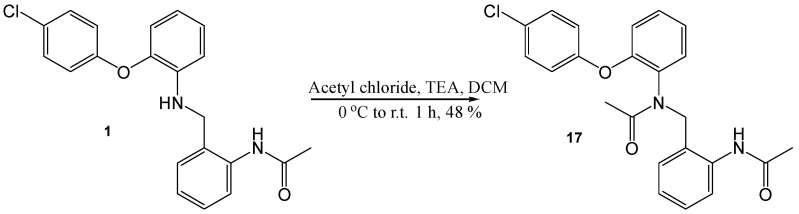
Synthesis of compound **17** with acetyl groups on the side chain and linker.

Introduction of an *N*-acetyl group on the benzylamine linker gave the inactive *N*-diacetylated compound **17** (Scheme 9) in a one-step procedure also from **1** in 48% yield. The inactivity of **17** could also be explained by reasons similar to those of compound **16.**

### 2.6. Modifications to the Benzylamine Template to Give 2, 3 and 4 N-Acetyl-Substituted Analogues

Docking studies of **1** indicated that activity might be improved by allowing it to take up an alignment, such as the original amide or amine linked compounds. It was hypothesised that this may be achieved by migrating the *N*-acetamide around the central aromatic ring, from the original 2 position of **1** to the 3 or 4 positions.

**Scheme 10:**
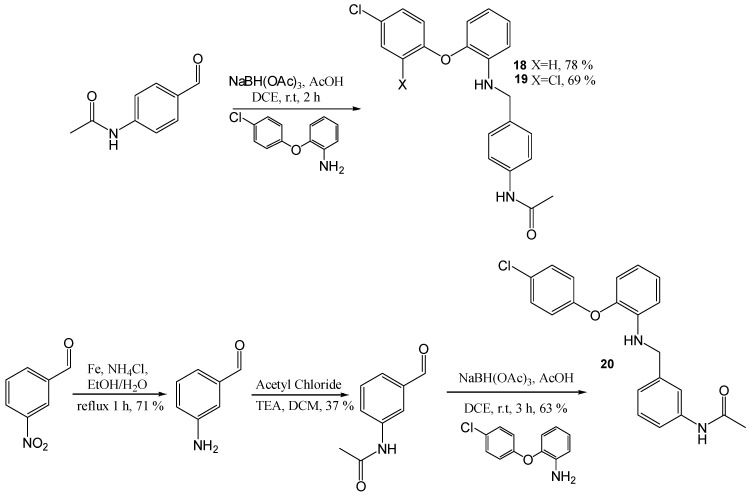
Synthesis of **18**, **19** and **20** give the respective 3- and 4-acetyl substituents on the central aromatic ring.

Compound **1** is the 2-substituted compound, so, therefore, there were two further compounds that required synthesis in this series for an SAR to be established (Scheme 10). For the 4-substituted analogue, the 4-*N*-acetylated aldehyde was commercially available, thus meaning that the final compound, **18**, could be synthesised in one step by reductive amination with the 4-chloro-diphenyl ether aniline and was isolated in 78% yield. The 2,4-dichloro analogue was also synthesised to give **19** in an isolated yield of 69%. For the 3-substituted analogue, the 3-*N*-acetylated aldehyde required synthesis from the commercially available 3-nitroaldehyde. Reduction of the nitro group with the aldehyde present and then acetylation of the primary amine gave the required product. This synthetic route is low yielding due to the decomposition of the 3-aminoaldehyde intermediate. However, the desired product was obtained and was reacted with the 4-chloro-substituted headgroup aniline to produce **20** in 63% yield.

With the synthesis of **18**, **19** and **20** results show that migration of the *N*-acetyl group does not lead to increased inhibitory activity (see Table 3 and series); the compound with the 2-acetyl substitution, i.e., **1** was still the most active.

Migration of the *N*-acetamido group to both the 3 and 4 positions causes the compounds to be pulled towards the cofactor, thus meaning that the hydrophobic headgroup is no longer orientated in the hydrophobic pocket of the enzyme. These changes in alignment may explain the reasons for the lower activity observed with these compounds compared to the 2-substituted acetamide, **1**. Compound **19** is inactive probably due to the second chloro-substituent altering the conformation of the diphenylether headgroup. 

### 2.7. Modifications to the Benzylamine Template, Synthesis and Docking of Extended Analogues

**Scheme 11:**
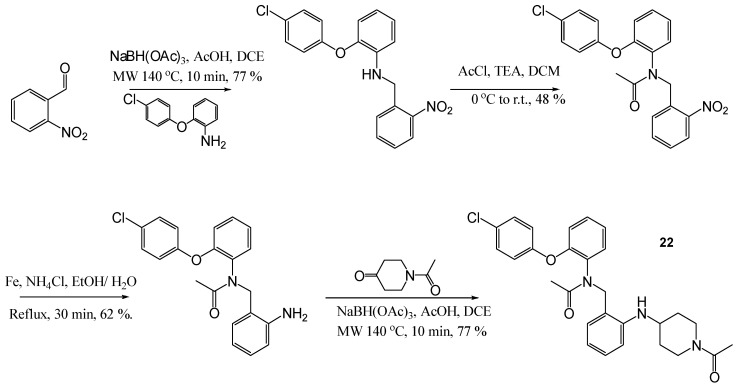
Synthetic route to the extended 4-acetyl-*N*-piperidinyl analogue **22**.

Different regions of the template have been explored and so far show no improvement in activity over **1**. In this benzylamine series, it is not clear from the docking studies whether extension will help, as the compound is aligned differently. It is possible that an extension will cause the molecule to be realigned within the active site, and this may affect activity. The synthesis of extended benzylamine-linked analogues was investigated, and the first example is the *N*-piperidyl derivative **22**, depicted in Scheme 11.

The synthetic route was different to those already used. Since the point of diversification is desired to be the final step in the synthesis, common intermediates are used where possible. Hence, the first step was the reductive amination between the headgroup aniline and 2-nitrobenzaldehyde. This was carried out using our enhanced microwave-assisted conditions [20] and the product was isolated in an excellent 77% yield (Scheme 11). The aniline nitrogen was protected to ensure that it did not interfere with any reactions at the primary amine at a later stage in the synthetic route. The first choice for this protection was an *N*-*t*-Boc protecting group; however, only starting material was isolated from this reaction with di-*t*-butyl-dicarbonate, so another method was required. The small acetyl group was chosen, and formation of the protected amine was successful in 48% yield and iron reduction gave the aniline in 62% yield. The reductive amination with 1-acetyl-4-piperidone produced the desired compound **22** in a good yield of 77%.

**Scheme 12:**
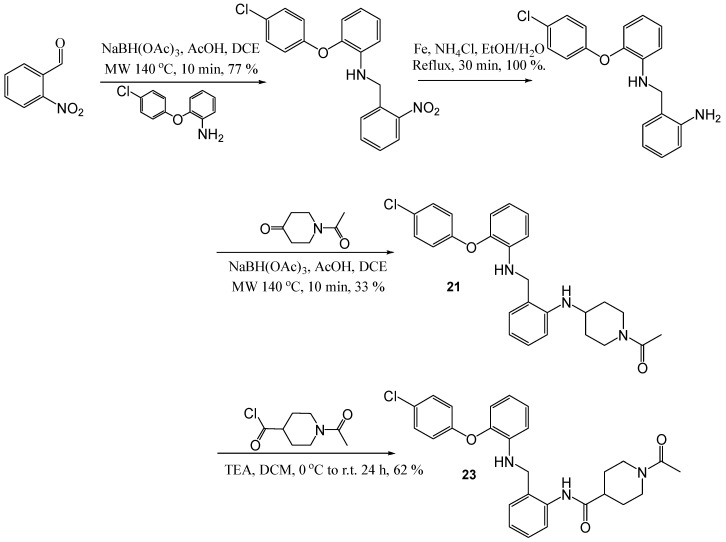
Synthesis of extended 2-acetyl analogues **21** and **23**.

It was then decided to investigate whether this *N*-acetyl protection was required, or whether the steric constraints and decreased reactivity of the aniline NH were enough to stop potential side reactions from occurring. Therefore, the route was modified (Scheme 12). The reductive amination was carried out with 1-acetyl-4-piperidone. The microwave-assisted reaction occurred successfully, albeit low yielding, to produce the desired **21**. Previously, extended compounds in the original amine and amide linked series had been extended using an amide bond. The same primary amine intermediate was, therefore, reacted with 1-acetyl-piperidine-4-carbonyl chloride to produce **23**, extended via an amide bond (Scheme 12).

**Scheme 13:**
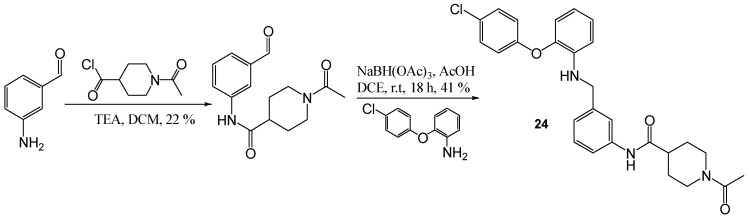
Synthesis of the extended 3-acetyl-*N*-piperidinyl analogue, compound **24**.

It was also decided to synthesise an extended compound with the *N*-acetamide group migrated around the ring to the 3 position (Scheme 13). This compound could be synthesised using 1-acetyl-piperidine-4-carbonyl chloride as the acid chloride to give **24**, after reductive amination.

The extended analogues were evaluated biologically as before. The results are shown in Table 4 and show interesting patterns. The extension of this series via an amine bond (**21**) is detrimental to activity, reducing the percentage inhibition at 10 µM to 36% and, for the *N*-acetyl analogue **22**, to 55%. Extension of this series via an amide bond (**23** and **24**) was much more promising, with percentage inhibitions of 91 and 97%, respectively. The IC_50_ values for these two compounds show a slight reduction in activity compared to compound **1**, (IC_50_ = 0.9 μM) with IC_50_ values of 3.9 and 1.1 μM, respectively (Table 4).

**Figure 7:**
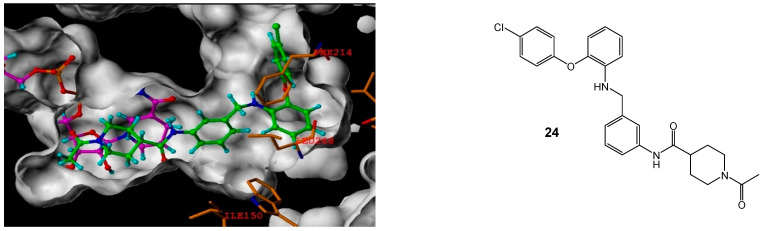

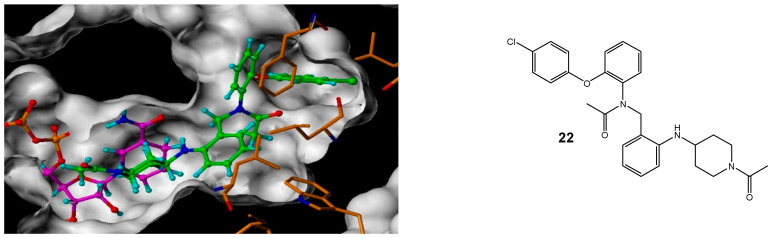
Compounds **24** and **22** docked into the 17β-HSD3 homology model.

The extended compounds were analysed in silico, and the docking of compounds **22** and **24** is shown in Figure 7. These show that both these compounds are aligned very differently to the shorter compounds without the extra pyridine ring, such as **18**. 

In both cases, the extra length of the compounds means that the hydrophobic head group lies within the hydrophobic pocket. From these docking figures alone, it is hard to see why there is such a difference in activity between **22** and **24**. Both have a similar alignment and are similar in length. It can be hypothesized that the difference is due to a favourable interaction between the carbonyl of the amide (X) in **24**, whereas this is absent in **22**. This slight increase in length may also mean that the carbonyl of the *N*-acetamide group in **22** can form a better interaction with the cofactor at the base of the active site. The 3-substituted compound **24** is more linear than the corresponding 2-substituted compound **23**; this difference in linearity may explain the difference in activity between them, as the active site appears to have a narrower region in the centre. 

Therefore, the more linear compounds may fit better within this channel unaffected by unfavourable steric constraints. Extension of the compounds may also have the added advantage that if the compounds are a more restricted fit, they may occupy the active site more tightly, improving potency and selectivity. 

### 2.8. Modifications to the Benzylamine Template by Substitution onto the CH_2_ of the Benzylamine Linker

**Figure 8:**
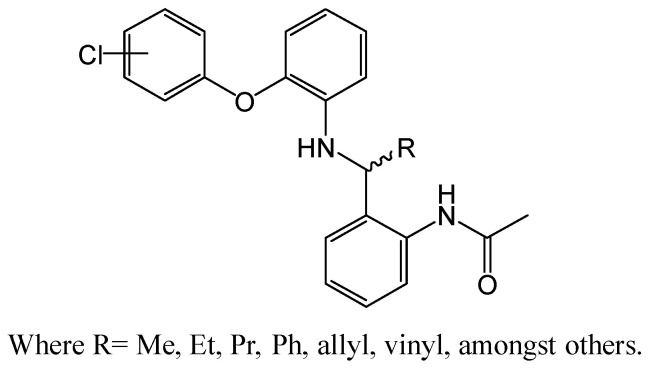
Novel *C*-substituted benzylamine linkers.

A favourable feature of SCH-451659 (Figure 2) is the (*S*)-lipophilic *t*-butyl group on the piperidine ring. It was demonstrated that this region leads to a very favourable increase in inhibitory activity [13]. It is, therefore, predicted to be beneficial to incorporate a hydrophobic group that can mimic this region. Within this series of benzylamine targets, there are a few regions that may be able to mimic this group, including the central aromatic ring and substitution around this ring. It is also possible that inclusion of alkyl groups onto the methylene of the amine link may have favourable hydrophobic interactions. Therefore, a range of targets with substitutions in this region was designed (Figure 8).

### 2.9. Reductive Amination Reactions of 2-Amino-Acetophenone

The introduction of a simple methyl substituent was the initial target (R = Me from Figure 8). The synthesis was started from the commercially available 2-amino-acetophenone, which was acetylated and then subjected to a reductive amination with the hydrophobic head group as before. We had previously shown [22] that microwave-assisted reductive amination is fast and efficient. However, in this case, the standard reductive amination conditions reactions were unsuccessful (Table 5) [22], even with an extended reaction time (Entry 1). The same lack of reactivity was observed when traditional Dean–Stark conditions were used (Entry 2). 

Many different sets of conditions for an indirect reductive amination were attempted (Entries 3–6). TiCl(O^i^Pr)_3_ has been used under several conditions for this reaction. Using TiCl(O^i^Pr)_3_ as the Lewis acid, it was attempted to form the imine, which could then be reduced in situ. Unfortunately, none of the sets of conditions attempted led to the desired product. 

**Scheme 14:**
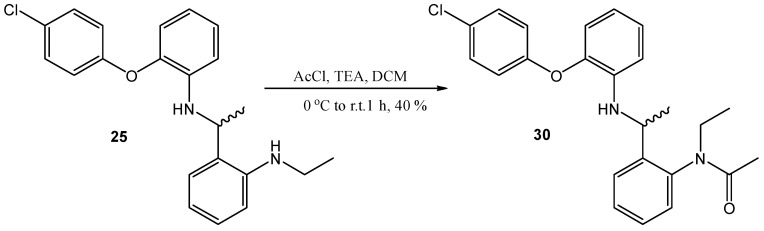
Synthesis of **30** from **25**.

In some cases, however, an interesting phenomenon was observed. The use of sodium borohydride as the reducing agent (Entry 3) led to the formation of an alternative product **25**, (Scheme 14) where the two starting materials have undergone a reductive amination as intended. However, the *N*-acetyl amide bond was also reduced to an ethyl group. Attempts to control this reaction, by using different reagents or temperatures, were not successful, as in some cases only starting materials were isolated and others produced the reduced amide as before (**25**, Entries 4–6). 

The synthesis of **25** may, however, be beneficial, as it will help to investigate the effects of the amide bond upon enzyme binding. The synthesis of **25** was then acetylated to produce **30** (Scheme 14), where the *N*-acetyl group is present, but the NH of the amide is substituted with an ethyl group. 

It had not been possible to determine whether the problem with this reaction lay with the formation or reduction of the imine. It was, therefore, attempted to form and identify, or, if possible, even isolate the intermediate imine. However, this was unsuccessful and showed that the problem lay with the formation of the imine, probably due to the low reactivity of the ketone, meaning that a new synthetic route was required. 

Although the synthetic route did not achieve the desired efficient synthetic route, compound **30** has the highest biological activity of the compounds reported in this paper with an IC_50_ of 74 nM (Table 9).

### 2.10. Use of Organometallic Reagents

**Scheme 15:**
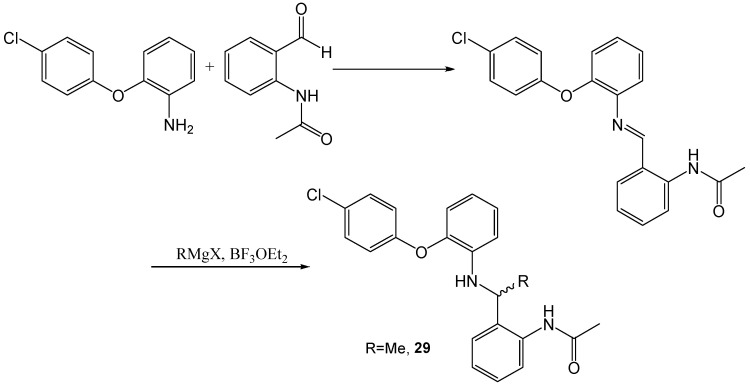
Second proposed synthetic route to substituted targets.

It was previously shown (Scheme 1) that the diphenyl ether aniline reacts successfully with the aldehyde, *N*-(2-formyl-phenyl)-acetamide, in a reductive amination to form compound **1**. It was hypothesised that if the intermediate imine could be formed, then organometallic chemistry techniques could be used to insert the desired alkyl groups at the imine bond, thus forming the desired products. The proposed synthetic route is shown in Scheme 15. 

The imine is formed and isolated, before being reacted with a Grignard reagent to add the desired alkyl group. This route is ideal for this series of targets, as the intermediates remain common until the last step, allowing for efficient diversification.

The formation of the imine intermediate could be achieved using two different sets of conditions: either by stirring in DCM with 10 eq. of anhydrous MgSO_4_ for 18 h, or by stirring in DCM with 2 eq. TiCl(O^i^Pr)_3_ for 4 h [29]. Both sets of conditions were effective, but the second procedure was chosen as it showed better reproducibility and exhibited a shorter reaction time. The conversion of the aldehyde to the imine was easily detected by NMR spectroscopy, as there was a distinct difference in the chemical shift between the aldehyde proton at 9.90 ppm in the starting material and the imine proton at 8.60 ppm.

Once the imine had been formed, the Grignard reactions were carried out immediately, thus minimising any chance of imine degradation. BF_3_OEt_2_ has been shown to enhance the reaction, so this was used in the procedures. Many different Grignard reagents were tested (Table 6). However, the majority of these met with little success. 

The procedures where phenyl, isopropyl, cyclopropyl, vinyl or methyl magnesium bromide were used (Entries 1–5) all exhibited a lack of reactivity, as in all cases a significant proportion of the starting imine was isolated. It was attempted to overcome this lack of reactivity using both microwave conditions (Entry 6) and conventional reflux conditions (Entry 7) [30]. In both cases, this caused decomposition of the imine without producing any of the desired product, the aniline being the only compound isolated.

Some success was observed, as the reactions with both allyl magnesium bromide and benzyl magnesium bromide were moderately successful (Entries 8 and 9). These were carried out under the same conditions as previously with BF_3_OEt_2_ added, and, as previously, the reaction mixtures were stirred for 18 h at room temperature. The allyl-substituted product was isolated in 57% yield (**26**), and the benzyl-substituted product in 8% yield (**27**).

**Scheme 16:**
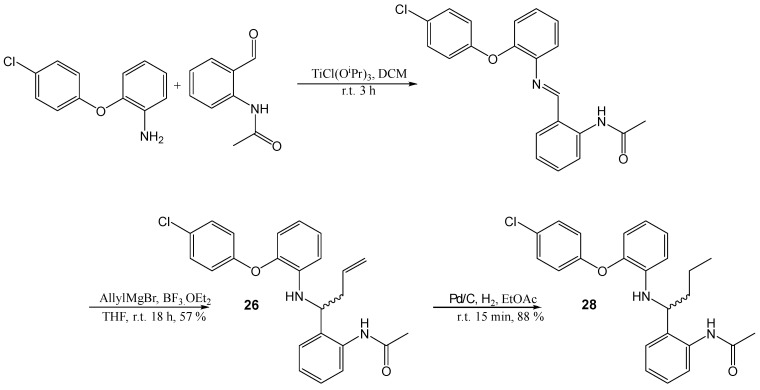
Synthesis of **26** and its reduction to form **28**.

The distinct difference between the reactivity of the allyl and benzyl Grignard reagents in contrast to the other Grignard reagents used may be due to the reagent basicity. Organometallic reagents are strong bases, so they might deprotonate the imine (pKa ~22–24) as well as the amide NH [31,32]. This would inactivate the Grignard, forming the corresponding alkane, though this can be compensated for by the usage of an excess of reagent (e.g., three equivalents here). However, imine deprotonation means it is no longer open to attack by the nucleophilic Grignard reagent. The imine stays in this deprotonated state until subjected to aqueous work-up, where it is re-protonated, regenerated and, in some cases, isolated. The allyl and benzyl Grignard reagents are much weaker bases, so are less likely to deprotonate the imine, which is available to react with the nucleophilic Grignard reagent. Only the allyl Grignard reaction worked in good yield (57%), with the benzyl group being introduced in 8% yield. This may be due to imine decomposition or to some deprotonation still occurring, especially in the case of the benzyl Grignard. The allyl-substituted product (**26**) is a useful intermediate and could be successfully reduced to produce the propyl substituted compound (**28**, Scheme 16). This was achieved using palladium on carbon as the catalyst, in an atmosphere of hydrogen at atmospheric pressure. It was a fast reaction, reaching completion in just 15 min to produce compound **28** in 88% yield.

At this stage there were three compounds in this series, the allyl- (**26**), benzyl- (**27**) and propyl-substituted (**28**) compounds. However, a general route to a wider series of compounds had not been identified, so further studies using organometallic addition reactions were investigated (Table 7).

Some possibly useful alternatives were identified [33,34,35,36,37]. Unfortunately, the use of zinc chloride (Entry 1), copper iodide (Entry 2), scandium triflate (Entry 3) and the *N*-heterocyclic carbene, 1,3-bis(2,4,6-trimethylphenyl)-imidazolinium chloride (Entry 4), were unsuccessful; in most cases, the imine was isolated. 

Attempts were made to alter the reactivity of the reaction in some way. For example, the use of copper iodide means that the copper would trans-metallate the Grignard to give an organocopper reagent, which is softer in nature than the Grignard reagent. This aimed to match the reactivity of the reagent to the imine bond; however, it was not successful. When cerium chloride was used as an additive, success was obtained (Entry 5) and the desired methyl substituted product was synthesised (**29**), as shown in Scheme 15. It was hypothesised that the cerium chloride increased the reactivity of the Grignard by decreasing basicity and improving nucleophilicity. This further helps to explain that the problem with this reaction, as mentioned previously, may be due to the Grignard reagent acting as a base, deprotonating the imine and, therefore, stopping the desired reaction. However, the yield was extremely low, with just 8% of product isolated. The reaction was found to be unreliable and this method was not pursued further. 

An alternative option to a Grignard reagent is to use an alkyl lithium reagent. The attempted procedures are shown in Table 8. However, similar problems with reactivity were encountered with the use of methyl lithium as with the methyl Grignard, so only the starting imine could be isolated. 

A review of the chemical literature revealed that dimethyl copper lithium could be used as an alternative [39]. This was made in situ using methyl lithium and copper iodide. However, this was also unsuccessful. 

Ultimately, a suitable method to synthesise a library of diverse compounds in this series was not found. However, the method described in Table 7 (Entry 5) did enable sufficient synthesis of compound **29** (Scheme 15) to enable biological evaluation, and this pleasingly showed an IC_50_ of 76 nM. Although very potent, due to the difficulty of synthesis this compound was not pursued and the *C*-allyl analogue of similar potency (**26**) was chosen for further studies (see Table 9 for biological data), as this was easily synthesised on a large enough scale for the chiral separation of the pure enantiomers.

### 2.11. Investigations into the Effects of Chirality on Enzyme Inhibition

While many of the compounds prepared in this benzylamine series are achiral, thus simplifying SAR development, the enantiopure forms of a racemic compound need to be subjected to biological evaluation separately to establish if one or both enantiomers are active. 

The most successful synthesis in this series so far was that of **26**, the allyl-substituted compound (Scheme 16) that was prepared in 57% yield. This allowed a suitable amount of the racemate (*ca*. 200 mg) to be prepared. Preparative chiral HPLC was used for separation of the pure enantiomers. Compound **26** was subjected to separation using an analytical scale Chiralcel AD-H chiral HPLC column, by eluting with 80% methanol and 20% water at 1.0 mL/min; the chromatograph showed that the mixture contained a 1:1 mixture of enantiomers and separation of the enantiomers was possible with no baseline overlap. Enantiomer A (**31**) had a retention time (T_R_) of 9.0 min and enantiomer B (**32**) a T_R_ of 11.5 min. Separation on a preparative scale using the same column specification as above allowed ~70 mg of each compound to be isolated and analysed [see Supplementary Materials].

### 2.12. Single Crystal X-ray Crystallography

**Figure 9:**
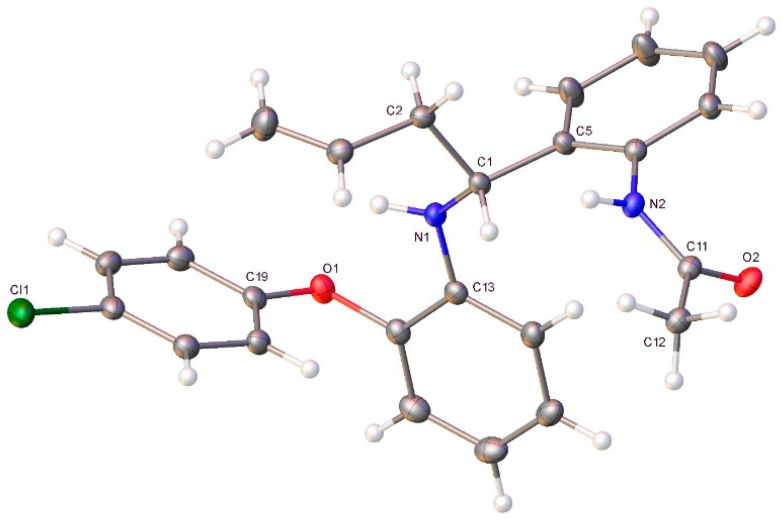
X-ray crystal structure of enantiomer A (**31**, (*R*)-). Inactive. Ellipsoids are represented at 30% probability.

**Figure 10:**
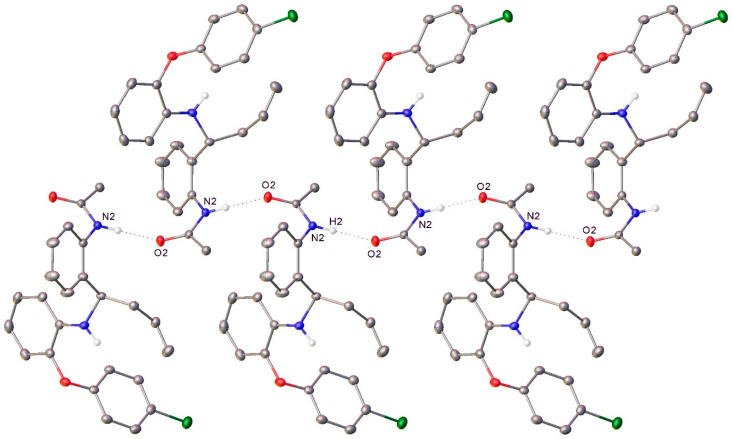
The packing structure of Enantiomer A (**31,** (*R*)-).

To identify the absolute stereochemistry of the enantiomers, it was necessary to employ X-ray crystallography. Both enantiomers were crystallised (from hexane/DCM) and crystallographic analysis of enantiomer A (**31**, see Experimental Section, Figure 9 and Figure 10, see also Supplementary Materials) permitted its conclusive identification as the *R*-enantiomer, meaning that B (**32**) is, therefore, the *S*-enantiomer.

The crystal form is orthorhombic, with space group *P*2_1_2_1_2_1_ and a unit cell that contains four molecules. H1A and H2 (attached to N1 and N2, respectively) were in the crystal structure and refined at a distance of 0.9 Å from the relevant parent nitrogen atom. The gross structure is dominated by 1-dimensional, hydrogen-bonded polymers, which propagate along the *a*-axis by virtue of intermolecular interactions between H_2_ (attached to the acetamide nitrogen N_2_) and the carbonyl oxygen, O_2_.

**Figure 11:**
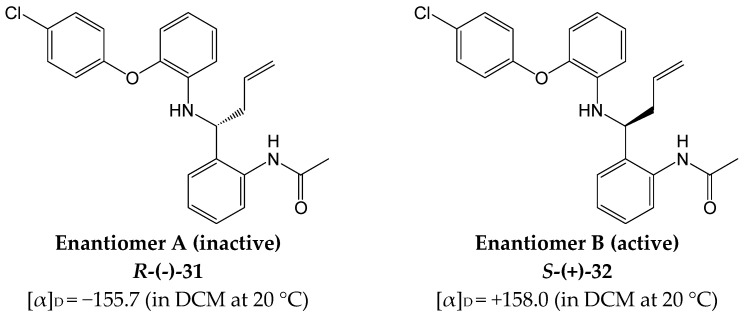
Enantiomers separated from compound **26**.

The optical rotation of **31** and **32** was measured, and the results and structures are shown in Figure 11. The optical rotations (measured as [α]_D_) were directly opposite for the two compounds and of a similar magnitude, therefore enantiomer A is *R*-(-)- and B is *S*-(+)-. 

The racemic mixture (**26**) showed inhibition values of between 20–60% when tested at 10 µM in the 17βHSD3 inhibition assay; the (*S)*-enantiomer (B, **31**) showed >60% inhibition at 10 µM and an IC_50_ = 370 nM, while the (*R*)-enantiomer (A, **32**) was inactive.

**Figure 12:**
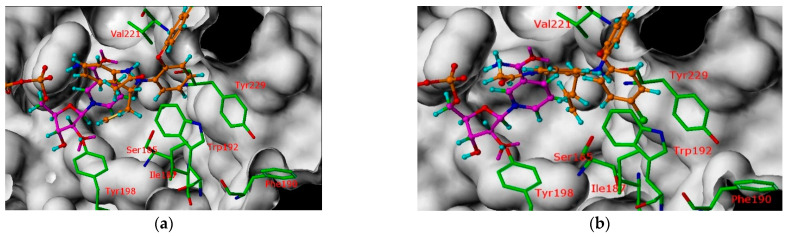
(**a**) The inactive enantiomer A (**31**, *R*-(-)-) and (**b**) the active enantiomer B (**32**, *S*-(+)-) docked into the 17βHSD3 homology model.

Both enantiomers were docked into the 17β-HSD3 homology model as shown in Figure 12. Both compounds docked in multiple modes due to the high degree of flexibility from the high number of rotatable bonds (only one mode is shown in Figure 12). In the (*S)*-enantiomer B (**32**), a hydrogen bond is predicted between the diphenyl-ether oxygen and a nitrogen atom in Val221 side chain; the hydrophobic pocket in the model indicates a predicted tight binding with the hydrophobic groups of the inhibitor, indicating this to be the most potent enantiomer as evidenced by the biological results and proves the importance of the directionality of the allyl group on binding in the active site.

### 2.13. Summary of Biological Activity

The inhibition of 17β-HSD3 by the novel compounds in our substituted aryl benzylamine series are listed in summary in Table 9. IC_50_ values were calculated for compounds showing >70% inhibition at 10 μM, and compounds showing <20% inhibition at 10 μM are classed as inactive (IA).

### 2.14. Selectivity over 17β-HSD Types 1 and 2, Androgenicity and Cytotoxicity

Inhibitors listed in Table 9 are also essentially selective over the type 2 isozyme of 17β-HSD2 and show <20% inhibition when tested at 10 µM. Compounds were also assessed for activity against the type 1 isozyme and for any androgenic or cytotoxic activity. Illustrative details for some of the more active compounds are provided below. Table 10 shows the inhibitory activity data obtained when selected 17β-HSD Type 3 inhibitors were tested in the 17β-HSD Type 1 assay. A selection of those compounds that exhibited significant 17β-HSD Type 3 inhibition were submitted for testing. The results show that such compounds are essentially inactive against 17β-HSD Type 1. The activity of a selection of 17β-HSD Type 3 inhibitors on 17β-HSD Type 2 was also assessed (Table 10). Compounds are also only weakly active against 17β-HSD Type 2. Generally, compounds are no less selective than SCH-451659 as a positive control or against the similar benchmark STX2171. 

Analogues were also assessed for potential androgenic and cytotoxic effects as well as against benchmarks. No androgenicity or significant cytotoxicity was found (Table 11).

## 3. Conclusions

We reported earlier on a small series of six- and seven-membered cyclic 17β-HSD3 inhibitors, using SCH-451659 as lead, as potential anti-cancer agents [17] via application of structure-based drug design encompassing pharmacophore generation, database mining, homology model construction and inhibitor docking. Excellent nanomolar level activity was observed, with STX2171 also showing good activity in vivo [21]. 

The design and synthesis of a related novel *N*-aryl benzylamine series of selective 17β-HSD3 inhibitors is reported here, using our homology model with docking studies to identify and optimise candidate compounds, with insights on how inhibitors interact with the active site. Some inhibitors of this new series exceeded the potency of STX2171, with the best *N*-(2-(1-[2-(4-chloro-phenoxy)-phenylamino]-ethyl)-phenyl)-acetamide **30** having an IC_50_ of 74 nM. Optimisation also gave potent compounds that are selective over the type 2 enzyme. 

Hydrogen bonding with amino acid residues Tyr229, Ser185 and Trp192 of 17β-HSD are important to *H*-bond to suitable inhibitor groups, such as an acetyl group on the aryl group and especially for those with extended linkers, where improved activity was observed. *N*-Acetyl-linked compounds showed loss of activity, indicating the importance of conformation on activity. Racemic mixtures of *C*-substituted methyl-, ethyl- and allyl-linked analogues prepared by organometallic routes have good activity, enhancing our theory of tight binding in the hydrophobic region. The enantiomers of racemate **26** were separated using chiral chromatography, and absolute configuration was determined using single crystal X-ray crystallography. Only the *S*-(+)- enantiomer **32** was active, which was predictable through docking studies, adding further confidence to our model. 

In summary, a general reductive amination procedure was used to synthesise the novel benzylamine-based series presented here, with sodium tri-acetoxy borohydride being the favoured reagent. Both diverse classical and new chemistries were employed in this work, such as heterogeneous and homogeneous oxidations; organometallic conjugate addition to imines using both a Grignard reagent approach; and newer alternatives. Use of chiral chromatography and X-ray crystallography was exemplified to establish enantiomeric absolute configuration for biological comparison in one case. The *N*-aryl benzylamine series reported here shows good, selective activity against a still under-developed target for hormone-dependent prostate cancer and is, therefore, worthy of further optimisation and development for further evolution of 17β-HSD3 inhibition.

## 4. Experimental Section

### 4.1. Materials and Methods

All chemicals were purchased from Aldrich Chemical Co. (Gillingham, UK) or Lancaster Synthesis (Morecambe, UK). All organic solvents were supplied by Fisher Scientific (Loughborough, UK). Reactions using anhydrous solvents were carried out under nitrogen. Thin layer chromatography (TLC) was performed on precoated plates (Merck TLC aluminium sheets silica gel 60 F254). Product(s) and starting material(s) were detected by either viewing under UV light and/or treating with a suitable staining system followed by heating. Flash column chromatography was performed on silica gel (Sorbsil/Matrex C60) or using Argonaut prepacked columns with a Flashmaster TM. IR spectra were recorded in a DCM solution on PerkinElmer Spectrum RXI FT-IR spectrometer cells and peak positions are expressed in cm^−1^. ^1^H NMR (270 MHz or 400 MHz) and DEPT-edited ^13^C NMR (68 MHz or 101 MHz) spectra were recorded with a Jeol Delta 270, or a Varian Mercury VX 400 NMR spectrometer, and chemical shifts are reported in parts per million (ppm). HPLC analyses were performed on a Waters Millenium 32 instrument equipped with a Waters 996 PDA detector. A Waters C18 Radial-Pak-reversed phase column (8 × 100 mm) was eluted with the solvent system specified at 1 mL/min. Microwave irradiation was carried out using a CEM Discover^®^ instrument (CEM Microwave Discovery Ltd., Buckingham, UK). FAB^−^, low- and high-resolution mass spectra were recorded at the Mass Spectrometry Service Centre, University of Bath, using m-nitro benzyl alcohol (NBA) as the matrix. ES and APCI low-resolution mass spectra were obtained on a Waters Micromass ZQ. Elemental analyses were performed by the Microanalysis Service, University of Bath.

### 4.2. Biological Assays

#### 4.2.1. Assay for 17β-HSD3 Activity

Human 293-EBNA cells stably transfected with 17β-HSD3 were plated at 50,000 cells/well in 24 well plates in complete growth medium. After 48 h 2–3 nM [^3^H]-androstenedione in assay medium (500 mL DMEM medium with 5 mL 100 × L-glutamine and 5 mL 7.5% sodium bicarbonate solution) was added with or without test compound at 1.5 mL/well (triplicate), and the cells incubated at 37 °C. Two hours later 1 mL medium was removed from each well and placed in a 125 × 16 mm glass test tube containing 25 µL of recovery solution (5000 dpm ^14^C-testosterone and 25 µg unlabelled testosterone ether (4 mL) was added, and the tubes vortexed at high speed for 2 × 30 s. After the samples had settled into two phases, they were snap-frozen in a dry ice/methanol bath. The upper organic phase was decanted into 75 × 12 mm tubes and evaporated to dryness under an airstream using a sample concentrator (TECHNE) at 40 °C. The samples were resuspended in ether (8 drops, then a further 3), spotted onto ca 60 F254 20 cm × 20 cm TLC plates and separated using a 4:1 *v*/*v* dichloromethane: ethyl acetate mobile phase. After drying the plates, the major spots were marked under a UV lamp, cut out and placed in individual scintillation vials containing 0.5 mL methanol. These were then shaken lightly and allowed to stand for 15 min before adding 10 mL of EcoScintA (scintillation fluid) to each tube along with 0.5 mL assay medium and counted in a scintillation spectrometer (Beckman, High Wycombe, Bucks, UK), using a program for duel [^3^H/^14^C] isotopes. The number of cells/well was then counted using a Coulter10 counter (Beckman). The inhibitory activity of the test compounds was then assessed by calculating the amount of product formed correcting for crossover between isotope counts, recovery, dilution and non-enzymatic degradation fmol/hr/million cells) with and without inhibitor (% inhibition). 

#### 4.2.2. 17β-HSD1 Activity Assay

The assay is of a similar format to the 17β-HSD Type 3 TLC assay. T-47D breast cancer cells, which express a high ratio of 17β-HSD Type 1 to 17β-HSD Type 2, were used to test the conversion of labelled estrone to estradiol in the presence and absence of the potential inhibitors. The cells were incubated with [^3^H]-estrone at a concentration of 2 nM, in the absence or presence of the inhibitors (10 μM). After incubation of the culture for 3 h at 37 °C, the products were isolated and separated by TLC using DCM/ EtOAc (4:1). The amount of estradiol formed was measured and compared to the control cells, where no inhibitor was present [40]. Each assay was carried out in the presence of a positive control (IC_50_ of 27 nM) [41] to ensure reproducible results were obtained.

#### 4.2.3. 17β-HSD2 Activity Assay

MDA-MB-231 human breast cancer cells in T25 flasks were incubated with 2 nM [^3^H]-E2 (41.3 Ci/mmol; PerkinElmer, Beaconsfield, Bucks, UK) in the presence or absence of 10 µM candidate 17β-HSD3 inhibitors. After 3 h at 37 °C, the steroids were isolated from the mixture by extraction with diethyl ether and procedural losses were measured using [^14^C]-E1 (5000 dpm; PerkinElmer). Separation of [^3^H]-E1 from [^3^H]-E2 was achieved using thin layer chromatography, eluting with dichloromethane/ethyl acetate, 4:1 *v*/*v* and the amount of [^3^H]-E1 produced was calculated from the [^3^H]-E1 produced, which was calculated from the [^3^H] counts detected and recovery of [^14^C]-E1 (Beckman LS 6000 SC). A positive control was used in this assay (IC_50_ 5.4 μM) to ensure reproducible results were obtained [42].

#### 4.2.4. Compound Toxicity

AR+ (LNCaP) and AR- (PC-3) PCa cell lines were used as standard lines to determine the cytotoxicity of HSD3 inhibitor compounds. Toxicity was assessed over 96 h in 96 well plates using the standard MTS/Alamar Blue proliferation assay and over longer durations by cell counting methodologies.

#### 4.2.5. X-ray Crystallography

Single crystals of **31** (enantiomer A) were selected and a suitable crystal was mounted on a diffractometer. The crystal was kept at 150.15 K during data collection., using Olex2 [43]. The structure was solved with the SHELXS-1997 method [44] structure solution program using Direct Methods and refined with the SHELXL [45] refinement package using Least Squares minimisation. Crystal Data for C_24_H_23_ClN_2_O_2_ (*A_r_* = 406.89): orthorhombic, space group *P*2_1_2_1_2_1_ (no. 19), *a* = 8.9500(1) Å, *b* = 10.4450(1) Å, *c* = 22.6800(2) Å, *U* = 2120.19(4) Å^3^, *Z* = 4, *T* = 150 K, *μ*(MoK_α_) = 0.202 mm^−1^, *D*_calc_ = 1.275 g cm^−3^, 42,345 reflections measured (7.056° ≤ 2θ ≤ 60.074°) and 6200 unique (*R*_int_ = 0.0403), which were used in all calculations. The final *R*_1_ was 0.0329 (*I* ≥ 2σ(*I*)) and *wR*_2_ was 0.0796 (all data). 

### 4.3. Synthesis of Benzylamine-Linked 17β-HSD3 Inhibitors

#### 4.3.1. General Procedure for the Reduction of Substituted 2-Nitrobenzaldehyde

A solution of the desired substituted 2-nitrobenzaldehyde in EtOH (5 mL/mmol) was cooled to 0 °C and to this NaBH_4_ (1.5 eq.) was added; the resulting solution was stirred at r.t. for 2 h. The EtOH was removed in vacuo and a saturated solution of NH_4_Cl was added before the mixture was then extracted with DCM and dried (MgSO_4_). It was then evaporated in vacuo to yield the desired substituted 2-nitrobenzyalcohol.

#### 4.3.2. General Procedure for the Reduction of the Substituted 2-Nitrobenzylalcohol

This procedure is as described by Matsuo et al. To a refluxing mixture of iron (5.5 eq.) and ammonium chloride (0.7 eq.) in a 10:1 mixture of EtOH:H_2_O, the substituted 2-nitrobenzylalcohol (1 eq.), was added. This reaction mixture was stirred at reflux for between 1 and 4 h, and followed by TLC; it was then allowed to cool to room temperature and the solvent was removed in vacuo. The residue was redissolved in DCM and washed with sat. aqueous sodium bicarbonate. The organic layer was dried (MgSO_4_), filtered and evaporated in vacuo to afford the desired 2-aminobenzyalcohol. 

#### 4.3.3. General Procedure for the Acylation of Substituted 2-Aminobenzylalcohols

To a solution of the substituted 2-aminobenzylalcohol and TEA (3 eq) in DCM (10 mL/1 mmol) at 0 °C, acetyl chloride (6 eq.) was added. The resulting solution was allowed to warm to r.t. and stirred for 18 h. NaHCO_3_ was added and the crude mixture was repeatedly extracted with DCM (with a trace of MeOH). The organic layers were then washed with 1 M HCl. The organic layers were combined and dried (MgSO_4_), filtered and evaporated in vacuo. The resulting solid was redissolved in MeOH (40 mL/1 mmol), NaOH (3 eq.) was added and the reaction was stirred at r.t. for 2 h. The solvent was evaporated in vacuo and H_2_O was added; the crude mixture was repeatedly extracted with EtOAc. The organic layers were combined and dried (MgSO_4_), filtered and evaporated in vacuo to obtain the desired 2-acetamide benzyl alcohol.

#### 4.3.4. General Procedure for the Dess-Martin Periodinane Oxidation of Alcohols

This procedure is based upon work by Dess et al. To a solution of the 2-acetamide alcohol in DCM (50 mL/1 mmol) Dess-Marin Periodinane (1.5 eq.) was added; the resulting solution was stirred at r.t. for 10 min. Sodium thiosulphate (4.5 eq.) in NaHCO_3_ was then added, and the mixture was repeatedly extracted with DCM. The organic layers were combined and dried (MgSO_4_), filtered and evaporated in vacuo. The crude mixture was purified using flash chromatography to afford the desired aldehyde.

#### 4.3.5. General Procedure for the Reductive Amination of the Substituted Diphenyl Ether Aniline with the Substituted 2-Acetamide Benzaldehyde

To a solution of the diphenyl ether aniline (1.5 eq.) and aldehyde (1 eq.) in DCE (2 mL/1 mmol), acetic acid (3 eq.) and sodium tri-acetoxyborohydride (2.5 eq.) were added. The resulting reaction mixture was stirred at r.t. for 2–18 h. NaHCO_3_ was then added and repeatedly extracted with DCM. The organic layers were combined and dried (MgSO_4_), filtered and evaporated in vacuo. The crude mixture was purified using flash chromatography to afford the desired compound.

##### 2-Amino-benzaldehyde

Using the general procedure for the reduction of the 2-nitrobenzylalcohol, the desired compound was isolated as a yellow oil, 794 mg, 99% yield. R.f. 0.35 (DCM), LCMS: *t*_r_ = 3.04 min (50% to 95% MeOH in water at 0.5 mL/min to 1.0 mL/min over 5 min), m/z M + H 122.15, ^1^H NMR (CDCl_3_, 270 MHz,): δ 6.12 (2H, s, NH_2_), 6.63 (1H, d, *J* = 8.4 HZ, ArH), 6.70–6.76 (1H, m, ArH), 7.26–7.32 (1H, m, ArH), 7.46 (1H, dd, *J* = 1.5, 7.7 Hz, ArH), 9.85 (1H, s, CHO). 

##### *N*-(2-Formyl-phenyl)-acetamide

Using the general procedure for the acylation of substituted 2-aminobenzylalcohols, the title compound was obtained as a yellow solid, 190 mg, 71% yield. R.f. 0.68 (DCM), m.p. 54–57 °C, LCMS: *t*_r_ = 1.67 min (90% MeOH in water), m/z M + H 164.1, ^1^H NMR (CDCl_3_, 270 MHz): δ 2.24 (3H, s, CH_3_), 7.21 (1H, td, *J* = 1.2, 7.7 Hz, ArH), 7.56–7.63 (1H, m, ArH), 7.65 (1H, dd, *J* = 1.5, 7.7 Hz, ArH), 8.72 (1H, d, *J* = 8.4 Hz, ArH), 9.85 (1H, s, CHO), 11.12 (1H, s, NH). ^13^C NMR (CDCl_3_, 68 MHz): δ 25.6 (CH_3_), 119.9 (ArCH), 121.5 (ArC), 123.0, 136.2, 136.4 (ArCH), 141.0 (ArC), 169.8 (CO), 195.7 (CHO). HRMS: Calcd. for C_9_H_9_NO_2_ (M + H)^+^ 164.0706, found (M + H)^+^ 164.0699.

##### *N*-(2-([2-(4-Chloro-phenoxy)-phenylamino]-methyl)-phenyl)-acetamide (**1**)

To a solution of 2-(4-chloro-phenoxy)-phenylamine (0.128 g, 0.58 mmol) and N-(2-formyl-phenyl)-acetamide **12** (0.19 g, 1.16 mmol) in DCE (2.6 mL), acetic acid (0.25 mL) and sodium tri-acetoxy borohydride (0.31 g, 1.45 mmol) were added. The resulting reaction mixture was heated in a CEM microwave for 10 min at 140 °C. NaHCO_3_ was added and the mixture was repeatedly extracted with EtOAc. The organic layers were combined and dried (MgSO_4_), filtered and evaporated in vacuo. The crude mixture was purified using flash chromatography (0–100% DCM in hexane) to afford the title compound as a white solid, 80 mg, 38% yield. R.f. 0.33 (DCM), m.p. 194–196 °C, LCMS: *t*_r_ = 1.36 min (95% MeOH in H_2_O), m/z M - H 365.4, HPLC: *t*_r_ = 5.1 min (90% acetonitrile in H_2_O, 0.5 mL/min), 98%, ^1^H NMR (DMSO, 400 MHz): δ 2.02 (3H, s, CH_3_), 4.27 (2H, d, *J* = 5.6 Hz, CH_2_), 5.95 (1H, s, NH), 6.46 (1H, d, *J* = 8.4 Hz, ArH), 6.55 (1H, td, *J* = 0.8, 7.6 Hz, ArH), 6.84 (1H, dd, *J* = 1.6, 8.0, ArH), 6.89–6.95 (3H, m, ArH), 7.07–7.11 (1H, m, ArH), 7.16–7.22 (2H, m, ArH), 7.35–7.41 (3H, m, ArH), 9.47 (1H, br.s, NHCO). ^13^C NMR (DMSO, 101 MHz): 23.2 (CH_3_), 42.6 (CH_2_), 111.7, 116.0, 118.3, 120.2, 125.2, 125.3, 125.6 (ArCH), 126.0 (ArC), 126.7, 126.9, 129.6 (ArCH), 133.5, 135.8, 140.5, 141.6, 156.7 (ArC), 168.5 (CO). HRMS: Calcd. for C_21_H_19_ClN_2_O_2_ (M + H)^+^ 367.1208, found (M + H)^+^ 367.1204. Anal. Calcd. for C_21_H_19_ClN_2_O_2_: C 68.76, H 5.22, N 7.64%. Found: C 69.0, H 5.28, N 7.52%.

##### *N*-(2-([2-(4-Trifluoromethoxy-phenoxy)-phenylamino]-methyl)-phenyl)-acetamide (**2**)

Using the general procedure for the reductive amination of the substituted diphenyl ether aniline with the substituted 2-acetamide benzaldehyde, the desired compound was isolated as a white solid, 155 mg, 55% yield. R.f. 0.4 (1:1, EtOAc: Hexane), m.p. 98–100 °C, LCMS: *t*_r_ = 1.07 min (95% MeOH in H_2_O), m/z M - H 415.09, HPLC: *t*_r_ = 2.22 min (90% acetonitrile in H_2_O), 99%, ^1^H NMR (CDCl_3_, 270 MHz): δ 1.94 (3H, s, CH_3_), 4.3 (3H, s, CH_2_ and NH), 6.79–6.96 (5H, m, ArH), 7.06–7.17 (3H, m, ArH), 7.25–7.34 (3H, m, ArH), 8.01 (1H, d, *J* = 8.15, ArH), 8.54 (1H, br.s, NHCO). ^13^C NMR (CDCl_3_, 68 MHz): δ 24.5 (CH_3_), 47.8 (CH_2_), 113.8, 118.1, 119.6, 119.7 (ArCH), 122.5 (ArC), 122.8, 124.6, 125.7 (ArCH), 127.6 (ArC), 128.9, 129.6 (ArCH), 137.5, 139.9, 143.9, 144.4 (ArC), 155.9 (OCF_3_), 168.5 (CO). ^19^F NMR (CDCl_3_, 376 MHz): δ - 58.29 (OCF_3_). HRMS: Calcd. for C_22_H_19_F_3_N_2_O_3_ (M + Na)^+^ 439.1240, found (M + Na)^+^ 439.1240. Anal. Calcd. for C_22_H_19_F_3_N_2_O_3_: C 63.46, H 4.60 N 6.73%. Found: C 63.5, H 4.62, N 7.0%.

##### *N*-(2-([2-(4-Chloro-phenoxy)-5′-fluoro-phenylamino]-methyl)-phenyl)-acetamide (**3**)

Using the general procedure for the reductive amination of the substituted diphenyl ether aniline with the substituted 2-acetamide benzaldehyde, the desired compound was isolated as a white solid, 68 mg, 27% yield. R.f. 0.4 (1:1, EtOAc: Hexane), m.p. 178–180 °C, LCMS: *t*_r_ = 0.98 min (95% MeOH in H_2_O), m/z M-H 383.28, HPLC: *t*_r_ = 2.87 min (90% acetonitrile in H_2_O), 99%, ^1^H NMR (CDCl_3_, 270 MHz): δ 2.03 (3H, s, CH_3_), 4.23 (2H, d, *J* = 5.0 Hz, CH_2_), 4.42 (1H, t, *J* = 4.9 Hz, NH), 6.41–6.58 (2H, m, ArH), 6.80–6.88 (3H, m, ArH), 7.10 (1H, t, *J* = 7.2 Hz, ArH), 7.22–7.32 (4H, m, ArH), 7.86 (1H, d, *J* = 7.9 Hz, ArH), 8.19 (1H, br.s, NHCO). ^13^C NMR (CDCl_3_, 68 MHz): δ 24.3 (CH_3_), 46.8 (CH_2_), 100.7 (d, *J* = 28.1 Hz, ArH), 104.6 (d, *J* = 23.7 Hz, ArCH), 118.0 (ArCH), 120.8 (d, *J* = 10.0 Hz, ArCH), 123.6 (ArCH), 128.1 (d, *J* = 11.8 Hz, ArC), 128.9, 129.4, 129.9 (ArCH), 136.9, 139.2 (ArC), 141.3 (d, *J* = 10.6 Hz, ArC), 156.3, 158.8, 162.3 (ArC), 168.7 (CO). ^19^F NMR (CDCl_3_, 376 MHz): δ ^-^115.43–115.57 (m, ArF). HRMS: Calcd. for C_21_H_18_ClFN_2_O_2_ (M + H)^+^ 383.0968, found (M + H)^+^ 383.0965.

##### 6-Amino-benzo[1,3]dioxole-5-carbaldehyde

Using the general procedure for the reduction of substituted 2-nitrobenzylalcohol, the desired compound was obtained as a brown solid, 360 mg, 85% yield. R.f. 0.67 (DCM), ^1^H NMR (CDCl_3_, 270 MHz,): δ 5.90 (2H, s, CH_2_), 6.11 (1H, s, ArH), 6.29 (2H, br.s, NH), 6.79 (1H, s, ArH), 9.57 (1H, s, CHO).

##### *N*-(6-Formyl-benzo[1,3]dioxol-5-yl)-acetamide

Using the general procedure for the acylation of substituted 2-aminobenzylalcohols, the title compound was obtained as a dark yellow solid, 180 mg, 78% yield. m.p. 133–137 °C, R.f. 0.35 (DCM), m.p. 133–137 °C, ^1^H NMR (CDCl_3_, 270 MHz,): δ 2.21 (3H, s, CH_3_), 6.05 (2H, s, CH_2_), 6.98 (1H, s, ArH), 8.34 (1H, s, ArH), 9.65 (1H, s, CHO), 11.46 (1H, s, NH).

##### *N*-(6-[2-(4-Chloro-phenoxy)-phenylamino]-methyl-benzo[1,3]dioxol-5-yl)-acetamide (**4**)

Using the general procedure for the reductive amination of the substituted diphenyl ether aniline with the substituted 2-acetamide benzaldehyde, the desired compound was isolated as a light cream solid, 540 mg, 29% yield. R.f. 0.75 (DCM), m.p. 154–156 °C, LCMS: *t*_r_ = 1.3 min (95% MeOH in water), m/z M^-^H 409.45, HPLC: *t*_r_ = 4.7 min (90% acetonitrile in H_2_O), 98%, ^1^H NMR (CDCl_3_, 400 MHz,): δ 1.95 (3H, s, CH_3_), 4.18 (2H, s, NHCH_2_), 4.23 (1H, s, NH), 5.93 (2H, s, CH_2_O), 6.74 (1H, s, ArH), 6.77–6.81 (1H m, ArH), 6.86–6.89 (3H, m, ArH), 7.10 (1H, td, *J* = 4.0, 8.8 Hz, ArH), 7.24–7.27 (2H, m, ArH), 7.44 (1H, s, ArH), 8.23 (1H, s, ArH). ^13^C NMR (CDCl_3_, 101 MHz): δ 24.2 (CH_3_), 47.2 (CH_2_NH), 101.4 (CH_2_O), 105.3, 109.1, 113.4, 118.5, 119.2, 119.4 (ArCH), 121.7 (ArC), 125.4 (ArCH), 128.0 (ArC), 129.8 (ArCH), 131.1, 139.6, 143.7, 144.7, 147.4, 155.9 (ArC), 168.3 (CO). HRMS: Calcd. for C_22_H_19_ClN_2_O_4_ (M + H)^+^ 409.0961, found (M + H)^+^ 409.0957.

##### (2-Amino-4,5-dimethoxy-phenyl)-methanol

Using the general procedure for the reduction of the substituted 2-nitrobenzylalcohol, the desired compound was obtained as a brown oil, 764 mg, 93% yield. R.f. 0.17 (EtOAc), ^1^H NMR (CDCl_3_, 270 MHz,): δ 3.30 (2H, br.s, NH_2_), 3.78 (3H, s, OCH_3_), 3.81 (3H, s, OCH_3_), 4.58 (2H, s, CH_2_), 6.29 (1H, s, ArH), 6.63 (1H, s, ArH).

##### *N*-(2-Hydroxymethyl-4,5-dimethoxy-phenyl)-acetamide

Using the general procedure for the acylation of substituted 2-aminobenzylalcohols, the title compound was obtained as a yellow, waxy solid, 277 mg, 59% yield. R.f. 0.48 (EtOAc), ^1^H NMR (CDCl_3_, 270 MHz,): δ 2.14 (3H, s, CH_3_), 2.85, (1H, br.s, OH), 3.82 (3H, s, OCH_3_), 3.84 (3H, s, OCH_3_), 4.56 (2H, s, CH_2_), 6.69 (1H, s, ArH), 7.46 (1H, s, ArH).

##### *N*-(2-Formyl-4,5-dimethoxy-phenyl)-acetamide

Using the general procedure for the Dess–Martin periodinane oxidation of alcohols, the title compound was obtained as a yellow solid, 137 mg, 50% yield. R.f. 0.22 (EtOAc), m.p. 175–178 °C, HPLC *t*r = 1.05 min (90% acetonitrile in H_2_O) 96%, LCMS *t*r = 0.86 min (95% MeOH in H_2_O) M-H 221.77, ^1^H NMR (CDCl_3_, 270 MHz): δ 2.23 (3H, s, CH_3_), 3.90 (3H, s, OCH_3_), 3.98 (3H, s, OCH_3_), 7.02 (1H, s, ArH), 8.46 (1H, s, ArH), 9.74 (1H, s, CHO), 11.32 (1H, br.s, NH).

##### *N*-(2-[2-(4-Chloro-phenoxy)-phenylamino]-methyl-4,5-dimethoxy-phenyl)-acetamide (**5**)

Using the general procedure for the reductive amination of the substituted diphenyl ether aniline with the substituted 2-acetamide benzaldehyde, the resulting reaction mixture was stirred at r.t. for 2 h, then it was subjected to microwave heating for 5 min at 140 °C. NaHCO_3_ was added, and the mixture was repeatedly extracted with DCM. The organic layers were combined and dried (MgSO_4_), filtered and evaporated in vacuo. The crude mixture was purified using flash chromatography (100% EtOAc) to afford the title compound as a cream solid, 20 mg, 11% yield. R.f. 0.44 (EtOAc), m.p. 117–118 °C, LCMS: *t*_r_ = 0.97 min (95% MeOH in water), m/z M-H 425.16, HPLC: *t*_r_ = 1.99 min (90% acetonitrile in H_2_O), 98%, ^1^H NMR (CDCl_3_, 270 MHz,): δ 1.94 (3H, s, CH_3_), 3.81 (3H, s, OCH_3_), 3.85 (3H, s, OCH_3_), 4.22 (3H, br.s, CH_2_ and NH), 6.74–6.90 (6H, m, ArH), 7.06–7.12 (1H, m, ArH), 7.21–7.26 (2H, m, ArH), 7.57 (1H, s, ArH), 8.26 (1H, br.s, NHCO). ^13^C NMR (CDCl_3_, 68 MHz): δ 24.4 (CH_3_), 47.2 (CH_2_), 56.11, 56.27 (OCH_3_), 107.4, 112.5, 113.6, 118.5, 119.3, 119.6 (ArCH), 120.2 (ArC), 125.6 (ArCH), 128.1 (ArC), 129.9, (ArCH), 130.6, 139.9, 143.7, 148.7, 156.0 (ArC), 168.5 (CO). HRMS: Calcd. for C_23_H_23_ClN_2_O_4_ (M + Na)^+^ 449.1230, found (M + Na)^+^ 449.1239.

##### *N*-(2-([2-(2,4-Dichloro-phenoxy)-phenylamino]-methyl)-4,5-dimethoxy-phenyl)-acetamide (**6**)

Here, 2-(2, 4-Dichloro-phenoxy)-phenylamine hydrochloride (135 mg, 0.47 mmol) was dissolved in DCM (10 mL), and K_2_CO_3_ (128 mg, 0.94 mmol) was added; the reaction was then stirred at r.t. for 30min. H_2_O was added to the reaction and the mixture was extracted with DCM. The organic layers were combined and dried (MgSO_4_), filtered and evaporated in vacuo. The resulting free amine was dissolved in DCE (2 mL) and to this N-(2-formyl-4,5-dimethoxy-phenyl)-acetamide (69 mg, 0.31 mmol) acetic acid (0.12 mL) and sodium tri-acetoxy borohydride (164 mg, 0.8 mmol) were added. The resulting reaction mixture was stirred at r.t. for 2 h. NaHCO_3_ was then added, and repeatedly extracted with DCM. The organic layers were combined and dried (MgSO_4_), filtered and evaporated in vacuo. The crude mixture was purified using flash chromatography (0–50% EtOAc in hexane) to afford the title compound as an off-white solid, 55 mg, 38% yield. R.f. 0.58 (EtOAc), m.p. 128–131 °C, LCMS: *t*_r_ = 1.1 min (95% MeOH in water), m/z M-H 459.24, HPLC: *t*_r_ = 2.2 min (90% acetonitrile in H_2_O), 96%, ^1^H NMR (CDCl_3_, 270 MHz,): δ 1.98 (3H, s, CH_3_), 3.83 (3H, s, OCH_3_), 3.87 (3H, s, OCH_3_), 4.26 (2H, s, CH_2_), 4.34 (1H, br.s, NH), 6.75–6.77 (3H, m, ArH), 6.83–6.91 (2H, m, ArH), 7.02–7.11 (1H, m, ArH), 7.15 (1H, dd, *J* = 1.7, 8.7 Hz, ArH), 7.61 (1H, s, ArH), 8.31 (1H, br.s, NHCO). ^13^C NMR (CDCl_3_, 101 MHz): δ 24.5 (CH_3_), 47.2 (CH_2_), 56.1, 56.3 (OCH_3_), 104.0 (ArC), 107.4, 112.5, 113.6, 118.0, 119.1, 120.0, 125.4 (ArCH), 125.5, 125.8 (ArC), 128.1 (ArCH), 129.0 (ArC), 130.6 (ArCH), 139.1, 143.7, 146.0, 148.7, 151.5 (ArC), 168.5 (CO). HRMS: Calcd. for C_23_H_22_Cl_2_N_2_O_4_ (M + H)^+^ 461.1029, found (M + H)^+^ 461.1028.

##### (1-Nitro-naphthalen-2-yl)-methanol

Using the general procedure for the reduction of substituted 2-nitrobenzaldehyde, the desired product was obtained as a dark yellow solid, 1 g, >99% yield. R.f. 0.59 (EtOAc), m.p 78–80 °C, ^1^H NMR (CDCl_3_, 270 MHz,): δ 2.32 (1H, br.s, OH), 4.82 (2H, s, CH_2_), 7.48–7.65 (3H, m, ArH), 7.81–7.90 (2H, m, ArH), 7.99 (1H, d, *J* = 8.4, ArH).

##### (1-Amino-naphthalen-2-yl)-methanol

Using the general procedure for the reduction of the substituted 2-nitrobenzylalcohol, the desired product was obtained, 730 mg, 86% yield. R.f. 0.62 (EtOAc), LCMS: *t*_r_ = 1.3 min (80% MeOH in water), m/z M-H 171.88, HPLC: *t*_r_ = 2.19 min (70% acetonitrile in water), 87%, ^1^H NMR (CDCl_3_, 270 MHz,): δ 4.85 (2H, s, CH_2_), 7.21–7.25 (2H, m, ArCH), 7.41–7.47 (2H, m, ArH), 7.74–7.85 (2H, m, ArH).

##### *N*-(2-Hydroxymethyl-naphthalen-1-yl)-acetamide

Using the general procedure for the acylation of substituted 2-aminobenzylalcohols, the desired product was obtained as a yellow solid, 377 mg, 82% yield. R.f. 0.58 (EtOAc), m.p. 105–108 °C, HPLC *t*_r_ = 1.45 min (90% acetonitrile in H_2_O) >99%, LCMS *t*_r_ = 0.92 min (95% MeOH in H_2_O) M + Na 237.90, ^1^H NMR (CDCl_3_, 270 MHz,): δ 2.32 (3H, s, CH_3_), 3.29 (1H, br.s, OH), 4.63 (2H, s, CH_2_), 7.40–7.44 (3H, m, ArH), 7.77–7.80 (3H, m, ArH).

##### *N*-(2-Formyl-naphthalen-1-yl)-acetamide

Using the general procedure for the Dess–Martin periodinane oxidation of alcohols, the desired product was obtained, 59 mg, 77% yield. R.f. 0.45 (EtOAc), ^1^H NMR (CDCl_3_, 270 MHz,): δ 2.28 (3H, s, CH_3_), 7.55–7.92 (5H, m, ArH), 7.99–8.03 (1H, m, ArH), 9.31 (1H, br.s, NH), 10.18 (1H, s, CHO).

##### *N*-(2-[2-(4-Chloro-phenoxy)-phenylamino]-methyl-naphthalen-1-yl)-acetamide (**7**)

Using the general procedure for the reductive amination of the substituted diphenyl ether aniline with the substituted 2-acetamide benzaldehyde, the desired compound was isolated, 28 mg, 24% yield. R.f. 0.38 (EtOAc), m.p. 153–155 °C, LCMS: *t*_r_ = 1.14 min (95% MeOH in water), m/z M-H 415.34, HPLC: *t*_r_ = 2.26 min (90% acetonitrile in water), 96%, ^1^H NMR (CDCl_3_, 270 MHz,): δ 2.28 (3H, s, CH_3_), 4.42 (2H, s, CH_2_), 4.53 (1H, br.s, NH), 6.63–6.73 (2H, m, ArH), 6.83–6.91 (3H, m, ArH), 6.97–7.03 (1H, m, ArH), 7.21–7.24 (2H, m, ArH), 7.42–7.51 (4H, m, ArH), 7.75 (1H, d, *J* = 8.4 Hz, ArH), 7.80–7.85 (2H, m, ArH and NHCO). ^13^C NMR (CDCl_3_, 68 MHz): 23.5 (CH_3_), 45.4 (CH_2_), 112.4, 117.7, 118.5, 119.6, 122.6, 125.5, 126.0, 126.1 (ArCH), 127.8 (ArC), 128.2, 128.4, 129.8 (ArCH), 130.5, 130.6, 133.4, 133.7, 140.2, 142.9, 156.5 (ArC), 169.4 (CO). HRMS: Calcd. for C_25_H_21_ClN_2_O_2_ (M + H)^+^ 439.1184, found (M + H)^+^ 439.1190.

##### (2-Amino-5-methyl-phenyl)-methanol

Using the general procedure for the reduction of the substituted 2-nitrobenzylalcohol, the desired compound was obtained as a brown solid, 162 mg, 79% yield. R.f. 0.45 (EtOAc), m.p. 118–121 °C, LCMS: *t*_r_ = 0.93 min (95% MeOH in water), m/z M + H 137.80, HPLC: *t*_r_ = 1.53 min (90% acetonitrile in H_2_O), 96%, ^1^H NMR (CDCl_3_, 270 MHz,): δ 2.22 (3H, s, CH_3_), 4.02 (2H, br.s, NH), 4.64 (2H, s, CH_2_), 6.62 (1H, d, *J* = 9.3 Hz, ArH), 6.89–6.95 (2H, m, ArH), 7.25 (1H, s, OH).

##### *N*-(2-Hydroxymethyl-4-methyl-phenyl)-acetamide

Using the general procedure for the acylation of substituted 2-aminobenzylalcohols, the title compound was obtained as a cream solid, 180 mg, 79% yield. R.f. 0.35 (EtOAc), m.p. 134–136 °C (from hexane), LCMS *t*_r_ = 1.4 min (80% MeOH in H_2_O) M + Na 210.99, ^1^H NMR (CDCl_3_, 270 MHz,): δ 2.17 (3H, s, CH_3_), 2.29 (3H, s, CH_3_), 4.63 (2H, d, *J* = 4.9 Hz, CH_2_), 7.02 (1H, s, ArH), 7.12 (1H, d, *J* = 8.4 Hz, ArH), 7.80 (1H, d, *J* = 8.2 Hz, ArH), 8.28 (1H, s, NH).

##### *N*-(2-Formyl-4-methyl-phenyl)-acetamide

Using the general procedure for the Dess–Martin periodinane oxidation of alcohols, the title compound was obtained as a red oil, 44 mg, 25% yield. R.f. 0.8 (10% MeOH in DCM), ^1^H NMR (CDCl_3_, 270 MHz): δ 2.21 (3H, s, CH_3_), 2.36 (3H, s, CH_3_), 7.37–7.41 (2H, m, ArH), 8.55–8.61 (1H, m, ArH), 9.84 (1H, s, CHO), 10.98 (1H, br.s, NH).

##### *N*-(2-[2-(4-Chloro-phenoxy)-phenylamino]-methyl-4-methyl-phenyl)-acetamide (**8**)

Using the general procedure for the reductive amination of the substituted diphenyl ether aniline with the substituted 2-acetamide benzaldehyde, the desired compound was isolated as a light cream solid, 37 mg, 47% yield. R.f. 0.35 (EtOAc), m.p. 138–140 °C (from hexane), LCMS: *t*_r_ = 1.21 min (95% MeOH in water), m/z M + H 381.20, HPLC: *t*_r_ = 2.38 min (90% acetonitrile in H_2_O), 96%. ^1^H NMR (CDCl_3_, 270 MHz,): δ 1.97 (3H, s, CH_3_), 2.29 (3H, s, CH_3_), 4.25 (3H, s, CH_2_ and NH), 6.76–6.93 (5H, m, ArCH), 7.07–7.13 (3H, m, ArH), 7.22–7.27 (2H, m, ArH), 7.83 (1H, d, *J* = 8.2 Hz, ArH), 8.35 (1H, br.s, NH). ^13^C NMR (CDCl_3_, 68 MHz): δ 20.9, 24.5 (CH_3_), 47.5 (CH_2_), 113.6, 118.6, 119.3, 119.6, 123.1, 125.5 (ArCH), 128.0, 128.1 (ArC), 129.3, 129.9, 130.2 (ArCH), 134.4, 134.7, 139.9, 143.8, 156.0 (ArC), 168.5 (CO). HRMS: Calcd. for C_22_H_21_ClN_2_O_2_ (M + H)^+^ 381.1364, found (M + H)^+^ 381.1365.

##### *N*-(4-Chloro-2-hydroxymethyl-phenyl)-acetamide

Using the general procedure for the acylation of substituted 2-aminobenzylalcohols, the title compound was obtained as a cream wax, 1.12 g, 89% yield. R.f. 0.35 (EtOAc), ^1^H NMR (CDCl_3_, 270 MHz,): δ 1.46 (2H, s, NH and OH), 2.19 (3H, s, CH_3_), 4.60 (2H, s, CH_2_), 7.14–7.26 (2H, m, ArH), 7.94–7.98 (1H, m, ArH).

##### *N*-(4-Chloro-2-formyl-phenyl)-acetamide

Using the general procedure for the Dess–Martin periodinane oxidation of alcohols, the title compound was obtained as a red solid, 383 mg, 70% yield. R.f. 0.72 (10% MeOH in EtOAc), m.p. 152–154 °C, ^1^H NMR (CDCl_3_, 270 MHz): δ 2.24 (3H, s, CH_3_), 7.55 (1H, dd, *J* = 2.5, 8.9 Hz, ArH), 7.61 (1H, dd, *J* = 2.5 Hz, ArH), 8.71 (1H, d, *J* = 9.2 Hz, ArH), 9.84 (1H, s, CHO), 11.00 (1H, br.s, NH).

##### *N*-(4-Chloro-2-[2-(4-chloro-phenoxy)-phenylamino]-methyl-phenyl)-acetamide (**9**)

Using the general procedure for the reductive amination of the substituted diphenyl ether aniline with the substituted 2-acetamide benzaldehyde, the desired compound was isolated as a light brown wax, 68mg, 26% yield. R.f. 0.43 (1:1, EtOAc: Hexane), LCMS: *t*_r_ = 1.21 min (95% MeOH in water), m/z M-H 399.15, 401.1, HPLC: *t*_r_ = 2.62 min (90% acetonitrile in H_2_O), 97%, ^1^H NMR (CDCl_3_, 270 MHz,): δ 1.95 (3H, s, CH_3_), 4.22 (3H, s, NH and CH_2_), 6.79–6.91 (5H, m, ArH), 7.07–7.13 (1H, m, ArH), 7.24–7.28 (4H, m, ArH), 7.98 (1H, d, *J* = 8.4 Hz, ArH), 8.56 (1H, br.s, NHCO). ^13^C NMR (CDCl_3_, 101 MHz): δ 24.5 (CH_3_), 47.5 (CH_2_), 113.8, 118.7, 119.5, 119.9, 124.1, 125.5, 128.7, 129.3 (ArCH), 129.5 (ArC), 129.9 (ArCH), 136.0, 139.4, 144.1, 155.9, 168.5, 200.5 (ArC), 205.1 (CO). HRMS: Calcd. for C_21_H_18_Cl_2_N_2_O_2_ (M + H)^+^ 401.0818, found (M + H)^+^ 401.0803.

##### *N*-(4-Chloro-2-[2-(2,4-dichloro-phenoxy)-phenylamino]-methyl-phenyl)-acetamide (**10**)

Here, 2-(2,4-Dichloro-phenoxy)-phenylamine hydrochloride (350 mg, 1.22 mmol) was dissolved in DCM (10 mL), and K_2_CO_3_ (335 mg, 2.44 mmol) was added; the reaction was then stirred at r.t. for 30 min. H_2_O was added and the mixture was extracted with DCM. The organic layers were combined and dried (MgSO_4_), filtered and evaporated in vacuo. The resulting amine was dissolved in DCE (2 mL) and N-(4-chloro-2-formyl-phenyl)-acetamide (160 mg, 0.81 mmol), acetic acid (0.15 mL) and sodium tri-acetoxy borohydride (0.43 g, 2.02 mmol) were added. The resulting reaction mixture was stirred at r.t. for 2 h. NaHCO_3_ was added and the mixture was repeatedly extracted with DCM. The organic layers were combined and dried (MgSO_4_), filtered and evaporated in vacuo. The crude mixture was purified using flash chromatography (0–50% EtOAc in hexane) to afford the title compound as a cream solid, 210 mg, 60% yield. R.f. 0.38 (EtOAc), m.p. 135–137 °C, LCMS: *t*_r_ = 1.3 min (95% MeOH in water), m/z M-H 433.1, 435.1, HPLC: *t*_r_ = 2.8 min (90% acetonitrile in H_2_O), 99%, ^1^H NMR (CDCl_3_, 270 MHz,): δ 1.98 (3H, s, CH_3_), 4.28–4.29 (2H, m, CH_2_), 4.40 (1H, br.s, NH), 6.70–6.88 (4H, m, ArH), 7.04–7.10 (1H, m, ArH), 7.17 (1H, dd, *J* = 2.5 Hz, ArH), 7.25–7.27 (2H, m, ArH), 7.45 (1H, d, *J* = 2.5 Hz, ArH), 7.96–7.99 (1H, m, ArCH), 8.64 (1H, br.s. NH). ^13^C NMR (CDCl_3_, 101 MHz): δ 24.6 (CH_3_), 47.4 (CH_2_), 113.8, 117.8, 119.7, 120.3, 124.1, 125.3 (ArCH), 125.9 (ArC), 128.2, 128.7, 129.3 (ArCH), 129.4, 129.6 (ArC), 130.7 (ArCH), 136.0, 138.6, 144.4, 151.1 (ArC), 168.5 (CO). HRMS: Calcd. for C_21_H_17_Cl_3_N_2_O_2_ (M + H)^+^ 435.0428, found (M + H)^+^ 437.0387.

##### *N*-(4-Chloro-2-[2-(4-trifluoromethoxy-phenoxy)-phenylamino]-methyl-phenyl)-acetamide (**11**)

Using the general procedure for the reductive amination of the substituted diphenyl ether aniline with the substituted 2-acetamide benzaldehyde, the desired compound was isolated as a brown solid, 86 mg, 30% yield. R.f. 0.35 (1:1, EtOAc: Hexane), m.p. 121–123 °C, LCMS: *t*_r_ = 1.07 min (95% MeOH in water), m/z M-H 449.29, HPLC: *t*_r_ = 3.28 min (90% acetonitrile in H_2_O), >99%, ^1^H NMR (CDCl_3_, 270 MHz,): δ 1.94 (3H, s, CH_3_), 4.23 (3H, s, CH_2_ and NH), 6.80–6.96 (5H, m, ArH), 7.08–7.18 (3H, m, ArH), 7.25–7.30 (3H, m, ArH), 7.99 (1H, dd, *J* = 8.4 Hz, ArH), 8.54 (1H, br.s, NHCO). ^13^C NMR (CDCl_3_, 101 MHz): 24.4 (CH_3_), 47.4 (CH_2_), 113.8, 118.1, 119.6, 119.8, 112.8, 123.9, 125.5, 128.6, 129.2 (ArCH), 135.9, 139.3 (ArC), 155.6 (OCF_3_), 207.9 (CO). ^19^F NMR (CDCl3, 376 MHz): δ −58.3 (OCF_3_). Anal. Calcd. for C_22_H_18_ClF_3_N_2_O_3_: C 58.61, H 4.02, N 6.21%. Found: C 58.1, H 4.12, N 5.96%. HRMS: Calcd. for C_22_H_18_ClF_3_N_2_O_3_ (M + H)^+^ 451.1031, found (M + H)^+^ 451.1024.

##### *N*-(2-Formyl-phenyl)-*N*-methyl-acetamide (**13**)

To a solution of *N*-(2-formyl-phenyl)-acetamide **12** (100 mg, 0.61 mmol) in DMF (10 mL), NaH (60% dispersion in mineral oil, 30 mg, 0.73 mmol) was added. After 1h had elapsed, MeI (0.08 mL, 1.2 mmol) was added and stirred at r.t. under N_2_ for 3 days. This was poured onto water (20 mL), extracted with EtOAc and dried (MgSO_4_). The crude product was purified by flash chromatography (0–100% DCM in hexane) to yield the desired product as a white wax, 60 mg, 56% yield. R.f. 0.35 (DCM), LCMS: *t*_r_ = 1.0 min (95% MeOH in water), m/z M + H 177.80, HPLC: *t*_r_ = 1.0 min (95% MeOH in water), 97%, ^1^H NMR (CDCl_3_, 270 MHz,): δ 1.79 (3H, s, CH_3_CO), 3.28 (3H, s, CH_3_N), 7.26–7.29 (1H, m, ArH), 7.50–7.55 (1H, m, ArH), 7.69 (1H, td, *J* = 1.6, 7.5 Hz, ArH), 7.97 (1H, *J* = 1.6, 7.7 Hz, ArH), 10.13 (1H, s, CHO).

##### *N*-(2-([2-(4-Chloro-phenoxy)-phenylamino]-methyl)-phenyl)-*N*-methyl-acetamide (**14**)

To a solution of 2-(4-chloro-phenoxy)-phenylamine (174 mg, 0.78 mmol) and *N*-(2-formyl-phenyl)-*N*-methyl-acetamide **13** (70 mg, 0.39 mmol) in DCE (2mL), NaHB(OAc)_3_ (210 mg, 0.98 mmol) and AcOH (0.07 mL) were added. The resulting solution was stirred at r.t. for 2 h. NaHCO_3_ was then added, and the mixture was extracted with DCM and dried (MgSO_4_). The crude product was purified by flash chromatography (0–100% DCM in hexane) to yield the desired product as a brown oil, 65 mg, 44% yield. R.f. 0.51 (DCM), LCMS: *t*_r_ = 1.19 min (95% MeOH in water), m/z M-H 378.93, HPLC: *t*_r_ = 2.64 min (90% acetonitrile in water), 98%, ^1^H NMR (CDCl_3_, 270 MHz,): δ 1.78 (3H, s, CH_3_CO), 3.24 (3H, s, CH_3_N), 4.25–4.29 (2H, m, CH_2_), 6.59 (1H, dd, *J* = 1.4, 8.0 Hz, ArH), 6.67 (1H, td, *J* = 1.4, 7.7 Hz, ArH), 6.83 (1H, dd, *J* = 1.4, 7.7 Hz, ArH), 6.88–6.92 (2H, m, ArH), 7.00 (1H, td, *J* = 1.6, 7.7 Hz, ArH), 7.11–7.17 (1H, m, ArH), 7.23–7.26 (2H, m, ArH), 7.31–7.34 (2H, m, ArH), 7.41–7.44 (1H, m, ArH). ^13^C NMR (CDCl_3_, 68 MHz): 26.5, 36.6 (CH_3_), 43.7 (CH_2_), 117.7, 118.7, 119.5, 125.4 (ArCH), 127.9 (ArC), 128.5, 128.9, 129.1, 129.7 (ArCH), 136.3, 139.9, 142.5, 142.8, 156.1 (ArC), 170.8 (CO). HRMS: Calcd. for C_22_H_21_ClN_2_O_2_ (M + H)^+^ 381.1364, found (M + H)^+^ 381.1378.

##### *N*-[2-(([2-(4-Chloro-phenoxy)-phenyl]-methyl-amino)-methyl)-phenyl]-*N*-methyl-acetamide (**15**)

*N*-(2-([2-(4-chloro-phenoxy)-phenylamino]-methyl)-phenyl)-acetamide (100 mg, 0.27 mmol) in DMF (10 mL) was cooled to 0 °C, NaH (35 mg, 0.81 mmol) was added and the resulting solution was stirred for 1 h. MeI (0.05 mL, 0.81 mmol) was added, and the solution stirred for a further 18 h. The reaction mixture was then poured onto water, extracted with EtOAc and dried (MgSO_4_). NMR analysis showed the crude product to be a mixture of product and the related mono-methylated compound. Preparative HPLC was used for purification to yield the desired mono-methylated product as a white wax, 25 mg, 23% yield. Please note *N*-(2-([2-(4-chloro-phenoxy)-phenylamino]-methyl)-phenyl)-*N*-methyl-acetamide was also isolated, 46 mg, 44% yield. R.f. 0.45 (EtOAc), LCMS: *t*_r_ = 5.6 min (80% MeOH in water), m/z M + H 395.18, HPLC: *t*_r_ = 3.75 min (90% acetonitrile in water), 98%, ^1^H NMR (CDCl_3_, 270 MHz,): δ 1.70 (3H, s, CH_3_), 2.66 (3H, s, NCH_3_), 3.09 (3H, s, NCH_3_), 4.10 (2H, s, CH_2_), 6.67–6.73 (2H, m, ArH), 6.96 (2H, d, *J* = 3.9 Hz, ArH), 7.04–7.27 (8H, m, ArH). ^13^C NMR (CDCl_3_, 68 MHz): δ 22.0, 36.3, 39.7 (CH_3_), 54.9 (CH_2_), 117.8, 119.7, 122.2, 122.5, 125.6 (ArCH), 127.1 (ArC), 142.4, 145.0, 147.4 (ArC), 170.8 (CO). HRMS: Calcd. for C_23_H_23_ClN_2_O_2_ (M + H)^+^ 395.1521, found (M + H)^+^ 395.1533.

##### *N*-[2-(([2-(4-Chloro-phenoxy)-phenyl]-methyl-amino)-methyl)-phenyl]-acetamide (**16**)

To a solution of *N*-(2-[2-(4-chloro-phenoxy)-phenylamino]-methyl-phenyl)-acetamide, (100 mg, 0.27 mmol), paraformaldehyde (81 mg, 2.7 mmol) and NaBH_4_ (55 mg, 1.35 mmol) in THF (5 mL) were added TFA (1.3 mL). The resulting solution was stirred at r.t. for 18 h. This was then poured into NaOH solution (25%) with ice chips, extracted with DCM and dried (MgSO_4_). The crude product was then purified by flash chromatography (0–100% EtOAc in hexane) to produce the desired compound as a colourless oil, 64 mg, 62% yield. R.f. 0.66 (EtOAc), LCMS: *t*_r_ = 4.02 min (80% MeOH in water), m/z M + H 379.12, HPLC: *t*_r_ = 2.88 min (90% MeOH in water), 99%, ^1^H NMR (CDCl_3_, 270 MHz,): δ 1.99 (3H, s, CH_3_), 2.65 (3H, s, CH_3_N), 4.19 (2H, s, CH_2_), 6.81 (1H, dd, *J* = 1.6, 8.4 Hz, ArH), 6.93–6.95 (2H, m, ArH), 6.99–7.04 (2H, m, ArH), 7.10 (1H, td, *J* = 1.6, 8.0 Hz, ArH), 7.14–7.15 (1H, m, ArH), 7.23–7.32 (4H, m, ArH), 8.27 (1H, d, *J* = 8.4 Hz, ArH), 10.12 (1H, br.s, NHCO). ^13^C NMR (CDCl_3_, 101 MHz): 24.8, 40.7 (CH_3_), 59.5 (CH_2_), 118.7, 120.3, 120.9, 121.3, 123.2, 124.1, 124.6, (ArCH), 125.1 (ArC), 128.6 (ArC), 130.1 (ArCH), 138.6, 142.4, 150.8, 155.1 (ArC), 168.6 (CO). HRMS: Calcd. for C_22_H_21_ClN_2_O_2_ (M + H)^+^ 381.1364, found (M + H)^+^ 381.1363.

##### *N*-(2-Acetylamino-benzyl)-*N*-[2-(4-chloro-phenoxy)-phenyl]-acetamide (**17**)

A solution of *N*-(2-[2-(4-chloro-phenoxy)-phenylamino]-methyl-phenyl)-acetamide (100 mg, 0.27 mmol) in DCM (5 mL) was cooled to 0 °C, and TEA (0.2 mL) and acetyl chloride (0.34 mL, 0.81 mmol) were added; the resulting solution was stirred at r.t. for 1 h. Saturated NaHCO_3_ was added, extracted with DCM and dried (MgSO_4_). The crude product was purified by flash chromatography (0–100% EtOAc in hexane) and preparative HPLC to yield the desired product as an off white waxy solid, 53 mg, 48% yield. R.f. 0.54 (EtOAc), LCMS: *t*_r_ = 2.17 min (95% MeOH in water), m/z M-H 407.15, HPLC: *t*_r_ = 2.40 min (90% acetonitrile in water), 95%, ^1^H NMR (CDCl_3_, 270 MHz,): δ 1.93 (3H, s, CH_3_), 2.24 (3H, s, CH_3_), 4.80 (2H, s, CH_2_), 7.02–7.12 (2H, m, ArH), 7.21–7.30 (4H, m, ArH), 8.20 (1H, dd, *J* = 7.7 Hz, ArH), 9.89 (1H, s, NHCO). ^13^C NMR (CDCl_3_, 68 MHz): 22.1, 24.6 (CH_3_), 49.8 (CH_2_), 118.1, 120.7, 122.1, 123.0, 124.0 (ArCH), 125.0 (ArC), 129.2 (ArCH), 129.8 (ArC), 130.1, 130.2, 131.5 (ArCH), 137.7, 153.2, 153.8 (ArC), 169.5, 172.7 (CO). HRMS: Calcd. for C_23_H_21_ClN_2_O_3_ (M + Na)^+^ 431.1313, found (M + Na)^+^ 431.1105.

##### *N*-(4-([2-(4-Chloro-phenoxy)-phenylamino]-methyl)-phenyl)-acetamide (**18**)

Using the general procedure for the reductive amination of the substituted diphenyl ether aniline 4-acetamido-benzaldehyde, the desired compound was isolated as a light pink solid, 195 mg, 78% yield. R.f. 0.55 (5% MeOH in DCM), m.p. 137–139 °C, LCMS: *t*_r_ = 1.39 min (95% MeOH in H_2_O), m/z M-H 365.48, HPLC: *t*_r_ = 2.0 min (90% acetonitrile in H_2_O), 98%, ^1^H NMR (CDCl_3_, 270 MHz): δ 2.15 (3H, s, CH_3_), 4.30 (2H, d, *J* = 5.7 Hz, CH_2_), 6.60–6.69 (2H, m, ArH), 6.82 (1H, dd, *J* = 1.5, 7.7 Hz, ArH), 6.86–6.91 (2H, m, ArH), 6.96–7.02 (1H, m, ArH), 7.12 (1H, s, NH), 7.21–7.26 (4H, m, ArH), 7.41–7.44 (2H, m, ArH). ^13^C NMR (CDCl_3_, 101 MHz): δ 24.6 (CH_3_), 47.2 (CH_2_), 111.9, 117.1, 118.5, 119.3, 120.1, 125.3 (ArCH), 127.6 (ArC), 127.9, 129.6 (ArCH), 135.1, 136.8, 140.1, 142.6, 156.2 (ArC), 168.2 (CO). HRMS: Calcd. for C_21_H_19_ClN_2_O_2_ (M + Na)^+^ 389.1025, found (M + Na)^+^ 389.1028. Anal. Calcd. for C_21_H_19_ClN_2_O_2_: C 68.76, H 5.22 N 7.64%. Found: C 68.5, H 5.26, N 7.61%.

##### *N*-(4-([2-(2,4-Dichloro-phenoxy)-phenylamino]-methyl)-phenyl)-acetamide (**19**)

To a solution of 2-(2,4-dichloro-phenoxy)-phenylamine hydrochloride (0.15 g, 0.52 mmol) in DCM (10 mL), K_2_CO_3_ (0.22 g, 1.04 mmol) was added,;the resulting solution was stirred at r.t for 30 min. Water was added and the mixture was extracted with DCM. The organic layers were combined and dried (MgSO_4_), filtered and evaporated in vacuo. The resulting amine was dissolved in DCE (3 mL) and 4-acetamidobenzaldehyde (0.126 g, 0.0.75 mmol), acetic acid (0.11 mL) and sodium tri-acetoxy borohydride (0.27 g, 1.3 mmol) were added. The resulting reaction mixture was stirred at r.t. for 2 h. NaHCO_3_ was added, and the mixture was repeatedly extracted with DCM. The organic layers were combined and dried (MgSO_4_), filtered and evaporated in vacuo. The crude mixture was purified using flash chromatography (0–100% EtOAc in hexane) to afford the title compound as a white wax, 142 mg, 69% yield. R.f. 5.8 (EtOAc), LCMS: *t*_r_ = 1.2 min (95% MeOH in H_2_O), m/z M-H 399.03, 401.04, HPLC: *t*_r_ = 2.42 min (90% acetonitrile in H_2_O), 98%, ^1^H NMR (CDCl_3_, 270 MHz): δ 2.15 (3H, s, CH_3_), 4.31 (2H, s, CH_2_), 4.59 (1H, br.s, NH), 6.59–6.68 (2H, m, ArH), 6.75 (1H, dd, *J* = 1.5, 7.9 Hz, ArH), 6.80 (1H, d, *J* = 8.7 Hz, ArH), 6.96–7.02 (1H, m, ArH), 7.12 (1H, dd, *J* = 2.5, 8.9 Hz, ArH), 7.22–7.31 (1H, m, ArH), 7.41–7.45 (2H, m, ArH). ^13^C NMR (CDCl_3_, 68 MHz): δ 24.7 (CH_3_), 47.3 (CH_2_), 112.2, 117.1, 118.5, 119.4, 120.2 (ArCH), 125.3 (ArC), 125.5, 127.9, 128.0 (ArCH), 128.2 (ArC), 130.4 (ArCH), 135.1, 136.9, 139.7, 142.7, 151.8 (ArC), 168.4 (CO). HRMS: Calcd. for C_21_H_18_Cl_2_N_2_O_2_ (M + H)^+^ 399.0673, found (M + H)^+^ 399.0674. Anal. Calcd. for C_21_H_18_Cl_2_N_2_O_2_ C 62.85, H 4.52 N 6.98%. Found: C 62.7, H 4.52, N 6.92%.

##### 3-Amino-benzaldehyde

Using the general procedure for the reduction of the substituted 2-nitrobenzylalcohol, the desired compound was obtained as a yellow solid, 1.7 g, 71% yield. R.f. 0.25 (DCM). Due to the instability of this compound, the product was used crude in the following reactions.

##### *N*-(3-Formyl-phenyl)-acetamide

Using the general procedure for the acylation of substituted 2-aminobenzylalcohols, the title compound was obtained as a cream oil, 250 mg, 37% yield. R.f. 0.43 (10% MeOH in DCM), ^1^H NMR (CDCl_3_, 270 MHz,): δ 2.19 (3H, s, CH_3_), 7.42 (1H, t, *J* = 7.9 Hz, ArH), 7.56 (1H, d, *J* = 7.7 Hz, ArH), 7.83–7.86 (1H, m, ArH), 8.03 (1H, s, ArH), 8.60 (1H, s, NH), 9.90 (1H, s, CHO).

##### *N*-(3-[2-(4-Chloro-phenoxy)-phenylamino]-methyl-phenyl)-acetamide (**20**)

Using the general procedure for the reductive amination of the substituted diphenyl ether aniline 3-acetamide benzaldehyde, the desired compound was isolated as a cream solid, 110 mg, 63% yield. R.f. 0.6 (EtOAc), m.p. 187–190 °C, LCMS: *t*_r_ = 1.39 min (50% to 95% MeOH in water at 0.5 mL/min to 1.0 mL/min over 5 min), m/z M-H 365.55, HPLC: *t*_r_ = 1.89 min (90% acetonitrile in H_2_O), 93%, ^1^H NMR (CDCl_3_, 270 MHz,): δ 2.13 (3H, s, CH_3_), 4.32 (2H, d, *J* = 5.4 Hz, CH_2_), 4.55 (1H, d, *J* = 5.4 Hz, NH), 6.61–6.66 (2H, m, ArH), 6.81–6.84 (1H, m, ArH), 6.68–6.92 (2H, m, ArH), 6.98–7.04 (2H, m, ArH), 7.21–7.27 (4H, m, ArH and NH), 7.38–7.43 (2H, m, ArH). ^13^C NMR (CDCl_3_, 68 MHz): δ 24.6 (CH_3_), 47.5 (CH_2_), 112.1, 117.2, 118.4, 118.7, 119.4, 123.0, 125.4 (ArCH), 127.7 (ArC), 129.0, 129.7, 129.9 (ArC), 138.2, 140.1, 140.4, 142.7, 156.3 (ArC), 168.5 (CO). HRMS: Calcd. for C_21_H_19_ClN_2_O_2_ (M + Na)^+^ 389.1027, found (M + Na)^+^ 389.1021.

##### *N*-(2-([2-(4-Chloro-phenoxy)-phenylimino]-methyl)-phenyl)-acetamide

A solution of 2-(4-chloro-phenoxy)-phenylamine (100 mg, 0.46 mmol) and *N*-(2-formyl-phenyl)-acetamide **12** (74 mg, 0.46 mmol) in anhydrous DCM (5mL) was stirred at r.t., MgSO_4_ (550 mg, 4.6 mmol) was added and the resulting mixture stirred for a further 18 h at r.t. The mixture was then filtered and the solid was washed with DCM. The filtrate was then evaporated to dryness to yield the desired product as an oil. The product was identified by NMR, as no CHO peak was visible in the ^1^H NMR and it had been replaced with an imine peak. The product was used crude in all following experiments. A small sample was purified for analytical purposes. ^1^H NMR (CDCl_3_, 270 MHz,): δ 2.03 (3H, s, CH_3_), 6.87–7.46 (11H, m, ArH), 8.60 (1H, s, N=CH), 8.72 (1H, d, *J* = 8.5 Hz, ArH). ^13^C NMR (CDCl_3_, 101 MHz): 24.9 (CH_3_), 116.7, 119.2, 119.6, 120.3 (ArCH), 120.6 (ArC), 122.5, 125.0, 127.9 (ArCH), 128.2 (ArC), 129.8, 132.7 (ArCH), 140.4, 141.7, 149.6, 156.1 (ArC), 163.4 (CH), 169.9 (CO).

##### *N*-(2-([2-(4-Chloro-phenoxy)-phenylimino]-methyl)-phenyl)-acetamide

A solution of 2-(4-chloro-phenoxy)-phenylamine (100 mg, 0.46 mmol) and *N*-(2-formyl-phenyl)-acetamide **12** (74 mg, 0.46 mmol) in anhydrous DCM (5 mL) was stirred at r.t., and TiCl(O^i^Pr)_3_ (0.25 mL, 1 mmol) was added. The resulting mixture was stirred for a further 4 h at room temperature. The mixture was then evaporated to dryness to yield the desired product as an oil. The product was used crude in all subsequent experiments.

##### [2-(4-Chloro-phenoxy)-phenyl]-2-nitro-benzylamine

To a solution of 2-(4-chloro-phenoxy)-phenylamine (150 mg, 0.68 mmol) and 2-nitrobenzaldehyde (310 mg, 2.04 mmol) in DCE (3.5 mL), acetic acid (0.36 mL) and sodium tri-acetoxy borohydride (0.36 g, 1.7 mmol) were added. The resulting reaction mixture was heated in a microwave at 140 °C for 10 min. NaHCO_3_ was then added and the mixture was repeatedly extracted with EtOAc. The organic layers were combined and dried (MgSO_4_), filtered and evaporated in vacuo. The crude mixture was purified using flash chromatography (0–100% EtOAc in hexane) to afford the title compound as a yellow wax, 194 mg, 77% yield. R.f. 0.63 (1:1, EtOAc: Hexane), LCMS: *t*_r_ = 1.66 min (95% MeOH in H_2_O), m/z M + H 355.48, HPLC: *t*_r_ = 6.6 min (90% acetonitrile in H_2_O), 92%, ^1^H NMR (CDCl_3_, 270 MHz): δ 4.75 (2H, s, CH_2_), 4.97 (1H, s, NH), 6.52 (1H, dd, *J* = 1.2, 7.9 Hz, ArH), 6.66 (1H, td, *J* = 1.5, 7.7 Hz, ArH), 6.83–6.99 (4H, m, ArH), 7.21–7.27 (2H, m, ArH), 7.37–7.44 (1H, m, ArH), 7.54–7.57 (2H, m, ArH), 8.05 (1H, dd, *J* = 1.0, 7.7 Hz, ArH).

##### *N*-[2-(4-Chloro-phenoxy)-phenyl]-*N*-(2-nitro-benzyl)-acetamide

Using the general procedure for the acylation of substituted 2-aminobenzylalcohols, the title compound was obtained as a brown oil, 92 mg, 48% yield. R.f. 0.21 (1:1, DCM:hexane), LCMS: *t*_r_ = 5.12 min (50% to 95% MeOH in water at 0.5 mL/min to 1.0 mL/min over 5 min), m/z M + H 397.48, HPLC: *t*_r_ = 5.0 min (90% acetonitrile in H_2_O), 91%, ^1^H NMR (CDCl_3_, 270 MHz,): δ 1.98 (3H, s, CH_3_), 5.13 (1H, d, *J* = 16.3 Hz, ½CH_2_), 5.33 (1H, d, *J* = 16.3 Hz, ½CH_2_), 6.79–6.87 (3H, m, ArH), 7.00–7.11 (2H, m, ArH), 7.20–7.35 (4H, m, ArH), 7.46 (1H, td, *J* = 1.5, 7.4 Hz, ArH), 7.72 (1H, dd, *J* = 1.2, 7.9 Hz, ArH), 7.83 (1H, dd, *J* = 1.2, 8.2 Hz, ArH).

##### *N*-(2-Amino-benzyl)-*N*-[2-(4-chloro-phenoxy)-phenyl]-acetamide

Using the general procedure for the reduction of the substituted 2-nitrobenzylalcohol, the desired compound was obtained as a pale-yellow solid, 53 mg, 62% yield. R.f. 0.25 (1:1, DCM:EtOAc), ^1^H NMR (CDCl_3_, 270 MHz,): δ 1.92 (3H, s, CH_3_), 4.55 (2H, br.s, NH_2_), 4.73–4.87 (2H, m, CH_2_), 6.38–6.52 (3H, m, ArH), 6.58–6.64 (2H, m, ArH), 6.77 (1H, d, *J* = 8.2 Hz, ArH), 6.96–7.05 (3H, m, ArH), 7.19–7.25 (3H, m, ArH). ^13^C NMR (CDCl_3_, 68 MHz): δ 22.2 (CH_3_), 49.3 (CH_2_), 115.41, 116.7, 118.3 (ArCH), 119.8 (ArC), 120.7, 123.8, 129.3, 129.6, 129.9, 130.4, 131.9 (ArCH), 132.1, 146.5, 153.4, 154.4 (ArC), 171.7 (CO).

##### [2-(4-Chloro-phenoxy)-phenyl]-(2-amino-benzyl)-amine

Using the general procedure for the reduction of the substituted 2-nitrobenzylalcohol, the desired compound was obtained as a cream solid, 118 mg, >100% yield. R.f. 0.35 (EtOAc), m.p. 178–180 °C (from hexane), LCMS: *t*_r_ = 5.51 min (50% to 95% MeOH in water at 0.5 mL/min to 1.0 mL/min over 5 min), m/z M-H 323.4, HPLC: *t*_r_ = 5.95 min (90% acetonitrile in H_2_O), 85%, ^1^H NMR (CDCl_3_, 270 MHz,): δ 3.99 (2H, s, NH_2_), 4.16 (1H, s, NH), 4.23 (2H, s, CH_2_), 6.66–6.77 (3H, m, ArH), 6.83–6.91 (4H, m, ArH), 7.07–7.16 (3H, m, ArH), 7.20–7.26 (2H, m, ArH).

##### 1-[4-(2-[2-(4-Chloro-phenoxy)-phenylamino]-methyl-phenylamino)-piperidin-1-yl]-ethanone (**21**)

To a solution of [2-(4-chloro-phenoxy)-phenyl]-(2-amino-benzyl)-amine (50 mg, 0.15 mmol) and *N*-benzoyl-4-piperidone (0.038 mL, 0.30 mmol) in DCE (1.5 mL), acetic acid (0.03 mL) and sodium tri-acetoxyborohydride (82 mg, 0.38 mmol) were added. The resulting reaction mixture was then subjected to microwave heating for 20 min at 140 °C. A further portion of sodium tri-acetoxy borohydride (0.45 g, 0.2 mmol) was added, and the solution was subjected to microwave heating for a further 10 min at 140 °C. NaHCO_3_ was added and the mixture was repeatedly extracted with DCM. The organic layers were combined and dried (MgSO_4_), filtered and evaporated in vacuo. The crude mixture was purified using flash chromatography (0–10% MeOH in DCM) to afford the title compound as a cream oil, 23 mg, 33% yield. R.f. 0.2 (1:1, EtOAc: Hexane), LCMS: *t*_r_ = 5.75 min (50% to 95% MeOH in water at 0.5 mL/min to 1.0 mL/min over 5 min), m/z M + Na 472.41, HPLC: *t*_r_ = 6.19 min (90% acetonitrile in H_2_O), 96%, ^1^H NMR (CDCl_3_, 400 MHz): δ 1.25 (2H, s, CH_2_), 1.83–1.92 (2H, m, CH_2_), 2.07 (3H, s, CH_3_), 2.98–3.04 (1H, m, ½CH_2_), 3.11–3.17 (1H, m, ½CH_2_), 3.47–3.51 (1H, m, ½CH_2_), 3.56–3.62 (1H, m, ½CH_2_), 4.09–4.17 (1H, m, NH), 4.21 (2H, td, *J* = 9.6 Hz, CH_2_NH), 4.70 (1H, s, NH), 6.65–6.71 (2H, m, ArH), 6.75 (1H, td, *J* = 1.6, 7.6 Hz, ArH), 6.81–6.86 (3H, m, ArH), 6.92 (1H, dd, *J* = 1.2, 8.0 Hz, ArH), 7.12 (1H, td, *J* = 1.2, 8.0 Hz, ArH), 7.15–7.17 (1H, m, ArH), 7.19–7.23 (3H, m, ArH). ^13^C NMR (CDCl_3_, 101 MHz): δ 21.5 (CH_3_), 29.7, 32.1, 39.7, 44.6 (CH_2_), 47.5 (CH_2_NH), 48.7 (CH), 110.9, 112.8, 116.8, 118.4, 119.4 (ArCH), 122.1 (ArC), 125.4 (ArCH), 127.7 (ArC), 129.2, 129.6, 130.4 (ArCH), 140.2, 143.3, 145.9, 156.1 (ArC), 168.8 (CO). HRMS: Calcd. for C_26_H_28_ClN_3_O_2_ (M + H)^+^ 450.1943, found (M + H)^+^ 450.1943.

##### *N*-[2-(1-Acetyl-piperidin-4-ylamino)-benzyl]-N-[2-(4-chloro-phenoxy)-phenyl]-acetamide (**22**)

Using the general procedure for the reductive amination of the substituted diphenyl ether aniline with the substituted 2-acetamide benzaldehyde, the desired compound was isolated as a cream oil, 45 mg, 63% yield. R.f. 0.72 (10% MeOH in EtOAc), LCMS: *t*_r_ = 5.4 min (50% to 95% MeOH in water at 0.5 mL/min to 1.0 mL/min over 5 min), m/z M + H 492.49, HPLC: *t*_r_ = 6.42 min (90% acetonitrile in H_2_O), 99%, ^1^H NMR (CDCl_3_, 400 MHz): δ (Multiple signals observed due to restricted rotation and, therefore, the presence of rotamers) 1.34–1.51 (1H, m, ½CH_2_),1.55–1.66 (1H, m, ½CH_2_),1.75–1.79 (1H, m, ½CH_2_), 1.91, 1.93 (3H, s, CH_3_), 1.96–2.01 (1H, m, ½CH_2_), 2.08, 2.09 (3H, s, CH_3_), 2.91–2.97 (½H, m, ¼CH_2_), 3.02–3.09 (½H, m, ¼CH_2_), 3.11–3.18 (½H, m, ¼CH_2_), 3.21–3.27 (½H, m, ¼CH_2_), 3.33–3.41 (1H, m, CH), 3.68–3.74 (½H, m, ¼CH_2_), 3.81–3.86 (½H, m, ¼CH_2_), 3.99–4.04 (½H, m, ¼CH_2_), 4.23–4.28 (½H, m, ¼CH_2_), 4.36 (½H, d, *J* = 14.4 Hz, ¼CH_2_), 4.65 (½H, d, *J* = 14.8 Hz, ¼CH_2_), 4.92 (½H, d, *J* = 14.4 Hz, ¼CH_2_), 5.25 (½H, d, *J* = 14.4 Hz, ¼CH_2_), 5.58–5.66 (1H, m, NH), 6.30–6.37 (3H, m, ArH), 6.44–6.50 (2H, m, ArH), 6.63–6.66 (½H, m, ArH), 6.72–6.74 (½H, m, ArH), 7.03–7.12 (3H, m, ArH), 7.13–7.16 (1H, m, ArH), 7.17–7.23 (2H, m, ArH). ^13^C NMR (CDCl_3_, 101 MHz): δ 21.5, 21.5, 22.0 (CH_3_), 30.6, 31.3, 31.9, 32.3, 39.5, 39.7, 44.7 (CH_2_), 48.4, 48.7 (CH), 49.5, 49.9 (CH_2_), 110.1, 110.2, 114.9, 115., 117.8, 118.1, 119.6, 119.7, 120.5, 120.9, 123.7, 123.8, 129.1, 129.1, 129.3, 129.4, 129.6, 129.7, 129.9 (ArCH), 131.6, 131.7 (ArC), 132.1, 132.2 (ArCH), 145.8, 145.9, 153.3, 153.8, 154.0, 154.3 (ArC), 168.7, 168.7, 171.6, 171.7 (CO). HRMS: Calcd. for C_28_H_30_ClN_3_O_3_ (M + H)^+^ 492.2048, found (M + H)^+^ 492.2049.

##### 1-Acetyl-piperidine-4-carboxylic acid (2-[2-(4-chloro-phenoxy)-phenyl amino]-methyl-phenyl)-amide (**23**)

To a solution of [2-(4-chloro-phenoxy)-phenyl]- (2-amino-benzyl)-amine (48 mg, 0.15 mmol) and TEA (0.09 mL) in DCM (6 mL) at 0 °C, 1-acetylpiperidine-4-carbonyl chloride (58 mg, 0.6 mmol) was added and the resulting solution was stirred, allowed to warm to room temperature and further stirred for 24 h. NaHCO_3_ was added and the mixture was extracted with DCM. The organic portions were then washed with 1 M HCl. The organic layers were combined and dried (MgSO_4_), filtered and evaporated in vacuo. The title compound was obtained as an off-white solid, 44 mg, 62% yield. R.f. 0.15 (10% MeOH in DCM), m.p. 140–143 °C (from hexane), LCMS: *t*_r_ = 1.25 min (95% MeOH in water), m/z M-H 476.56 HPLC: *t*_r_ = 1.71 min (90% acetonitrile in H_2_O), 95%, ^1^H NMR (CDCl_3_, 400 MHz): δ 1.49–1.63 (2H, m, CH_2_), 1.74–1.83 (2H, m, CH_2_), 1.99 (3H, s, CH_3_), 2.18–2.24 (1H, m, CH), 2.49–2.57 (1H, m, CH_2_), 2.92–2.99 (1H, m, CH_2_), 3.69–3.73 (1H, m, CH_2_), 4.28 (3H, s, CH_2_NH and NH), 4.42–4.46 (1H, m, CH_2_), 6.77–6.86 (3H, m, ArH), 6.92–6.94 (1H, m, ArH), 7.06–7.10 (2H, m, ArH), 7.22–7.33 (5H, m, ArH), 8.05 (1H, d, *J* = 8.0 Hz, ArH), 8.85 (1H, s, NHCO). ^13^C NMR (CDCl_3_, 101 MHz): δ 21.4 (CH_3_), 28.5, 28.7, 40.8 (CH_2_), 43.8 (CH), 45.6, 47.8 (CH_2_), 113.5, 118.7, 119.2, 119.7, 122.4, 124.5, 125.3 (ArCH), 127.2, 128.2 (ArC), 128.9, 129.7, 129.8 (ArCH), 137.4, 139.3, 144.0, 155.7 (ArC), 168.7, 172.1 (CO). HRMS: Calcd. for C_27_H_28_ClN_3_O_3_ (M + Na)^+^ 500.1711, found (M + Na)^+^ 500.1705.

##### 1-Acetyl-piperidine-4-carboxylic acid (3-formyl-phenyl)-amide

To a solution of 3-amino-benzaldehyde (200 mg, 1.65 mmol) and TEA (0.13 mL) in DCM (4 mL) at 0 °C, 1-acetylpiperidine-4-carbonyl chloride (0.6 g, 3.3 mmol) was added and the resulting solution was allowed to warm to room temperature and stirred for 2 days. NaHCO_3_ was added and the mixture was repeatedly extracted with DCM; the organic layers were then washed with HCl (1 M). The organic layers were combined and dried (MgSO_4_), filtered and evaporated in vacuo. The title compound was obtained as a cream oil, 97 mg, 22% yield. R.f. 0.42 (10% MeOH in DCM), LCMS: *t*_r_ = 0.99 min (95% MeOH in water), m/z M-H 273.39, HPLC: *t*_r_ = 1.26 min (90% acetonitrile in H_2_O), 81%, ^1^H NMR (CDCl_3_, 270 MHz,): δ 1.66–1.90 (2H, m, CH_2_), 1.95–2.03 (2H, m, CH_2_), 2.11 (3H, s, CH_3_), 2.50–2.61 (1H, m, CH), 2.66–2.76 (1H, m, ½CH_2_), 3.09–3.20 (1H, m, ½CH_2_), 3.89–3.94 (1H, m, ½CH_2_), 4.61–4.65 (1H, m, ½CH_2_), 7.48 (1H, t, *J* = 7.9 Hz, ArH), 7.61 (1H, td, *J* = 1.2, 7.7 Hz, ArH), 7.90–8.00 (4H, m, ArH and NH), 9.97 (1H, s, CHO).

##### 1-Acetyl-piperidine-4-carboxylic acid (3-[2-(4-chloro-phenoxy)-phenyl amino]-methyl-phenyl)-amide (**24**)

Using the general procedure for the reductive amination of the substituted diphenyl ether aniline with the substituted 2-acetamide benzaldehyde, the desired compound was isolated as a cream wax, 70 mg, 41% yield. R.f. 0.22 (10% MeOH in EtOAc), LCMS: *t*_r_ = 5.5 min (50% to 95% MeOH in water at 0.5 mL/min to 1.0 mL/min over 5 min), m/z M-H 476.42, HPLC: *t*_r_ = 1.73 min (90% acetonitrile in H_2_O), 93%, ^1^H NMR (CDCl_3_, 270 MHz,): δ 1.63–1.77 (2H, m, CH_2_), 1.78–1.90 (2H, m, CH_2_), 2.09 (3H, s, CH_3_), 2.38–2.50 (1H, m, CH), 2.63–2.73 (1H, m, ½CH_2_), 3.11 (1H, td, *J* = 2.7, 13.9 Hz, ½CH_2_), 3.88 (1H, d, *J* = 13.6 Hz, ½CH_2_), 4.33 (2H, s, CH_2_), 4.58–4.63 (2H, m, ½CH_2_ and NH), 6.60–6.66 (2H, M, ArH), 6.82 (1H, dd, *J* = 1.5, 8.4 Hz, ArH), 6.86–6.92 (2H, m, ArH), 6.95–7.06 (2H, m, ArH), 7.21–7.28 (3H, m, ArH), 7.39–7.44 (3H, m, ArH and NH). ^13^C NMR (CDCl_3_, 68 MHz): δ 21.6 (CH_3_), 28.6, 28.9, 41.0, (CH_2_), 44.1 (CH), 45.8, 47.6 (CH_2_), 112.0, 117.2, 118.5, 118.7, 118.8, 119.4, 123.2, 125.4 (ArCH), 127.7 (ArC), 129.4, 129.8 (ArCH), 129.8, 138.1, 140.2, 140.5 (ArC), 169.0, 172.3 (CO). HRMS: Calcd. for C_27_H_28_ClN_3_O_3_ (M + H)^+^ 478.1892, found (M + H)^+^ 478.1878.

##### *N*-(2-Acetyl-phenyl)-acetamide

A solution of 2-aminoacetopheone (2.0 g, 14.8 mmol) in DCM (80 mL) was cooled to 0 °C, and TEA (2.4 mL) and acetyl chloride (2.06 mL, 30 mmol) were added. The resulting solution was stirred at r.t. for 30 min. NaHCO_3_ was added and the solution was extracted; the organic layers were then washed with HCl (1M) and brine. The organic layers were dried (MgSO_4_), filtered and evaporated in vacuo to yield the desired product as a brown solid, 2.3 g, 89% yield. R.f. 0.49 (EtOAc), m.p. 68–70 °C, LCMS: *t*_r_ = 1.32 min (80% MeOH in water), m/z M-H 175.79, HPLC: *t*_r_ = 1.60 min (90% acetonitrile in H_2_O), 94%, ^1^H NMR (CDCl_3_, 270 MHz): δ 2.15 (3H, s, CH_3_), 2.58, (3H, s, CH3), 6.99–7.06 (1H, m, ArH), 7.43–7.49 (1H, m, ArH), 7.80 (1H, dd, *J* = 1.5, 8.15 Hz, ArH), 8.65 (1H, dd, *J* = 1.0, 8.4 Hz, ArH), 11.63 (1H, br.s, NH). ^13^C NMR (CDCl_3_, 68 MHz): δ 25.7, 28.8 (CH3), 120.8 (ArCH), 121.7 (ArC), 122.4, 131.7, 135.3 (ArCH), 141.1 (ArC), 169.6, 202.9 (CO). HRMS: Calcd. for C_10_H_11_NO_2_ (M + H)^+^ 178.0863, found (M + H)^+^ 178.0858.

##### *N*-(2–1-[2-(4-Chloro-phenoxy)-phenylamino]-ethyl-phenyl)-ethylamine (**25**)

A solution of 2-(4-chloro-phenoxy)-phenylamine (298 mg, 1.4 mmol), *N*-(2-acetyl-phenyl)-acetamide, (200 mg, 1.13 mmol) and chloro-tri-isopropoxy-titanium IV (0.53 mL, 2.26 mmol) in toluene (15 mL) was stirred at ambient temperature for 4 days. NaHCO_3_ was added and the mixture was extracted with EtOAc, dried (MgSO_4_) and evaporated to dryness. The residue was redissolved in THF (20 mL) and cooled to 0 °C, succinic acid (270 mg, 2.26 mmol) and borane (1M in THF, 2.3 mL, 2.26 mmol) were added. The reaction was slowly warmed to r.t. and stirred for 8h. NaHCO_3_ was added, and the volatile solvents removed in vacuo; the mixture was then extracted with EtOAc and dried (MgSO_4_). The crude material was purified by flash chromatography (0–100% DCM in hexane) to yield the product as an oil, 79 mg, 19% yield. LCMS: *t*_r_ = 1.42 min (95% MeOH in water), m/z M-H 365.33, HPLC: *t*_r_ = 4.49 min (90% acetonitrile in water), 97%, ^1^H NMR (CDCl_3_, 270 MHz,): δ 1.17 (3H, t, *J* = 7.2 Hz, CH_3_CH_2_), 1.56 (3H, d, *J* = 6.7 Hz, CH_3_CH), 3.10 (2H, q, *J* = 14.1 Hz, CH_2_), 4.22 (1H, d, *J* = 6.0 Hz, NH), 4.53 (1H, q, *J* = 13.3 Hz, CH), 4.59 (1H, br.s, NH), 6.66–6.93 (7H, m, ArH), 7.01 (1H, td, *J* = 7.9, 1.5 Hz, ArH), 7.16–7.31 (4H, m, ArH). ^13^C NMR (CDCl_3_, 68 MHz): 14.9, 19.9 (CH_3_), 38.1 (CH_2_), 50.9 (CH), 111.1, 113.6, 117.0, 118.0, 118.6, 119.5, 125.5, 126.5 (ArCH), 128.6, 127.8 (ArC), 128.3, 129.7 (ArCH), 13.7, 143.3, 146.7, 156.4 (ArC). HRMS: Calcd. for C_22_H_23_ClN_2_O (M + Na)^+^ 389.1386, found (M + Na)^+^ 389.1391.

##### *N*-(2-(1-[2-(4-Chloro-phenoxy)-phenylamino]-but-2-enyl)-phenyl)-acetamide (**26**)

*N*-(2-([2-(4-Chloro-phenoxy)-phenylimino]-methyl)-phenyl)-acetamide (500 mg, assumed 100% pure, 1.4 mmol) was dissolved in THF (15 mL) and cooled to 0 °C under a N_2_ atmosphere, BF_3_OEt_2_ (0.18 mL, 1.4 mmol) and allyl magnesium bromide (1 M in ether, 4.2 mL, 4.2 mmol) were added. The resulting solution was stirred at r.t. for 18 h. The reaction was then quenched with sat. NH_4_Cl solution then extracted with EtOAc and dried (MgSO_4_). The crude product was purified by flash chromatography (0–50% EtOAc in hexane) to yield the desired product as a light brown solid, 320 mg, 57% yield. R.f. 0.36 (DCM), LCMS: *t*_r_ = 1.59 min (95% MeOH in water), m/z M + H (+Na) 429.13, M + H 407.15, HPLC: *t*_r_ = 2.45 min (90% acetonitrile in water), 92%, HRMS: Calcd. for C_24_H_23_ClN_2_O_2_ (M + H)^+^ 407.1521, found (M + H)^+^ 407.1503. ^1^H NMR (CDCl_3_, 400 MHz,): δ 1.86 (3H, s, CH_3_), 2.54–2.62 (2H, m, CH_2_), 4.31 (1H, t, *J* = 6.4 Hz, CHNH), 4.60 (1H, s, NH), 5.10 (1H, s, ½CH_2_CH), 5.13 (1H, d, *J* = 5.2 Hz, ½CH_2_CH), 5.65–5.75 (1H, m, CHCH_2_), 6.66 (1H, d, *J* = 8.0 Hz, ArH), 6.77 (1H, t, *J* = 8.0 Hz, ArH), 6.87–6.96 (4H, m, ArH), 7.13 (1H, t, *J* = 7.6 Hz, ArH), 7.26–7.31 (4H, m, ArH), 8.05 (1H, d, *J* = 8.4 Hz, ArH), 9.40 (1H, br.s, NHCO). ^13^C NMR (CDCl_3_, 101 MHz): 24.3 (CH_3_), 40.6 (CH), 58.9 (CH_2_), 114.9, 118.0 (ArCH), 119.3 (CH_2_), 119.8, 120.0, 123.1, 124.7, 125.6 (ArCH), 128.0 (ArC), 128.2, 128.2, 129.8 (ArCH), 130.7 (ArC), 134.0 (CH), 136.9, 139.3, 143.5, 156.1 (ArC), 168.1 (CO).

##### *N*-(2-(1-[2-(4-Chloro-phenoxy)-phenylamino]-2-phenyl-ethyl)-phenyl)-acetamide (**27**)

*N-*(2-([2-(4-Chloro-phenoxy)-phenylimino]-methyl)-phenyl)-acetamide (111 mg, assumed 100% pure, 0.3 mmol) was dissolved in THF (5 mL) and cooled to 0 °C under a N_2_ atmosphere, BF_3_OEt_2_ (0.04 mL, 0.3 mmol) and benzyl magnesium bromide (2 M in THF, 0.3 mL, 1.2 mmol) were added. The resulting solution was stirred at r.t. for 18 h. The reaction was then quenched with sat. NH_4_Cl solution, extracted with EtOAc and dried (MgSO_4_). The crude product was purified by flash chromatography (0–20% EtOAc in DCM) to yield the desired product as an oil, 20 mg, 14% yield. R.f. 0.25 (DCM), LCMS: *t*_r_ = 1.26 min (95% MeOH in water), m/z M^-^H 455.15, HPLC: *t*_r_ = 2.73 min (90% acetonitrile in water), 97%, ^1^H NMR (CDCl_3_, 400 MHz,): δ 1.82 (3H, s, CH_3_), 3.02–3.04 (2H, m, CH_2_), 4.43 (1H, t, *J* = 8.0 Hz, CH), 4.50 (1H, br.s, NH), 6.52 (1H, d, *J* = 6.8 Hz, ArH), 6.63–6.74 (4H, m, ArH), 6.79–6.84 (1H, m, ArH), 6.98–7.00 (1H, m, ArH), 7.05 (1H, t, *J* = 7.2 Hz, ArH), 7.15–7.24 (7H, m, ArH), 7.91 (1H, d, *J* = 8.4 Hz, ArH), 8.90 (1H, br.s, NHCO). ^13^C NMR (CDCl_3_, 101 MHz): δ 24.3 (CH_3_), 42.9 (CH_2_), 60.8 (CH), 114.9, 117.9, 119.6, 119.7, 123.4, 124.9, 125.6, 127.2 (ArCH), 127.9 (ArC), 128.1, 128.2, 128.9, 129.1, 129.8 (ArCH), 131.2, 136.7, 136.8, 139.3, 143.4, 156.0 (ArC), 168.2 (CO). HRMS: Calcd. for C_28_H_25_ClN_2_O_2_ (M + H)^+^ 457.1677, found (M + H)^+^ 457.1666.

##### *N*-(2-(1-[2-(4-Chloro-phenoxy)-phenylamino]-butyl)-phenyl)-acetamide (**28**)

To a solution of *N*-(2-(1-[2-(4-chloro-phenoxy)-phenylamino]-but-2-enyl)-phenyl)-acetamide, (50 mg, 0.12 mmol) in EtOAc (25 mL) had Pd/C (15 mg) added. The solution was then stirred under a H_2_ atmosphere for 15 min and filtered through Celite. Purification by flash chromatography (0–50% EtOAc in hexane) afforded the desired product, 44 mg, 88% yield. R.f. 0.42 (EtOAc), LCMS: *t*_r_ = 3.72 min (90% MeOH in water), m/z M + H 409.00, HPLC: *t*_r_ = 4.69 min (90% MeOH in water), 99%, ^1^H NMR (CDCl_3_, 400 MHz,): δ 0.90 (3H, t, *J* = 7.6 Hz, CH_2_CH_3_), 1.21–1.39 (2H, m, CH_2_), 1.79–1.86 (2H, m, CH_2_), 1.87 (3H, s, CH_3_CO), 4.29 (1H, t, *J* = 7.6 Hz, CH), 4.37 (1H, br.s, NH), 6.70 (1H, d, *J* = 8.0 Hz, ArH), 6.73–6.78 (1H, m, ArH), 6.85–6.87 (1H, m, ArH), 6.90–6.96 (3H, m, ArH), 7.11 (1H, t, *J* = 7.2 Hz, ArH), 7.25–7.31 (4H, m, ArH), 8.05 (1H, d, *J* = 8.0 Hz, ArH), 9.36 (1H, br.s, NHCO). ^13^C NMR (CDCl_3_, 101 MHz): 13.7 (CH_3_CH_2_), 19.6 (CH_2_), 24.4 (CH_3_CO), 38.0 (CH_2_), 60.0 (CH), 114.7, 118.3, 119.4, 119.5, 123.0, 124.4, 125.4, 127.9, 128.1, 128.5, 129.9 (ArCH), 130.9, 136.8, 139.3, 143.7, 156.0 (ArC), 168.1 (CO). HRMS: Calcd. for C_24_H_25_ClN_2_O_2_ (M + H)^+^ 409.1677, found (M + H)^+^ 409.1677.

##### *N*-(2-(1-[2-(4-Chloro-phenoxy)-phenylamino]-ethyl)-phenyl)-acetamide (**29**)

A cerium chloride suspension was prepared. CeCl_3_.7H_2_O (stored in the oven, 515 mg, 1.38 mmol) was heated under high vacuum for 15 min and allowed to cool to r.t., and then to 0 °C in an ice bath. To this THF (3 mL) and methyl magnesium bromide (3 M in diethyl ether, 0.46 mL, 1.38 mmol) were added, this was then stirred at r.t. for 2 h. To this *N*-(2-([2-(4-chloro-phenoxy)-phenylimino]-methyl)-phenyl)-acetamide intermediate (166 mg, 0.46 mmol) was added and stirred at r.t. for a further 18 h. NaHCO_3_ was added and the mixture was extracted with EtOAc, dried (MgSO_4_) and purified by flash chromatography (0–100% DCM) to yield the desired product 14 mg, 8% yield. R.f. 0.56 (DCM with TEA), LCMS: *t*_r_ = 1.08 min (90% MeOH in water), m/z M + Na 403.20, HPLC: *t*_r_ = 2.6 min (90% acetonitrile in water), 94%, ^1^H NMR (CDCl_3_, 270 MHz,): δ 1.55 (3H, d, *J* = 6.6 Hz, CH_3_CH), 1.90 (3H, s, CH_3_CO), 4.25 (1H, d, *J* = 3.0 Hz, CHNH), 4.52–4.54 (1H, m, CH), 6.73–6.78 (2H, m, ArH), 6.84–6.99 (4H, m, ArH), 7.11 (1H, t, *J* = 7.7 Hz, ArH), 7.24–7.31 (4H, m, ArH), 8.02 (1H, d, *J* = 8.0 Hz, ArH), 9.16 (1H, br.s, NH). ^13^C NMR (CDCl_3_, 101 MHz): 21.5, 24.4 (CH_3_), 53.9 (CH), 114.6, 118.5, 119.4, 119.6, 123.2, 124.7, 125.4, 127.3, 128.1 (ArCH), 128.2 (ArC), 129.9 (ArCH), 132.0, 136.8, 138.9, 143.9, 155.9 (ArC), 168.2 (CO). HRMS: Calcd. for C_22_H_21_ClN_2_O_2_ (M + H)^+^ 381.1364, found (M + H)^+^ 381.1352.

##### *N*-(4-(1-[2-(4-Chloro-phenoxy)-phenylamino]-ethyl)-phenyl)-*N*-ethyl-acetamide (**30**)

*N*-(4-(1-[2-(4-Chloro-phenoxy)-phenylamino]-ethyl)-phenyl)-ethane (50 mg, 0.14 mmol) was dissolved in DCM (1 mL) and cooled to 0°C, acetyl chloride (0.04 mL, 0.56 mmol) and TEA (0.02 mL, 0.42 mmol) were added. This was then allowed to warm to room temperature and stirred for 1h. Saturated NaHCO_3_ solution was added, and the mixture was extracted with DCM, dried (MgSO_4_) and purified by flash chromatography to yield the title compound as an off-white oil, 23 mg, 40% yield. R.f. 0.55 (EtOAc), LCMS: *t*_r_ = 1.37 min (95% MeOH in water), m/z M-H 407.34, HPLC: *t*_r_ = 3.03 min (90% acetonitrile in H_2_O), 96%, ^1^H NMR (CDCl_3_, 270 MHz,): δ 1.15 (3H, dt, *J* = 6.8, 10.4 Hz, CH_3_CH_2_), 1.43 (3H, dd, *J* = 6.4, 15.6 Hz, CH_3_CH), 1.74 (3H, d, *J* = 25.6 Hz, CH_3_CO), 3.07–3.15 (1H, m, ½CH_2_), 4.24–4.36 (2H, m, ½CH_2_ and NH), 4.64–4.75 (1H, m, CH), 6.51 (1H, dd, *J* = 1.6, 8.4 Hz, ArH), 6.57–6.67 (2H, m, ArH), 6.74–6.84 (2H, m, ArH), 6.86–6.90 (1H, m, ArH), 6.92–6.97 (1H, m, ArH), 7.06 (1H, td, *J* = 1.6, 8.0 Hz, ArH), 7.21–7.35 (4H, m, ArH), 7.39–7.47 (1H, m, ArH). ^13^C NMR (CDCl_3_, 68 MHz): 12.8, 22.5, 23.1 (CH_3_), 43.5 (CH_2_), 47.5 (CH), 112.7, 117.7, 118.8, 119.4, 125.3, 126.9, 128.3, 129.4, 129.7, 130.3 (ArCH), 138.9, 139.9, 141.7, 142.4, 143.1, 156.3 (ArC), 170.5 (CO). HRMS: Calcd. for C_24_H_25_ClN_2_O_2_ (M + Na)^+^ 431.1497, found (M + Na)^+^ 431.1487.

##### 1-Bromo-2-phenoxy-benzene

A mixture of 2-bromophenol (0.211 mL, 2 mmol), phenylboronic acid (490 mg, 4 mmol), copper acetate (364 mg, 2 mmol), TEA (1.38 mL, 10 mmol) and 4Å molecular sieves in DCM (25 mL) was stirred at r.t. for 18 h. The slurry was filtered through Celite and concentrated in vacuo. This was then diluted with EtOAc and NaHCO_3_ solution, extracted and the organic portions were washed with brine and dried (MgSO_4_). The crude mixture was purified by flash chromatography (hexane) to yield the desired product as a colourless oil, 232 mg, 47% yield. R.f. 0.75 (DCM), LCMS: *t*_r_ = 1.3 min (95% MeOH in water), m/z M-H 246.84, 248.86, HPLC: *t*_r_ = 2.88 min (90% acetonitrile in water), 98%, ^1^H NMR (CDCl_3_, 270 MHz,): δ 6.95–7.04 (4H, m, ArH), 7.11 (1H, td, *J* = 1.1, 8.0 Hz, ArH), 7.22–7.37 (3H, m, ArH), 7.61–7.65 (1H, m, ArH). ^13^C NMR (CDCl_3_, 68 MHz): 115.0 (ArC), 118.2, 120.7, 123.5, 125.1, 128.8, 129.9, 133.9 (ArCH), 153.8, 156.9 (ArC).

##### 1-Bromo-2-phenoxy-4′-chlorobenzene

A mixture of 2-bromophenol (0.2 mL, 2 mmol), phenylboronic acid (600 mg, 4 mmol), copper acetate (350 mg, 2 mmol), TEA (1.4 mL, 10 mmol) and powdered 4 Å molecular sieves (~2 g) in DCM (25 mL) was stirred at r.t. for 18 h. The slurry was filtered through Celite and concentrated in vacuo. This was then diluted with EtOAc and NaHCO_3_ solution, extracted and the organic portions were washed with brine and dried (MgSO_4_). The crude mixture was purified by flash chromatography (hexane) to yield the desired product as a colourless oil, 320 mg, 59% yield. R.f. 0.72 (DCM), HPLC: *t*_r_ = 3.29 min (90% acetonitrile in water), >99%, ^1^H NMR (CDCl_3_, 270 MHz,): δ 6.85–6.91 (1H, m, ArH), 6.96 (1H, dd, *J* = 1.4, 8.0 Hz, ArH), 7.00–7.07 (1H, m, ArH), 7.25–7.29 (3H, m, ArH), 7.62 (1H, dd, *J* = 1.6, 8.0 Hz, ArH).

##### 1-(2-Nitro-phenyl)-ethanone-*O*-methyl-oxime

To a solution of 2-nitroacetophenone (1.9 g, 10.9 mmol), methoxyamine hydrochloride (0.96 g, 10.9 mmol) in anhydrous pyridine (38 mL) and anhydrous EtOH (38 mL), powdered 4Å molecular sieves (~1 g) were added. The resulting mixture was heated at reflux for 3 h. The resulting mixture was filtered through Celite to remove the molecular sieves and then evaporated to dryness. The solid was redissolved in EtOAc and extracted with 20% NaHCO_3_ solution; this was then dried (MgSO_4_) and evaporated in vacuo to yield the desired compound as a mixture of enantiomers, yellow oil, 1.95 g, 87% yield. The product was used crude in following reactions. R.f. 0.55 (DCM), HPLC: *t*_r_ = 1.87 min, 58%, *t*_r_ = 2.39 min, 29% (90% acetonitrile in water), LCMS: *t*_r_ = 3.30 min, m/z M + H 195.4, *t*_r_ = 4.00 min, m/z M + H 195.3 (70% MeOH in water). ^1^H NMR (CDCl_3_, 400 MHz,): Major isomer: δ 2.54 (3H, s, CH_3_), 3.69 (3H, s, OCH_3_), 7.23–8.08 (4H, m, ArH). Minor isomer: δ 2.14 (3H, s, CH_3_), 3.94 (3H, s, OCH_3_), 7.23–8.08 (4H, m, ArH).

##### 1-(2-Nitro-phenyl)-ethylamine hydrochloride

A solution of 1-(2-nitro-phenyl)-ethanone-*O*-methyl-oxime (1.95 g, 10.05 mmol) in THF (7 mL) was cooled to 0 °C, borane/ THF complex (28 mL, 28.1 mmol) was added and the resulting solution was then heated at reflux for 6 h. The reaction was then cooled to −20 °C and water (2 mL) was added slowly followed by aq. 20% KOH solution (2 mL) over 20 min. The resulting mixture was then heated at reflux for a further 2 h and then poured into DCM. The mixture was then extracted with brine and dried (MgSO_4_). To form the salt, the product was redissolved in DCM and then concentrated HCl (1.5 mL) was added to the mixture and stirred for 1 h. The resulting solid was removed by filtration and washed with ether and dried, 345 mg, 17% yield. R.f. 0.32 (Hexane: DCM, 1:1), LCMS: *t*_r_ = 1.33 min (70% MeOH in water), m/z M + H 167.2 (free base), HPLC: *t*_r_ = 2.09 min (90% acetonitrile in water), >99%, ^1^H NMR (CDCl_3_, 400 MHz,): δ 1.60 (3H, d, *J* = 6.8 Hz, CH_3_), 4.77–4.80 (1H, m, CH), 7.63–7.67 (1H, m, ArH), 7.86 (1H, td, *J* = 1.2, 7.6 Hz, ArH), 8.03–8.05 (2H, m, ArH), 8.72 (2H, br, s, NH_2_).

##### (2-Phenoxy-phenyl)-(1-phenyl-ethyl)-amine

Palladium acetate (19 mg, 10 mol%) and rac-BINAP (51 mg, 10 mol%) were placed into an oven-dried flask; this was then evacuated and backfilled with N_2_. To this, 1-phenylethylamine (100 mg, 0.83 mmol), 1-bromo-2-phenoxy-benzene (185 mg, 0.74 mmol) and toluene (1 mL) were then added (via syringe). This was stirred for 10 min at r.t. Sodium *t*-butoxide (95 mg, 1 mmol) and further toluene (1 mL) were then added. The resulting solution was heated to reflux for 18 h. The mixture was then filtered through Celite and purified by flash chromatography (0–50% DCM in hexane) to yield the desired product as a pale cream oil, 104 mg, 49% yield. R.f. 0.45 (1:1, DCM: Hexane), LCMS: *t*_r_ = 2.18 min (90% MeOH in water), m/z M + H 290.10, HPLC: *t*_r_ = 3.11 min (90% acetonitrile in water), 98%, ^1^H NMR (CDCl_3_, 270 MHz,): δ 1.47 (3H, d, *J* = 6.3 Hz, CH_3_), 4.45–4.58 (2H, m, CH and NH), 6.46 (1H, dd, *J* = 1.1, 8.0 Hz, ArH), 6.56 (1H, td, *J* = 1.4, 7.9 Hz, ArH), 6.80–6.89 (2H, m, ArH), 6.99–7.11 (3H, m, ArH), 7.18–7.36 (7H, m, ArH). ^13^C NMR (CDCl_3_, 68 MHz): 25.2 (CH_3_), 53.3 (CH), 112.7, 116.7, 117.6, 119.2, 122.8, 124.8, 125.9, 126.9, 128.7, 129.8 (ArCH), 139.6, 143.0, 145.2, 157.7 (ArC). HRMS: Calcd. for C_20_H_19_NO (M + H)^+^ 290.1539, found (M + H)^+^ 290.1529.

##### [2-(4-Chloro-phenoxy)-phenyl]-(1-phenyl-ethyl)-amine

Palladium acetate (10 mg, 10 mol%) and rac-BINAP (26 mg, 10 mol%) were placed into an oven-dried flask, which was evacuated and backfilled with N_2_. To this was then added (via syringe) 1-phenylethylamine (50 mg, 0.42 mmol), 1-bromo-2-phenoxy-4′chlorobenzene (105 mg, 0.38 mmol) and toluene (1 mL). This was stirred for 10min at r.t. Sodium *t*-butoxide (50 mg, 0.5 mmol) and a further portion of toluene (1 mL) were then added. The resulting solution was heated to reflux for 3 h. This was then filtered through Celite and purified by flash chromatography (hexane) to yield the desired product as a white solid, 51 mg, 41% yield. R.f. 0.35 (hexane), m.p. 84–86 °C, LCMS: *t*_r_ = 4.67 min (90% MeOH in water), m/z M-H 322.3, HPLC: *t*_r_ = 4.30 min (90% acetonitrile in water), 97%, ^1^H NMR (CDCl_3_, 270 MHz,): δ 1.48 (3H, d, *J* = 6.0 Hz, CH_3_), 4.49–4.53 (2H, m, CH and NH), 6.48 (1H, t, *J* = 8.0 Hz, ArH), 6.92–6.95 (2H, m, ArH), 7.20–7.31 (7H, m, ArH). ^13^C NMR (CDCl_3_, 68 MHz): 25.2 (CH_3_), 53.3 (CH), 113.0, 116.9, 118.8, 119.3, 125.3, 125.9, 127.1 (ArCH), 127.8 (ArC), 128.8, 129.8 (ArCH), 139.5, 142.6, 145.1, 156.4 (ArC). HRMS: Calcd. for C_20_H_18_ClNO (M + H)^+^ 324.1150, found (M + H)^+^ 324.1136.

##### [2-(4-Chloro-phenoxy)-phenyl]-[1-(2-nitro-phenyl)-ethyl]-amine

Palladium acetate (15 mg, 10 mol%), rac-BINAP (45 mg, 10 mol%) and 1-(2-nitro-phenyl)-ethylamine hydrochloride (151 mg, 0.75 mmol) were placed into an oven-dried flask, which was evacuated and backfilled with N_2_. To this, 1-bromo-2-phenoxy-4′chlorobenzene (190 mg, 0.68 mmol) and toluene (2 mL) were then added (via syringe). This was stirred for 10 min at r.t. Sodium *t*-butoxide (195 mg, 2.04 mmol) and a further portion of toluene (2 mL) were then added. The resulting solution was heated to reflux for 24 h. The slurry was then filtered through Celite and purified by flash chromatography (0–100% DCM in hexane) to yield the desired product as a yellow oil, 120 mg, 48% yield. R.f. 0.45 (1:1, Hexane: DCM), LCMS: *t*_r_ = 3.85 min (90% MeOH in water), m/z M-H 367.50, ^1^H NMR (CDCl_3_, 400 MHz,): δ 1.51 (3H, d, *J* = 6.8 Hz, CH_3_), 5.16 (1H, q, *J* = 6.4 Hz, CH), 6.28 (1H, dd, *J* = 1.2, 7.6 Hz, ArH), 6.56 (1H, td, *J* = 1.2, 7.6 Hz, ArH), 6.76 (1H, dd, *J* = 1.6, 8.4 Hz, ArH), 6.82 (1H, td, *J* = 1.2, 7.2 Hz, ArH), 6.89–6.93 (2H, m, ArH), 7.24–7.28 (2H, m, ArH), 7.31–7.35 (1H, m, ArH), 7.47 (1H, td, *J* = 1.2, 7.6 Hz, ArH), 7.54 (1H, dd, *J* = 1.2, 8.0 Hz, ArH), 7.88 (1H, dd, *J* = 1.2, 8.4 Hz, ArH). ^13^C NMR (CDCl_3_, 101 MHz): δ 24.1 (CH_3_), 48.7 (CH), 112.3, 117.5, 118.7, 119.1, 124.7, 125.2, 127.3, 127.8 (ArCH), 127.9 (ArC), 129.7, 133.6 (ArCH), 138.4, 140.4, 142.6, 148.7, 156.1 (ArC).

##### [2-(4-Chloro-phenoxy)-phenyl]-[1-(2-amino-phenyl)-ethyl]-amine

Using the general procedure for the reduction of the substituted 2-nitrobenzylalcohol, but with a shortened reaction time of 10min at reflux, the product was isolated as a yellow oil, 12 mg, 25% yield. R.f. 0.32 (DCM), LCMS: *t*_r_ = 2.81 min (90% MeOH in water), m/z M-H 337.60, ^1^H NMR (CDCl_3_, 400 MHz,): δ 1.53 (3H, d, *J* = 6.8 Hz, CH_3_), 4.06 (2H, br.s, NH_2_), 4.21 (1H, br.s, NH), 4.54–4.56 (1H, m, CH), 6.63–6.67 (2H, m, ArH), 6.70–6.77 (2H, m, ArH), 6.81 (1H, dd, *J* = 0.8, 7.6 Hz, ArH), 6.87–6.91 (2H, m, ArH), 6.95–6.99 (1H, m, ArH), 7.07 (1H, td, *J* = 0.8, 7.2 Hz, ArH), 7.19 (1H, dd, *J* = 1.2, 7.6 Hz, ArH), 7.23–7.27 (2H, m, ArH). ^13^C NMR (C(ArC), 128.0, 129.6 (ArCH), 139.5, 143.1, 144.8, 156.2 (ArC).

##### *N*-[2-(1-Acetylpiperidin-4-ylamino)benzyl]-*N*-[2-(4-chlorophenoxy)phenyl]acetamide (**30**)

To a solution of [2-(4-chloro-phenoxy)-phenyl]-[1-(2-amino-phenyl)-ethyl]-amine (20 mg, 0.06 mmol) and TEA (0.008 mL) in DCM (1 mL) at 0 °C, acetyl chloride (0.009 mL) was added and the resulting solution was warmed to r.t. and stirred for 1 h. NaHCO_3_ was added and the mixture extracted with DCM; the organic layers were then washed with 1M HCl. The organic layers were combined and dried (MgSO_4_) and evaporated in vacuo. Purification by flash chromatography (0–10% MeOH in DCM) afforded the desired product as a pale cream oil, 22 mg, 96% yield.

##### *R*-(-)-*N*-(2-(1-[2-(4-Chloro-phenoxy)-phenylamino]-but-2-enyl)-phenyl)-acetamide (**31**)

m.p. 139–140 °C (from hexane/DCM), LCMS (Chiracel AD-H column): *t*_r_ = 11.5 min (80% MeOH in water), m/z M-H 405.2, HPLC (Chiracel AD-H column): *t*_r_ = 9.00 min (80% MeOH in water), >99%, [α]_D_ = −155.7, ^1^H NMR (CDCl_3_, 400 MHz,): δ 1.86 (3H, s, CH_3_), 2.54–2.62 (2H, m, CH_2_), 4.31 (1H, t, *J* = 6.4 Hz, CHNH), 4.60 (1H, s, NH), 5.10 (1H, s, ½CH_2_CH), 5.13 (1H, d, *J* = 5.2 Hz, ½CH_2_CH), 5.65–5.75 (1H, m, CHCH_2_), 6.66 (1H, d, *J* = 8.0 Hz, ArH), 6.77 (1H, t, *J* = 8.0 Hz, ArH), 6.87–6.96 (4H, m, ArH), 7.13 (1H, t, *J* = 7.6 Hz, ArH), 7.26–7.31 (4H, m, ArH), 8.05 (1H, d, *J* = 8.4 Hz, ArH), 9.40 (1H, br.s, NHCO). ^13^C NMR (CDCl_3_, 101 MHz): 24.3 (CH_3_), 40.6 (CH_2_), 58.9 (CH_2_), 114.9, 118.0 (ArCH), 119.3 (CH_2_), 119.8, 120.0, 123.1, 124.7, 125.6 (ArCH), 128.0 (ArC), 128.2, 128.2, 129.8 (ArCH), 130.7 (ArC), 134.0 (CH), 136.9, 139.3, 143.5, 156.1 (ArC), 168.1 (CO). Anal. Calcd. for C_24_H_23_ClN_2_O_2_. ½H_2_O C 69.31, H 5.82, N 6.74%. Found: C 68.9, H 5.75, N 6.50%. HRMS: Calcd. for C_24_H_23_ClN_2_O_2_ (M + H)^+^ 407.1521, found (M + H)^+^ 407.1503.

##### *S*-(+)-*N*-(2-(1-[2-(4-Chloro-phenoxy)-phenylamino]-but-2-enyl)-phenyl)-acetamide (**32**)

m.p. 139–141 °C (from hexane/DCM), LCMS (Chiracel AD-H column): *t*_r_ = 14.8 min (80% MeOH in water), m/z M-H 405.4, HPLC (Chiracel AD-H column): *t*_r_ = 11.50 min (90% acetonitrile in water), >99%, [α]_D_ = +158.0, ^1^H NMR: As **191**, ^13^C NMR: As **191**, Anal. Calcd. for C_24_H_23_ClN_2_O_2_. ½H_2_O C 69.31, H 5.82, N 6.74%. Found: C 69.7, H 5.74, N 6.75%. HRMS: Calcd. for C_24_H_23_ClN_2_O_2_ (M + H)^+^ 407.1521, found (M + H)^+^ 407.1502. DCl_3_, 101 MHz): 20.2 (CH_3_), 50.1 (CH), 113.3, 116.6, 117.7, 118.6, 118.7, 119.3, 125.3, 126.7 (ArCH), 127.5, 127.7.

## Figures and Tables

**Table 1:** Oxidation conditions investigated. ^a^ Isolated yield. ^b^ Only starting material isolated. ^c^ Incorrect product isolated, mono-chloro substitution occurred (Scheme 5).

Entry	Reagent/Conditions	Yield (%) ^a^
1	Trichloroisocyanuric acid, TEMPO, 0 °C to r.t. 10 min [24].	^c^
2	TEMPO, DCM, 3 d.	^b^
3	SWERN, (oxalyl chloride, DMSO, −50 °C, 2 h) [25].	53
4	MP-TsO-TEMPO, acetonitrile, 4 h.	66
5	Dess Martin Periodinane (DMP), [26,27] DCM, 10 min.	67
6	MnO_2_, DCM, 18 h.	71

**Table 2:**
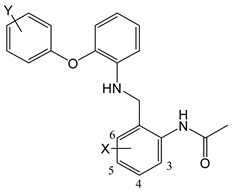
Compound list and biological data obtained for analogues based upon **1**, with substitutions around the central aromatic ring. ^a^ Results obtained from the TLC assay. Mean of at least two measurements with typically an SD of ±5%. IA (inactive). ^b^ Compound **4** was synthesised using a different route, see Scheme 2a. ^c^ Compound **1** was synthesised using a different route (see Scheme 1).

Compound No.	X	Y	% Inhibition at 10 μM ^a^
**1 ^c^**	H	4-Cl	87, IC_50_ = 0.9 μM
**4 ^b^**	4,5-Methylenedioxy	4-Cl	86, IC_50_ = 450 nM
**5**	4,5-diOMe	4-Cl	IA
**6**	4,5-diOMe	2,4-diCl	IA
**7**	Naphthyl	4-Cl	IA
**8**	5-Me	4-Cl	60–70
**9**	5-Cl	4-Cl	97, IC_50_ = 1.9 μM
**10**	5-Cl	2,4-diCl	IA
**11**	5-Cl	4-OCF_3_	IA

**Table 3:**
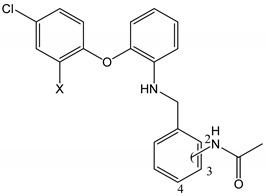
Biological data for compounds with a migrated *N*-acetamide group; ^a^ results obtained from the TLC assay. Mean of at least two measurements with typically an SD of ±5%.

Compound No.	2/3/4	X	% Inhibition at 10 μM ^a^
**1**	2	H	87, IC_50_ = 0.9 μM
**18**	4	H	29
**20**	3	H	53
**19**	4	Cl	IA

**Table 4:**
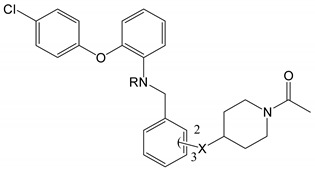
Biological results obtained for the extended analogues; ^a^ results obtained from the TLC assay. Mean of at least 2 measurements with typically an SD of ±5%. ^b^ Not determined.

Compound No.	R	X	2/3	% Inhibition at 10 μM ^a^	IC_50_ (μM)
**22**	Ac	NH	2	55	^b^
**21**	H	NH	2	36	^b^
**23**	H	NHCO	2	91	3.9
**24**	H	NHCO	3	97	1.1

**Table 5:**
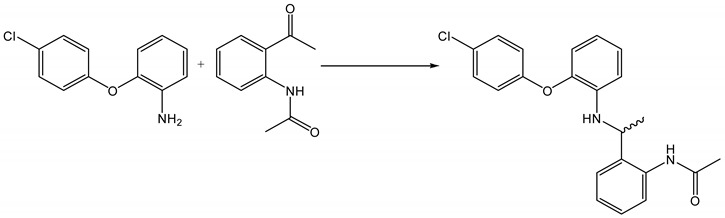
Reductive amination conditions investigated. SM, starting material.

Entry	Conditions	Result
1	Standard MW conditions [20], 140 °C, 30 min.	SM only
2	p-TSA, 4 Å MS, Dean–Stark reflux 18 h.	SM only
3	TiCl(O^i^Pr)_3_, r.t. 18 h, then cooled to −78 °C and NaBH_4_ added, 0 °C, 5 h [29].	Reduced amide (**25**)
4	TiCl(O^i^Pr)_3_, r.t. 2 d, then NaBH_4_ (2 eq.) and succinic acid (2 eq.) added, r.t. 3 h.	No product
5	TiCl(O^i^Pr)_3_, r.t. 2 d, then 1 M borane (2 eq.) and succinic acid (2 eq.) added, r.t. 3 h.	Reduced amide (**25**)
6	TiCl(O^i^Pr)_3_, r.t. 10 min, then NaBH(OAc)_3_, r.t. 6 h [29].	Reduced amide (**25**)
7	TiCl(O^i^Pr)_3_, DCM, r.t. 18 h.	No imine formation

**Table 6:** Grignard additions into imine intermediate.

Entry	Grignard Reagent	Conditions	Result
1	PhMgBr	BF_3_OEt_2_, r.t. 18 h	Imine isolated
2	IsopropylMgCl	BF_3_OEt_2_, r.t. 18 h	Imine isolated
3	CyclopropylMgBr	BF_3_OEt_2_, r.t. 18 h	Imine isolated
4	VinylMgBr	BF_3_OEt_2_, r.t. 18 h	Imine isolated
5	MeMgBr	BF_3_OEt_2_, r.t. 18 h	Imine isolated
6	MeMgBr	BF_3_OEt_2_, MW 100 °C, 10 min	Aniline SM isolated
7	MeMgBr	BF_3_OEt_2_, Reflux, 6 h	Aniline SM isolated
8	AllylMgBr	BF_3_OEt_2_, r.t. 18 h	57% isolated yield (**26**)
9	BnMgBr	BF_3_OEt_2_, r.t. 18 h	8% isolated yield (**27**)

**Table 7:** Investigations into possible alternatives to the Grignard procedure.

Entry	Conditions	Result
1	IsopropylMgCl, ZnCl_2_, r.t. 1 h, then imine added, r.t. 18 h [33].	Imine isolated
2	IsopropylMgCl, CuI, BF_3_OEt_2_, r.t. 18 h [34].	Imine isolated
3	Sc(OTf)_3_, MethylMgBr, r.t. 18 h [35].	Imine isolated
4	1,3-Bis(2,4,6-trimethylphenyl)-imidazolinium chloride, BuMgCl, r.t. 1 h, then imine added, r.t. 3 d [36].	No product
5	CeCl_3_, MeMgBr, r.t. 2 h, then imine added, then r.t. 18 h [37,38].	Product, **29**, 8%.

**Table 8:** Attempted procedures using organolithium reagents.

Entry	Conditions	Result
1	MeLi, −80 °C, 18 h, r.t. 5 h.	Imine isolated
2	Me_2_CuLi formed in situ. CuI, MeLi, −40 °C, 1 h, then imine added, −40 °C to r.t 18 h [39].	Imine isolated

**Table 9:** ^a^ Results obtained from the TLC assay. Mean of at least two measurements with typically an SD of ±5%. IA inactive.

Compound Number	% Inhibition at 10 μM ^a^ or IC_50_
**STX2171**	200 nM
**1**	IC_50_ = 900 nM
**2**	60–70
**3**	60–70
**4**	IC_50_ = 450 nM
**5**	IA
**6**	IA
**7**	IA
**8**	60–70
**9**	IC_50_ = 1.9 μM
**10**	IA
**11**	60–70
**14**	IC_50_ = 460 nM
**15**	60–70
**16**	IA
**17**	IA
**18**	29
**19**	IA
**20**	53
**21**	36
**22**	55
**23**	IC_50_ = 3.9 μM
**24**	IC_50_ = 1.1 μM
**25**	20–60
**26**	IC_50_ = 520 nM
**27**	IA
**28**	IC_50_ = 370 nM
**29**	IC_50_ = 76 nM
**30**	IC_50_ = 74 nM
**31**	lA
**32**	IC_50_ = 370 nM

**Table 10:** Selectivity of some 17β-HSD Type 3 inhibitors against 17β-HSD Type 1 and Type 2.

Compound No.	Inhibition of 17β-HSD Type 3 (IC_50_ nM)	Inhibition of 17β-HSD Type 1 (%, at 1 μM ^a^ or 10 μM ^b^)	Inhibition of 17β-HSD Type 2 (%, at 10 μM)
**SCH-451659** (Positive Control)	5	19.6 ^b^	18.4
**STX2171**	200	nd	20.3
**1**	900	1.3 ^a^	15.9
**23**	3900	−2.3 ^b^	-

^a^ Tested at 1 μM; ^b^ tested at 10 μM.

**Table 11:** Lack of significant androgenic and cytotoxic effects for lead compounds.

Compound	HSD3 Activity (IC_50_ nM)	Cytotoxicity IC_50_ (µM)	
		PC3 (AR-)	LNCaP (AR+)
**SCH-451659**	17.1	6.8	5.5
**STX2171**	200.0	22.0	15.7
**29**	76.0	18.3	14.6
**30**	74.0	19.6	11.3
**BMS Dibenzazocine**	8.6	>50.0	19.4

## Data Availability

All relevant data are available in the article or Supplementary Materials.

## Supplementary Materials

The following are available online. Section S1: IC_50_ for inhibition of 17β-HSD Type 3 activity by compound **1**; Section S2: Chiral separation of compounds **31** and **32**; Section S3: X-ray data for compound **31**. Crystallographic data for **31** have been deposited with the Cambridge Crystallographic Data Centre as supplementary publication CCDC 2088971. A copy can be obtained free of charge on application to CCDC, 12 Union Road, Cambridge CB2 1EZ, UK [Fax (+44)-1223-336033, e-mail: deposit@ccdc.cam.ac.uk.2088971.

## Sample Availability

Samples of some compounds may be available from the authors.
