# Supplementary material for: Substituted Aryl Benzylamines as Potent and Selective Inhibitors of 17β-Hydroxysteroid Dehydrogenase Type 3
Source: Molecules. 2021 Nov 26;26(23):7166. doi: 10.3390/molecules26237166 (PMC8659031; doi:10.3390/molecules26237166)
Supplement: Supplementary file 1 [file molecules-26-07166-s001.zip › molecules-1474026-supplementary final new.pdf]

# SUPPLEMENTARY INFORMATION

# Substituted Aryl Benzylamines as Potent and Selective Inhibitors of 17β-Hydroxysteroid Dehydrogenase Type 3

Nigel Vicker[1,†], Helen V. Bailey[1,†], Joanna M. Day[2], Mary F. Mahon[3], Andrew Smith[1], Helena J. Tutill[2], Atul Purohit[2] and Barry V. L. Potter[1,4,*]

[1] Medicinal Chemistry, Department of Pharmacy and Pharmacology and Sterix Ltd, University of Bath, Claverton Down, Bath BA2 7AY, UK

[2] Oncology Drug Discovery & Women's Health Group, Department of Endocrinology & M Metabolic Medicine & Sterix Ltd., Imperial College London, London W2 1NY, UK

[3] Department of Chemistry, University of Bath, Claverton Down, Bath BA2 7AY, UK

[4] Medicinal Chemistry & Drug Discovery, Department of Pharmacology, University of Oxford, Mansfield Road, Oxford, OX1 3QT, UK.

* Correspondence: barry.potter@pharm.ox.ac.uk

† Equal contribution.

**Section S1.** Inhibition of 17β-HSD Type 3 activity by compound **1**, ($IC_{50}$ = 0.9 μM)

**Section S2**. Chiral separation of compounds **31** and **32.**

**Section S3.** X-ray data for compound **31**.

**Section S1.** Inhibition of 17β-HSD Type 3 activity by compound **1**, ($IC_{50}$ = 0.9 µM)

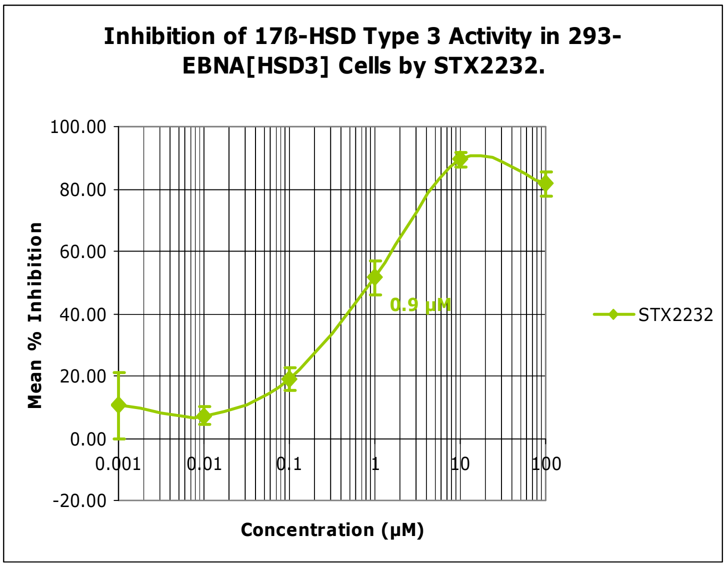


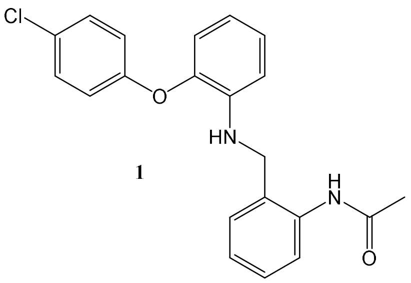


**1**

Figure S1. Inhibition of 17β-HSD Type 3 activity by compound **1**, ($IC_{50}$ = 0.9 µM)

**Section S2**. Chiral separation of racemic **26** to give enantiomers **31** and **32.**

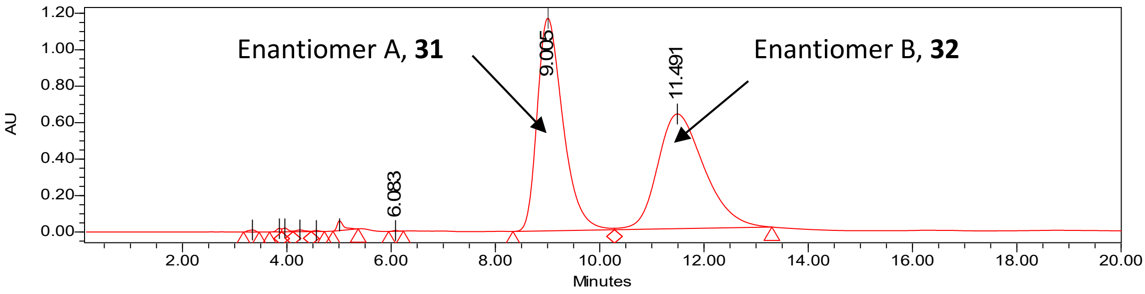


Figure S2. Chiral Separation of compound **26** to give enantiomers **31** and **32** using a Chiralcel AD-H chiral HPLC Column (80 % methanol and 20 % water at 1.0 mL/min).

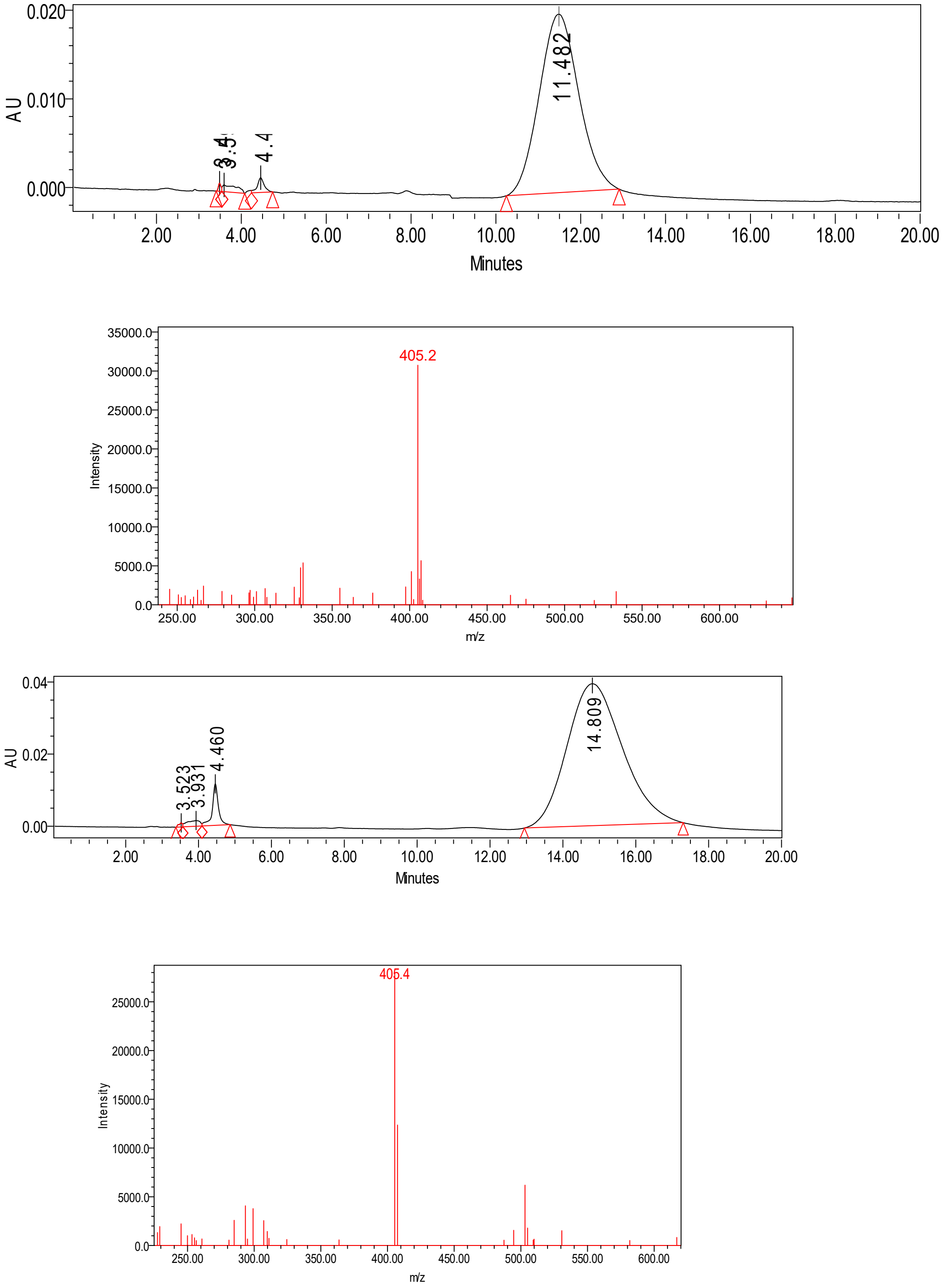


Figure S3. LCMS traces of Enantiomer A (**31**) and Enantiomer B (**32**) from the chiral separation the racemic compound **26** using a preparative Chiralcel AD-H chiral column (80 % methanol, 20 % water at 1.2 mL/min).

**Section S3.** X-ray data for compound **31**.

Table S1: Crystal data and structure refinement for k07farm3, compound **31**.

| | |
|---|---|
| Identification code | k07farm3 |
| Empirical formula | $C_{24}H_{23}ClN_2O_2$ |
| Formula weight | 406.89 |
| Temperature/K | 150.15 |
| Crystal system | orthorhombic |
| Space group | $P2_12_12_1$ |
| a/Å | 8.9500(1) |
| b/Å | 10.4450(1) |
| c/Å | 22.6800(2) |
| α/° | 90 |
| β/° | 90 |
| γ/° | 90 |
| Volume/Å$^3$ | 2120.19(4) |
| Z | 4 |
| $\rho_{calc}$g/cm$^3$ | 1.275 |
| μ/mm$^{-1}$ | 0.202 |
| F(000) | 856.0 |
| Crystal size/mm$^3$ | 0.35 × 0.25 × 0.25 |
| Radiation | MoKα (λ = 0.71073) |
| 2Θ range for data collection/° | 7.056 to 60.074 |
| Index ranges | $-12 \leq h \leq 12$, $-14 \leq k \leq 14$, $-31 \leq l \leq 30$ |
| Reflections collected | 42345 |
| Independent reflections | 6200 [$R_{int}$ = 0.0403, $R_{sigma}$ = 0.0264] |
| Data/restraints/parameters | 6200/2/272 |
| Goodness-of-fit on $F^2$ | 1.040 |
| Final R indexes [I>=2σ (I)] | $R_1$ = 0.0329, $wR_2$ = 0.0764 |
| Final R indexes [all data] | $R_1$ = 0.0388, $wR_2$ = 0.0796 |
| Largest diff. peak/hole / e Å$^{-3}$ | 0.20/-0.21 |
| Flack parameter | 0.000(13) |

**Crystal Data** for $C_{24}H_{23}ClN_2O_2$ ($A_r$ =406.89): orthorhombic, space group $P2_12_12_1$ (no. 19), $a$ = 8.9500(1) Å, $b$ = 10.4450(1) Å, $c$ = 22.6800(2) Å, $U$ = 2120.19(4) Å$^3$, $Z$ = 4, $T$ = 150 K, $\mu$(MoK$_\alpha$) = 0.202 mm$^{-1}$, $D_{calc}$ = 1.275 g cm$^{-3}$, 42345 reflections measured (7.056° ≤ 2θ ≤ 60.074°), 6200 unique ($R_{int}$ = 0.0403) which were used in all calculations. The final $R_1$ was 0.0329 ($I \geq 2\sigma(I)$) and $wR_2$ was 0.0796 (all data).

H1A and H2 (attached to N1 and N2, respectively, in the structure of **k07farm3**, were located and refined at a distance of 0.9Å from the parent nitrogen atoms. The gross structure is dominated by 1-dimensional, hydrogen-bonded polymers which propagate along the *a*-axis.

Table S2: Fractional Atomic Coordinates (×10[4]) and Equivalent Isotropic Displacement Parameters (Å[2]×10[3]) for k07farm3. $U_{eq}$ is defined as 1/3 of of the trace of the orthogonalised $U_{IJ}$ tensor.

| **Atom** | ***x*** | ***y*** | ***z*** | **U(eq)** |
|---|---|---|---|---|
| Cl1 | 13382.8(5) | 5596.5(5) | 6077.3(2) | 40.78(13) |
| O1 | 7750.0(14) | 4512.2(12) | 7316.4(5) | 31.3(3) |
| O2 | 7183.1(13) | 7843.6(12) | 10379.9(6) | 34.4(3) |
| N1 | 8651.4(16) | 4859.8(13) | 8464.9(6) | 25.5(3) |
| N2 | 9268.8(15) | 6827.1(13) | 10065.9(6) | 24.7(3) |
| C1 | 9448.2(18) | 5227.0(14) | 9001.4(7) | 24.3(3) |
| C2 | 10980.1(19) | 4564.5(15) | 8993.4(7) | 27.5(3) |
| C3 | 11963.0(19) | 4982.8(17) | 8494.9(8) | 31.9(4) |
| C4 | 12551(2) | 4214(2) | 8098.7(10) | 46.0(5) |
| C5 | 8614.9(19) | 4829.2(15) | 9555.5(7) | 26.3(3) |
| C6 | 7902(2) | 3644.8(18) | 9573.5(8) | 37.9(4) |
| C7 | 7206(3) | 3208.2(18) | 10080.4(9) | 44.3(5) |
| C8 | 7245(3) | 3947.0(18) | 10589.3(8) | 38.4(4) |
| C9 | 7942(2) | 5123.3(17) | 10583.1(7) | 29.8(3) |
| C10 | 8614.8(17) | 5579.4(15) | 10067.4(7) | 24.5(3) |
| C11 | 8502.2(18) | 7886.2(15) | 10219.5(7) | 25.0(3) |
| C12 | 9337.5(19) | 9135.6(16) | 10186.8(8) | 28.8(3) |
| C13 | 7475.1(17) | 5593.9(15) | 8248.0(7) | 25.0(3) |
| C14 | 6683(2) | 6477.9(16) | 8587.6(8) | 29.4(3) |
| C15 | 5513(2) | 7185.3(18) | 8344.4(9) | 35.4(4) |
| C16 | 5100(2) | 7017(2) | 7760.1(10) | 40.2(4) |
| C17 | 5869(2) | 6131.8(19) | 7417.4(9) | 36.4(4) |
| C18 | 7028.8(19) | 5443.8(16) | 7659.6(7) | 27.4(3) |
| C19 | 9074.7(19) | 4848.5(16) | 7041.5(7) | 26.2(3) |
| C20 | 9785(2) | 6019.5(16) | 7114.5(7) | 28.4(3) |
| C21 | 11124(2) | 6255.6(17) | 6817.7(8) | 30.3(3) |
| C22 | 11713(2) | 5320.8(17) | 6455.7(7) | 31.0(4) |
| C23 | 11008(2) | 4153.6(17) | 6382.9(8) | 34.1(4) |
| C24 | 9683(2) | 3912.9(17) | 6679.8(8) | 32.6(4) |

Table S3: Anisotropic Displacement Parameters (Å[2]×10[3]) for k07farm3. The Anisotropic displacement factor exponent takes the form: $-2\pi^2[h^2a^{*2}U_{11}+2hka^*b^*U_{12}+\ldots]$.

| **Atom** | $U_{11}$ | $U_{22}$ | $U_{33}$ | $U_{23}$ | $U_{13}$ | $U_{12}$ |
|---|---|---|---|---|---|---|
| Cl1 | 32.8(2) | 51.5(3) | 38.1(2) | 6.6(2) | 5.33(18) | 7.7(2) |
| O1 | 36.5(6) | 30.2(6) | 27.2(6) | -4.3(5) | 2.1(5) | -7.5(5) |
| O2 | 20.7(5) | 34.6(6) | 47.9(8) | -7.4(6) | 1.2(5) | 1.4(5) |

| | | | | | | |
|---|---|---|---|---|---|---|
| N1 | 29.2(7) | 25.9(6) | 21.5(6) | -1.8(5) | 1.5(5) | 1.9(5) |
| N2 | 20.6(6) | 27.6(6) | 26.0(6) | -4.1(5) | 1.0(5) | -0.3(5) |
| C1 | 30.6(7) | 22.1(7) | 20.4(7) | 0.0(6) | 0.8(6) | -1.5(6) |
| C2 | 30.8(7) | 25.1(7) | 26.8(7) | 1.9(6) | -1.5(6) | 1.2(6) |
| C3 | 29.3(8) | 30.2(8) | 36.2(9) | 2.1(7) | 2.1(7) | 0.3(7) |
| C4 | 45.6(11) | 44.3(11) | 48.2(11) | -4.0(9) | 15.5(9) | -0.4(9) |
| C5 | 32.8(8) | 24.3(7) | 21.9(7) | 1.4(6) | 1.7(6) | -1.1(6) |
| C6 | 59.6(12) | 27.0(8) | 27.2(8) | -2.2(7) | 8.8(8) | -9.1(8) |
| C7 | 69.0(14) | 30.4(9) | 33.3(9) | 1.4(7) | 13.7(10) | -14.3(9) |
| C8 | 53.4(12) | 35.6(9) | 26.3(8) | 4.6(7) | 9.6(8) | -4.4(8) |
| C9 | 32.8(8) | 34.1(8) | 22.6(7) | -1.0(6) | 1.1(6) | 1.7(7) |
| C10 | 23.9(7) | 25.8(7) | 23.7(7) | -0.1(6) | -0.5(5) | 0.8(6) |
| C11 | 22.5(7) | 28.8(8) | 23.8(7) | -3.4(6) | -4.1(6) | 1.6(6) |
| C12 | 26.6(7) | 28.3(8) | 31.6(8) | -2.8(6) | -1.4(7) | -0.1(6) |
| C13 | 25.4(7) | 23.7(7) | 26.0(7) | 2.9(6) | 2.0(6) | -4.2(6) |
| C14 | 28.9(8) | 29.2(8) | 30.2(8) | 0.2(6) | 5.6(7) | -2.5(7) |
| C15 | 29.0(9) | 31.4(8) | 45.9(10) | 2.8(8) | 8.2(8) | 1.9(7) |
| C16 | 27.2(8) | 40.9(10) | 52.5(12) | 11.0(9) | -2.5(8) | 1.5(8) |
| C17 | 32.8(9) | 41.7(10) | 34.8(9) | 6.4(8) | -6.7(8) | -6.0(8) |
| C18 | 28.3(7) | 27.1(8) | 26.6(7) | 0.8(6) | 1.2(6) | -5.3(6) |
| C19 | 31.4(8) | 28.6(8) | 18.6(7) | 1.0(6) | -2.9(6) | -0.4(6) |
| C20 | 34.4(8) | 27.1(8) | 23.7(7) | -1.9(6) | -0.3(6) | -0.2(6) |
| C21 | 34.5(8) | 28.6(8) | 27.8(8) | 1.4(7) | -1.3(7) | -0.5(7) |
| C22 | 29.4(8) | 37.6(9) | 26.0(7) | 5.6(7) | 0.1(6) | 6.6(7) |
| C23 | 41.1(9) | 30.7(9) | 30.4(8) | -2.8(7) | -0.7(7) | 8.5(7) |
| C24 | 41.5(9) | 27.0(8) | 29.3(8) | -2.3(6) | -4.3(7) | 0.5(7) |

Table S4: Bond Lengths for k07farm3.

| **Atom** | **Atom** | **Length/Å** | **Atom** | **Atom** | **Length/Å** |
|---|---|---|---|---|---|
| Cl1 | C22 | 1.7474(18) | C8 | C9 | 1.378(3) |
| O1 | C18 | 1.403(2) | C9 | C10 | 1.399(2) |
| O1 | C19 | 1.385(2) | C11 | C12 | 1.506(2) |
| O2 | C11 | 1.236(2) | C13 | C14 | 1.396(2) |
| N1 | C1 | 1.462(2) | C13 | C18 | 1.402(2) |
| N1 | C13 | 1.392(2) | C14 | C15 | 1.395(3) |
| N2 | C10 | 1.429(2) | C15 | C16 | 1.387(3) |
| N2 | C11 | 1.348(2) | C16 | C17 | 1.390(3) |
| C1 | C2 | 1.536(2) | C17 | C18 | 1.377(2) |
| C1 | C5 | 1.519(2) | C19 | C20 | 1.388(2) |

| | | | | | |
|---|---|---|---|---|---|
| C2 | C3 | 1.498(2) | C19 | C24 | 1.387(2) |
| C3 | C4 | 1.315(3) | C20 | C21 | 1.397(2) |
| C5 | C6 | 1.392(2) | C21 | C22 | 1.380(2) |
| C5 | C10 | 1.401(2) | C22 | C23 | 1.383(3) |
| C6 | C7 | 1.385(3) | C23 | C24 | 1.386(3) |
| C7 | C8 | 1.389(3) | | | |

Table S5: Bond Angles for k07farm3.

| **Atom** | **Atom** | **Atom** | **Angle/°** | **Atom** | **Atom** | **Atom** | **Angle/°** |
|---|---|---|---|---|---|---|---|
| C19 | O1 | C18 | 117.88(12) | N1 | C13 | C14 | 123.61(15) |
| C13 | N1 | C1 | 121.24(13) | N1 | C13 | C18 | 119.36(15) |
| C11 | N2 | C10 | 122.65(13) | C14 | C13 | C18 | 117.03(15) |
| N1 | C1 | C2 | 107.91(12) | C15 | C14 | C13 | 120.90(17) |
| N1 | C1 | C5 | 112.18(13) | C16 | C15 | C14 | 120.71(17) |
| C5 | C1 | C2 | 108.95(13) | C15 | C16 | C17 | 119.11(17) |
| C3 | C2 | C1 | 113.69(13) | C18 | C17 | C16 | 119.83(18) |
| C4 | C3 | C2 | 124.94(18) | C13 | C18 | O1 | 118.33(14) |
| C6 | C5 | C1 | 119.47(14) | C17 | C18 | O1 | 119.17(15) |
| C6 | C5 | C10 | 118.21(15) | C17 | C18 | C13 | 122.43(16) |
| C10 | C5 | C1 | 122.18(14) | O1 | C19 | C20 | 124.18(15) |
| C7 | C6 | C5 | 121.54(17) | O1 | C19 | C24 | 115.05(15) |
| C6 | C7 | C8 | 119.69(18) | C24 | C19 | C20 | 120.76(16) |
| C9 | C8 | C7 | 119.91(17) | C19 | C20 | C21 | 119.41(16) |
| C8 | C9 | C10 | 120.46(16) | C22 | C21 | C20 | 119.29(17) |
| C5 | C10 | N2 | 120.55(14) | C21 | C22 | Cl1 | 120.13(15) |
| C9 | C10 | N2 | 119.27(14) | C21 | C22 | C23 | 121.37(17) |
| C9 | C10 | C5 | 120.16(15) | C23 | C22 | Cl1 | 118.50(14) |
| O2 | C11 | N2 | 122.20(15) | C22 | C23 | C24 | 119.49(16) |
| O2 | C11 | C12 | 121.33(15) | C23 | C24 | C19 | 119.67(17) |
| N2 | C11 | C12 | 116.48(14) | | | | |

Table S6: Hydrogen Bonds for k07farm3.

| **D** | **H** | **A** | **d(D-H)/Å** | **d(H-A)/Å** | **d(D-A)/Å** | **D-H-A/°** |
|---|---|---|---|---|---|---|
| N2 | H2 | O2[1] | 0.898(7) | 1.921(7) | 2.8185(18) | 178(2) |

[1]1/2+X,3/2-Y,2-Z

Table S7: Hydrogen Atom Coordinates (Å×10[4]) and Isotropic Displacement Parameters (Å[2]×10[3]) for k07farm3.

| Atom | *x* | *y* | *z* | U(eq) |
|---|---|---|---|---|
| H1 | 9250(20) | 4560(20) | 8180(7) | 37(5) |
| H2 | 10206(12) | 6910(20) | 9930(9) | 38(6) |
| H1A | 9595 | 6176 | 9004 | 29 |
| H2A | 11497 | 4743 | 9370 | 33 |
| H2B | 10827 | 3628 | 8966 | 33 |
| H3 | 12178 | 5871 | 8462 | 38 |
| H4A | 12361 | 3320 | 8116 | 55 |
| H4B | 13165 | 4553 | 7795 | 55 |
| H6 | 7893 | 3124 | 9230 | 46 |
| H7 | 6703 | 2407 | 10080 | 53 |
| H8 | 6793 | 3642 | 10941 | 46 |
| H9 | 7967 | 5628 | 10931 | 36 |
| H12A | 8788 | 9735 | 9935 | 43 |
| H12B | 10333 | 8987 | 10020 | 43 |
| H12C | 9435 | 9498 | 10584 | 43 |
| H14 | 6943 | 6599 | 8990 | 35 |
| H15 | 4993 | 7788 | 8582 | 43 |
| H16 | 4303 | 7501 | 7596 | 48 |
| H17 | 5595 | 6002 | 7017 | 44 |
| H20 | 9364 | 6654 | 7364 | 34 |
| H21 | 11625 | 7051 | 6865 | 36 |
| H23 | 11427 | 3522 | 6131 | 41 |
| H24 | 9194 | 3111 | 6636 | 39 |

Crystal structure determination of [k07farm3]

Refinement model description. Number of restraints - 2, number of constraints - unknown. Details:

```
1. Fixed Uiso
 At 1.2 times of:
  All C(H) groups, All C(H,H) groups
 At 1.5 times of:
  All C(H,H,H) groups
2. Restrained distances
 H1-N1 = H2-N2
 0.9 with sigma of 0.005
3.a Ternary CH refined with riding coordinates:
 C1(H1A)
3.b Secondary CH2 refined with riding coordinates:
 C2(H2A,H2B)
3.c Aromatic/amide H refined with riding coordinates:
 C3(H3), C6(H6), C7(H7), C8(H8), C9(H9), C14(H14), C15(H15), C16(H16),
 C17(H17), C20(H20), C21(H21), C23(H23), C24(H24)
```

```
3.d X=CH2 refined with riding coordinates:
 C4(H4A,H4B)
3.e Idealised Me refined as rotating group:
 C12(H12A,H12B,H12C)
```

This report has been created with Olex2, compiled on 2018.05.29 svn.r3508 for OlexSys.

Crystallographic data for **31** been deposited with the Cambridge Crystallographic Data Centre as supplementary publication CCDC 2088971. A copy of these data can be obtained free of charge on application to CCDC, 12 Union Road, Cambridge CB2 1EZ, UK [fax (+44) 1223 336033, e-mail: deposit@ccdc.cam.ac.uk.2088971.
